# Plasmonic Single‐Molecule Affinity Detection at 10^−20^ Molar

**DOI:** 10.1002/adma.202418610

**Published:** 2025-01-23

**Authors:** Eleonora Macchia, Cinzia Di Franco, Cecilia Scandurra, Lucia Sarcina, Matteo Piscitelli, Michele Catacchio, Mariapia Caputo, Paolo Bollella, Gaetano Scamarcio, Luisa Torsi

**Affiliations:** ^1^ Dipartimento di Farmacia‐Scienze del Farmaco Università degli Studi di Bari Aldo Moro Bari 70125 Italy; ^2^ CNR IFN Bari 70125 Italy; ^3^ Dipartimento di Chimica Università degli Studi di Bari Aldo Moro Bari 70125 Italy; ^4^ Dipartimento Interateneo di Fisica Università degli Studi di Bari Aldo Moro Bari 70125 Italy; ^5^ Centre for Colloid and Surface Science Dipartimento di Chimica Università degli Studi di Bari Aldo Moro Bari 70125 Italy; ^6^ Faculty of Science and Engineering Åbo Akademi University Turku Finland; ^7^ NEST Istituto Nanoscienze – CNR and Scuola Normale Superiore Pisa I‐56127 Italy

**Keywords:** plasmonic sensors, single‐molecule sensing, single‐molecule with a large transistor (SiMoT), surface probing techniques, surface‐plasmon‐resonance

## Abstract

DNA can be readily amplified through replication, enabling the detection of a single‐target copy. A comparable performance for proteins in immunoassays has yet to be fully assessed. Surface‐plasmon‐resonance (SPR) serves as a probe capable of performing assays at concentrations typically around 10⁻⁹ molar. In this study, plasmonic single‐molecule assays for both proteins and DNA are demonstrated, achieving limits‐of‐detections (LODs) as low as 10⁻^2^⁰ molar (1 ± 1 molecule in 0.1 mL), even in human serum, in 1 h. This represents an improvement in typical SPR LODs by eleven orders‐of‐magnitude. The single‐molecule SPR assay is achieved with a millimeter‐wide surface functionalized with a physisorbed biolayer comprising trillions of recognition‐elements (antibodies or protein–probe complexes) which undergo an acidic or alkaline pH‐conditioning. Potentiometric and surface‐probing imaging experiments reveal the phenomenon underlying this extraordinary performance enhancement. The data suggest an unexplored amplification process within the biomaterial, where pH‐conditioning, driving the biolayer in a metastable state, induces a self‐propagating aggregation of partially misfolded proteins, following single‐affinity binding. This process triggers an electrostatic rearrangement, resulting in the displacement of a charge equivalent to 1.5e per 10^2^ recognition elements. Such findings open new opportunities for reliable SPR‐based biosensing at the physical detection limits, with promising applications in point‐of‐care plasmonic systems.

## Graphical abstract

Surface‐plasmon‐resonance (SPR) typically detects in the nanomolar range. A pH‐conditioning of a physisorbed biolayer, hosting trillions of capturing antibodies or probes enables SPR to detect a single protein or DNA‐strand in 0.1 mL of plasma. The pH‐conditioning transition the biolayer in a metastable state where a single affinity‐biding event can trigger an amplification mechanism involving a broad self‐propagating electrostatic rearrangement

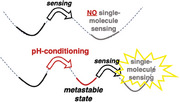

## 1 Introduction

Molecular assays are dominated by polymerase‐chain‐reaction based technologies, capable of amplifying hundred‐million‐fold a single copy of a nucleic acid marker in 0.1 mL (10^−20^ mol/L, M). No wonder they serve as a cornerstone in molecular diagnostics.^[^ ^1^ ^]^ While the relevance of ultrasensitive immunoassays is acknowledged, e.g. in enhancing diagnostic accuracy,^[^ ^2^ ^]^ no amplification phenomenon that enables single‐protein detection down to 10^−20^ m, namely in a range comparable to molecular assays, is known. Such limitation persists despite the potential for single‐molecule protein sequencing^[^ ^3^ ^]^ that, while technically achievable, remains impractical for rapid‐test assays. Digital beads‐based assays, typically operating in the 10^−15^ m range and seldom reaching 10^−19^ m,^[^ ^4^, ^5^ ^]^ entail four steps and results are ready in 5 h.

The detection of single proteins typically relies on near‐field approaches^[^ ^6^ ^]^ and their inherent spatial‐temporal confinement within a near‐field volume ranging from 10^−18^ to 10^−15^ L,^[^ ^7^ ^]^ such as within a microwell,^[^ ^8^ ^]^ a nanopore^[^ ^9^ ^]^ and detections can be performed with nanosensors^[^ ^10^ ^]^ or nanostructured‐based surface‐enhanced Raman methods,^[^ ^11^ ^]^ as well as with plasmonic nanodevices^[^ ^12^ ^]^ or nanometric field‐effect transistor (FET) based sensors.^[^ ^13^ ^]^ Generally, these approaches allow for an increase in the concentration of the target analyte while minimizing interference from other more abundant species present in an inevitably complex real biofluid. However, a significant challenge remains: the lowest concentration detected falls, generally, within the 10^−9^–10^−6^ m range. This is due to the diffusion barrier or concentration limit effects, which make it virtually impossible for a single molecule to reach a nanometric detecting interface by diffusing through a volume larger than 10^−15^ L.^[^ ^14^, ^15^ ^]^ Fluorescence‐based near‐field approaches can achieve limit‐of‐detections (LODs)—defined as ensuring a confidence level exceeding 99%,^[^ ^16^ ^]^ in a range as low as 10^−9^–10^−12^ m.^[^ ^15^ ^]^ Such LODs are enabled by the possibility to enhance the signal through increasing the fluorophore brightness while minimizing background light scattering. Even lower LODs can be reached by overcoming the diffusion barrier using fluorescence detection combined with the elicited digital beads‐based assays^[^ ^4^, ^8^ ^]^ that allow for wide‐field‐sampling.^[^ ^6^ ^]^ All these approaches achieve single‐molecule resolution but fail (most of them by many orders of magnitude) to reach a LOD of 10^−20^ m, corresponding to a single‐molecule in 0.1 mL. Last but not least, the ability to achieve extremely low LODs, even reaching down to 10^−20^ m,^[^ ^17^ ^]^ was demonstrated using large‐area transistor devices^[^ ^18^ ^]^ and Kelvin probe force microscopies,^[^ ^19^ ^]^ but the sensing mechanism remains elusive.

Surface‐plasmon‐resonance (SPR) allows to perform assays inspecting ultrathin layers of biological recognition elements deposited on a metalized slide. In standard SPR immunosensing assays, LODs are typically around 10^−9^ m.^[^ ^20^, ^21^ ^]^ When immunosensing involves plasmon‐enhanced effects such as in localized or fiber‐optic SPR, the LOD can decrease to 10^−12^ or 10^−13^ m (Table S1, Supporting Information). Seldom LODs of 10^−16^ m can be achieved, but only through the use of complex plasmonic nanosensors, even involving a nanoparticle release.^[^ ^22^ ^]^

Here, a selective plasmonic single‐molecule protein and DNA assay, performed directly on a bare physisorbed layer of capturing antibodies or probes, is proposed. The assay can detect, in about 1 h, at LODs of 10^−20^ m even in serum, which is at least four orders‐of‐magnitude lower than state‐of‐the‐art methods and 11 orders‐of‐magnitude better than standard SPR assays entailing no plasmonic enhancement strategies.

These results are corroborated by an ensemble of electronic, surface‐probing, and imaging techniques (e.g., electrolyte‐gated transistor sensors, *ς*‐potential, Kelvin probe force microscopies, and static contact angle) that concur to reveal the critical role of pH‐conditioning of the millimeter large detecting surface populated by trillions of highly packed capturing antibodies or protein–probe complexes, serving as recognition elements for target antigens and DNA stands, respectively. A phenomenological amplification sensing mechanism, arising from pH‐induced partial unfolding of the protein‐based recognition elements, ultimately exposing some of their hydrophobic regions, is supported by the experiments. Such a pH‐conditioning, triggering an aggregation process^[^ ^23^ ^]^ through short‐range hydrophobic interactions,^[^ ^24^ ^]^ drives the proteins in the biomaterial, in a metastable state. Strikingly, the structural modifications start from a single‐affinity couple conformational electrostatic change and entails a spreading over at least hundreds of millions of the packed recognition elements, enabling reliable detections down to 10^−20^ m. The mechanism is general and allows to explain not only the data acquired with SPR detection but also those obtained through potentiometric, contact angle, and imaging techniques.

## 2 Results and Discussion

### 2.1 pH‐Activated Plasmonic “Single/Few‐Molecules” Affinity Sensing

**Figure 1:**
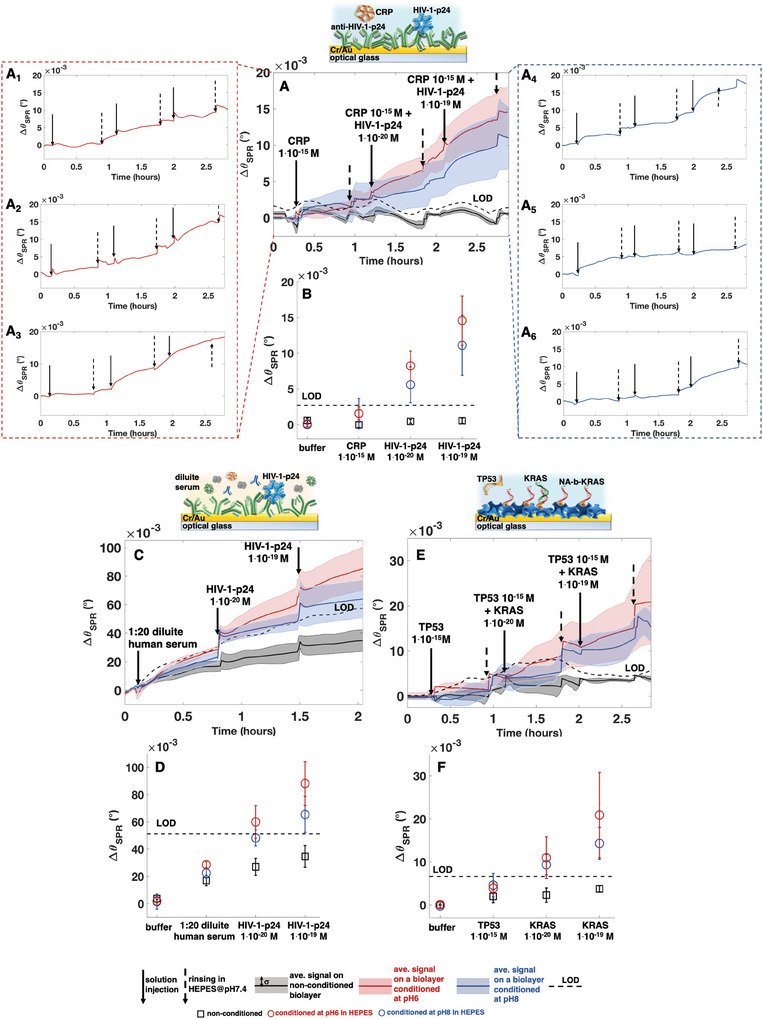
SPR “single/few‐molecules” detections of two antigens and a DNA strand in a physiological buffer and in diluted human serum—sensograms (Δ*θ*_SPR_ vs. time) of pH‐conditioned biolayers (red lines: pH6, blue lines: pH8) at each sensing step; data on a nonconditioned biolayer is shown for comparison (black lines). The colored shadings represent one standard deviation, while the dashed black lines are the LOD levels, calculated as the average values of each black curve (taken as the measure of the noise) plus three times the standard deviation. All the solutions are in an HEPES@pH7.4 buffer (*i*_s_ = 150 × 10^−3^ m) or in diluted human serum. The solid arrows indicate the injection of 1 mL of a given solution, while the rinsing steps are indicated by dashed arrows. A) Sensogram of an anti‐HIV‐1‐p24 capturing layer exposed to the nonbinding CRP (1 × 10^−15^ m) and to HIV‐1‐p24 (1 × 10^−20^ m, 1 × 10^−19^ m, six replicates, raw‐data in Figures S7–S9, Supporting Information). Panels 1A_1 –_ 1A_6_ present data from three independent replicated experiments, with their averages and standard deviations shown in Figure 1A,B) Δ*θ*_SPR_ respect to *θ*_SPR0_ data, with *θ*_SPR0_ measured in plain buffer, relevant to panel (A); black squares are for the nonconditioned biolayers, red circles and blue circles for biolayer conditioned at pH6 and pH8, respectively. C,D) same as in (A) and (B), respectively, but in 1:20 diluted pooled human serum (four replicates, raw data in Figures S10–S12, Supporting Information). E) NA‐b‐KRAS probes exposed to the nonbinding TP53 (1 × 10^−15^ m) and to KRAS (1 × 10^−20 ^and 1 × 10^−19^ m, four replicates, raw data in Figures S17–S19, Supporting Information). F) *Δθ*_SPR_ shifts of data in panel (E).

In **Figure** **1**, the transient SPR signals on pH‐conditioned capturing or protein–probe complex biolayers, are shown. The same curves on nonconditioned biomaterials are shown for comparison. The generality of this study is established through the systematic evaluation of three different biosystems, namely: (i) an anti‐HIV‐1‐p24 capturing layer designed to detect the p24 antigen of the HIV‐1 virus capsid, with the nonbinding C‐reactive protein (CRP) serving as interferent in negative control experiments; (ii) an anti‐Immunoglobulin G (anti‐IgG) layer for detecting IgG, with IgM as nonbinding species; (iii) a biolayer (or biomaterial) of protein–probe complexes comprising neutravidins (NAs) coupled to biotinylated complementary KRAS strands, named b‐KRAS. The whole protein–probe layer is addressed as NA‐b‐KRAS. The KRAS mutated gene, used as a marker for pancreatic cancer precursors,^[^ ^25^ ^]^ is the target, while TP53, another mutated gene, serves as nonaffinity target in negative control experiments.

SPR reflectance intensity goes through a dip at the Δ*θ*_SPR_ shift of the laser incident angle *θ*, corresponding to the energy‐wavevector conservation between the exciting photons and the surface plasmon polaritons^[^ ^26^ ^]^ (Figure S1, Note S1, Supporting Information). The transient Δ*θ*_SPR_ shifts, addressed as sensograms that are measured in situ and operando, of the anti‐HIV‐1‐p24, anti‐IgG or NA‐b‐KRAS biolayers physisorption on a millimeter large slide (0.4 cm^2^), are shown in Figures S2–S4 (Supporting Information). The deposition occurs in a 4‐(2‐hydroxyethyl)‐1‐piperazineethanesulphonic acid (HEPES) buffer at pH7.4 and ionic strength, *i*_s_, of 150 × 10^−3^ m (HEPES@pH7.4), mimicking physiological conditions. The process leads to biomaterials formed of stably adherent monolayers, as expected for physisorbed protein films,^[^ ^27^ ^]^ which are 5–9 nm thick (Table S2, SN2, Supporting Information) and comprises 10^11^–10^12^ (molecules cm^−2^) highly packed^[^ ^28^ ^]^ recognition elements (Table S3, Supporting Information).

A physisorbed capturing layer exposed to its affinity ligand in the of 10^−7^ m range, results in a stacked “double‐layer” where no significant signal is detected below 10^−9^ m antigen concentration (Figure S5, SN3, Supporting Information). The situation changes completely when the pristine biomaterial, deposited at physiological condition (*i*_s_ = 150 × 10^−3^ m and pH = 7.4), is conditioned through exposure for 10 min to a buffer (*i*_s_ = 150 × 10^−3^ m) at a pH of either 6 (HEPES@pH6) or 8 (HEPES@pH8) (Figure S6, SN4, Supporting Information), as this simple procedure enables a single or few‐molecules to be reliably detected in 0.1 mL.

In Figure 1, the sensograms on pH‐conditioned (red curves: conditioning at pH6, blue curves: conditioning at pH8) are shown for the three biosystems. The 1 × 10^−20^ m (≈1 ± 1 analytes in 0.1 mL), addressed as “single‐molecule” and the 1 × 10^−19^ m (6 ± 2 targets in 0.1 mL) “few‐molecules” sensing regimes are investigated. Prior to these, a nonbinding interferent at much higher concentration (1 × 10^−15^ m, ≈10^5^ molecules in 0.1 mL) is assessed as well. More in details, the sensing protocol starts with the measure of the *θ*_SPR0_ baseline in the HEPES@pH7.4 buffer or in serum. Then, these are added with the nonbinding interferent and injected in the SPR cell (negative control experiment). Afterwards, two subsequent assays of “single‐molecule” and of “few‐molecules” sensing in buffer/serum solutions, are performed in the presence of the interferent. Specifically, Figure 1A shows the sensograms for the HIV‐1‐p24 protein in HEPES@pH7.4, while the *θ*_SPR_ shifts respect to the baseline (Δ*θ*_SPR_) at each sensing steps are shown in Figure 1B. For the “single‐molecule” sensing on a capturing layer conditioned at pH6, a Δ*θ*_SPR_ = (8.2 ± 2.1) × 10^−3^(°) is recorded while for the “few‐molecules” regime it reaches (14.6 ± 3.4) × 10^−3^(°). Figure 1C,D shows the same assay carried out in diluted human serum. In Figure 1A,C the average traces and their standard deviations, from three independent experiments, are shown. The steady‐state SPR signal after the removal of the target analyte is challenging to discern in these averaged traces while is more apparent in the individual experiments. For clarity, the traces in Figure 1A_1_–A_6_ display the data from each of the three independent replicated experiments at pH6 and pH8. Full details of the raw data, including the replicates originating the averages traces in Figure 1C, are provided in SN5 (Supporting Information). The assay in Figure 1C, measured directly in human serum, takes only three steps (conditioning, baseline, and sensing) lasting overall barely more than 1 h. The Δ*θ*_SPR_ shifts at each sensing steps are shown also for the NA‐b‐KRAS system in Figure 1E,F, respectively. All raw data are provided in Figures S7–S19, SN5 (Supporting Information). Similar data were also collected for the anti‐IgG system (Figure S16, Supporting Information), where, at variance, the pH conditioning is repeated before each sensing step. While this protocol results in a longer and more complicated process, no improvement is observed in the overall qualitative trend of the sensing. Hence, pH conditioning can be performed just once before the sensing starts.

In situ and operando monitoring of the temperature in the cell during the whole sensing protocol shows that it is 21.1 ± 0.1 °C (Figures S6c,d and S20, Supporting Information).

Strikingly, on all three systems of Figure 1, a signal exceeding the LOD is observed when a single‐affinity ligand binds to one recognition element among the 10^12^ present on the large (0.4 cm^2^) sensing surface even in a real fluid such as serum. This occurs on the layers conditioned at pH6, while the conditioning at pH8 provides a reliable response in the “few‐molecules” regime where higher signals are recorded. By contrast, on the nonconditioned layers, no appreciable signal is recorded until a target analyte concentration of 10^−7^ m is injected. Importantly, all the sensograms of Figure 1 exhibit a response at 10^−7^ m (Figures S7–S19, SN5, Supporting Information), thereby demonstrating that the pH‐conditioning does not impair the biolayer to functioning in the “double‐layer” regime.

A systematic comparison between the “single/few‐molecules” and the “double‐layer” regimes is provided in Figures S21–S22, SN4 (Supporting Information). An experimental design study further demonstrates that exposure of the biolayers to a sole change in ionic strength (*i*_s_) does not activate any “single/few‐molecules” regime (Figures S23–S24, Table S4, SN7, Supporting Information). The study was conducted using a 2^2^ full factorial experimental design, involving a decrease in ionic strength (∆*i*_S_) from 150 to 5 × 10^−3^ m and a decrease in pH (∆pH) from 7.4 to 6. This approach identifies the optimal condition for achieving an SPR response in the “single/few‐molecule” regime as a reduction in the pH of the rinsing buffer. As a subsequent step, the persistence of the observed effects outside this domain was evaluated by testing a shift in pH from 7.4 to 8. The purpose of this experiment was to assess whether shifting to a more basic pH could produce a comparable effect which was proven to be the case.

In addition to “single/few‐molecule” detection, the responses also exhibit very high selectivity. This is evident from the responses falling below the LOD when the sole interferents are assayed at concentrations at least 10^−4^‐fold higher than the targets. A response lower than the LOD level is also recorded when bare human serum (no addition of any nonendogenous targets), is analyzed. This occurs despite the presence in serum of proteins such as albumin, globulins, or fibrinogen at concentrations in the × 10^−3^ m range, which remain remarkably high even when the actual assayed fluid is diluted 1:20.

The refractive index of a proteins layer accounts additively for each protein amino‐acid content^[^ ^29^ ^]^ but is known to be also conditioned by changes in the surface charge density (*σ*_S_)^[^ ^30^ ^]^ or the module of the overall dipole moment per unit volume, the polarization |P|.^[^ ^29^ ^]^ They are both proportional to the surface‐potential, *Φ*_S_, and can shift due to conformational changes occurring in the detecting layer.^[^ ^31^ ^]^ Hence, our findings prove that the newly discovered “single/few‐molecules” sensing regime associated with no addition of a further layer, shifts the plasmonic resonance beyond the LOD level. Thus, demonstrating for the first time that SPR can selectively detect a single‐molecule at 10^−20^ m with a confidence level exciding 99%.^[^ ^16^ ^]^

### 2.2 “Single/Few‐Molecule" Sensing with Potentiometric Techniques: The Role of pH‐Conditioning

To corroborate the striking SPR results, the key role of the pH‐conditioning in activating the “single/few‐molecules” regime, is demonstrated, under the same experimental conditions, with several potentiometric techniques. Conformational changes associated with affinity bindings at a FET biofunctionalized gate induce a threshold voltage (*V*_T_) shift which results in a change of the source‐drain current (*I*_D_).^[^ ^18^, ^32^ ^]^ Electrolyte‐gated‐FETs, EGOFETs^[^ ^17^, ^33^ ^]^ (Figure S25a, Supporting Information), particularly well‐suited for sensing applications,^[^ ^34^, ^35^ ^]^ utilize an ionic‐conducting/electronic‐insulating electrolyte as dielectric and the gating is actuated via charge double‐layers whose capacitance, *C*_CDL_, is in the µF cm^−2^ range.^[^ ^17^, ^33^ ^]^ If, an unintentionally p‐type doped organic semiconductor (e.g., poly(3‐hexylthiophene‐2,5‐diyl, P3HT) form the FET channel, *V*_T_, the gate‐bias (*V*_GS_) needed to enter the accumulation regime, is more negative than flat‐band potential, *V*_FB_ = (*Φ*_Au _– Φ_P3HT_)/e – *σ*_S_/*C*_CDL,_^[^ ^36^ ^]^ with *Φ*_Au_ being the Au‐gate work function. *V*_T_, therefore, depends on the gate work‐function^[^ ^36^ ^]^ and on its surface‐potential, *Φ*_S_, as well as on *σ*_S_ and *P*.

For sensing purposes, the EGOFET gate‐electrode is functionalized with a biological recognition layer, e.g., anti‐HIV‐1‐p24 (Figure S25b, Supporting Information) or NA‐b‐KRAS (Figure S25c, Supporting Information). The biolayers physisorption resulted in a shift of *V*_T_ toward more negative values as compared to the bare Au‐electrode, which implies that biofunctionalized Au‐gate work‐function, *Φ*_biolayer_, is lower than *Φ*_Au._^[^ ^36^, ^37^, ^38^ ^]^

The EGOFET measuring cell, comprising also a bare Au reference gate (Figure S25d, Supporting Information), is filled with a low salinity, *i*_s_ = 5 × 10^−3^ m HEPES buffer at pH7.4 (HEPES@pH7.4/*i*_s_‐low) dielectric medium offering, at a physiological pH, a relatively large Debye Length (*λ*_D_) of 4.3 × 10^−9^ m, comparable to the recognition layers thickness (Table S2, Supporting Information). Thus, assuring adequate unscreening of the gate electrode *σ*_S_ known to resulting in higher sensitivity.^[^ ^39^ ^]^

**Figure 2:**
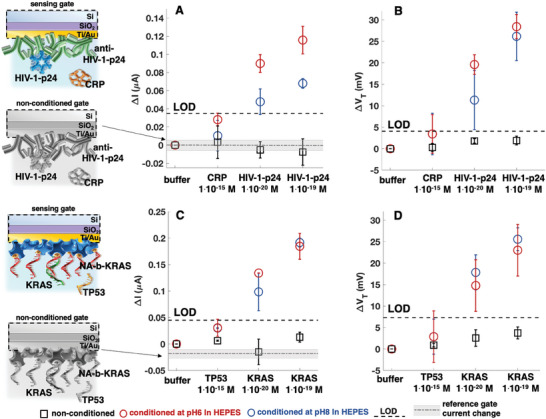
EGOFET source‐drain current (*I*_D_) and threshold voltage (*V*_T_) shifts upon “single/few‐molecules” sensing of HIV‐1‐p24 and KRAS – *I*_D_ (*V*_GS_ = −0.5 V and *V*_D_ = −0.4V) current relative shifts Δ*I* respect to *I*_0_ (*I*_0_ being the baseline measured after incubation in the bare HEPES@pH7.4) averaged over two replicates, are measured in HEPES@pH7.4/*i*_s_‐low (*i*_s_ = 5 × 10^−3^ m) after incubation of the sensing gate in the higher salinity HEPES@pH7.4 (*i*_s_ = 150 × 10^−3^ m) solutions to be assayed. The Δ*I* and Δ*V*_T_ responses of the nonconditioned biolayers (black squares), and of the biolayers conditioned at pH6 (red circles) or pH8 (blue circles), are measured with the sensing gate at each sensing step. The dashed‐dotted gray lines represent the average Δ*I*/*I*_0_ measured with the bare Au reference gate electrodes, while the shaded areas represent one standard deviation. A,B) Δ*I* and Δ*V*_T_ for the HIV‐1‐p24 “single/few‐molecules” sensing at the anti‐HIV‐1‐p24 gate, with CRP serving as negative control (raw data in Figures S27–S29, Supporting Information). C,D) Δ*I* and Δ*V*_T_ for the KRAS “single/few‐molecules” sensing at the NA‐b‐KRAS gate, with TP53 serving as negative control (raw data in Figures S29–S31,Supporting Information).

In **Figure** **2**A,B (raw data in Figures S26–S28, SN8, Supporting Information) the *I*_D_ and *V*_T_ shifts, Δ*I* and Δ*V*_T_ (vs. *I*_0_ and *V*_T0_ baselines measured in HEPES@pH7.4/i_s_‐low) for HIV‐1‐p24 sensing on differently pH‐conditioned anti‐HIV‐1‐p24 capturing layers, are shown at different steps of the “single/few‐molecules” sensing. In Figure 2C,D (raw data in Figures S29–S31, Supporting Information) homologous data are presented for the KRAS sensing. All the measurements involve the incubation of the biofunctionalized gate in the HEPES@pH7.4 buffer at physiological salinity (*i*_s_ = 150 × 10^−3^ m), added with the nonbinding species at 1 × 10^−15^ m or with a “single” (1 × 10^−20^ m) or “few” (1 × 10^−19^ m) affinity ligands. After incubation, the *I*_D_ currents are measured in HEPES@pH7.4/*i*_s_‐low. So, the nonconditioned electrodes are never exposed to a pH‐shift.

Remarkably, the gates conditioned at pH6 (red hollow circles) or at pH8 (blue hollow circles), display a reliably detectable signal beyond the LOD, already for “single‐molecule” binding. By contrast the data for the nonconditioned electrodes (black hollow squares) all fall below the LOD, clearly indicating, that the FET‐response is inhibited when no pH‐conditioning occurs. The sensing shifts *V*_T_ toward more positive values, so that after “single/few‐molecule” sensing on a pH‐conditioned biolayer, its work function, *Φ*_sensing_, increases. For instance, on the anti‐HIV‐1‐p24 system, the “single‐molecule” sensing at pH6 shows a Δ*V*_T = _(20 ± 2) mV and a Δ*I* = (0.09 ± 0.01) µA, both beyond the LOD.

A literature search (Table S5, SN8, Supporting Information) shows that several large‐area FETs capable of detecting below 10^−15^–10^−12^ m, encompass a non‐intentional pH‐conditioning of the detecting biofunctionalized electronic surface. This generally occurs during the necessary transfer of the biofunctionalized transducing interface, from the incubating solutions (at physiological pH) to the measuring electrolyte solutions with lower salinity and/or pH, in the intention of maximizing the Debye length and hence the charge unscreening and sensing response. In this study, the gate of the nonconditioned EGOFET devices is intentionally never subjected to any pH change. The responses falling below the LODs clearly demonstrate how this pH‐conditioning solely enables the ultralow LODs and not the often‐evoked amplification associated with a FET transduction. The latter, which affects both the noise and the sensing signal, has little to no effect on lowering the LOD.^[^ ^18^ ^]^ Moreover, the enabling of the “single/few‐molecule” sensing regime is not related to the current stabilization that occurs during potential cycling. The latter was demonstrated to be associated with a reduction in the dispersion of the polarization of the physisorbed layer.^[^ ^38^ ^]^

It is important to recall that, while single‐molecules have been potentiometrically detected at 10^−20^ m,^[^ ^17^ ^]^ the role of the pH‐conditioning in enabling the “single/few‐molecule” sensing regime is disclosed here for the first time.

Kelvin probe force microscopy (KPFM)^[^ ^40^ ^]^ images can quantify *Φ*_S,biolayer_ in a 90 × 90 µm^2^ area hosting ≈10^8^ capturing antibodies.^[^ ^37^ ^]^ The apparatus is schematically shown in Figures S32a, SN9, Supporting Information) along with an energy diagram for the *Φ*_biolayer_ and *Φ*_S,biolayer_ energy levels, before and after sensing, that are compared to the eV_T_ shifts in an EGOFET (Figure S32b, Supporting Information).

Atomic force microscopy (AFM) morphological images (Figure S33, Supporting Information) of the three as deposited capturing and protein–probe biolayers at the sharp interface with an exposed portion of the substrate, show that the layers exhibit a densely packed surface coverage with contiguous structures much larger than an individual capturing or protein–probe elements.

**Figure 3:**
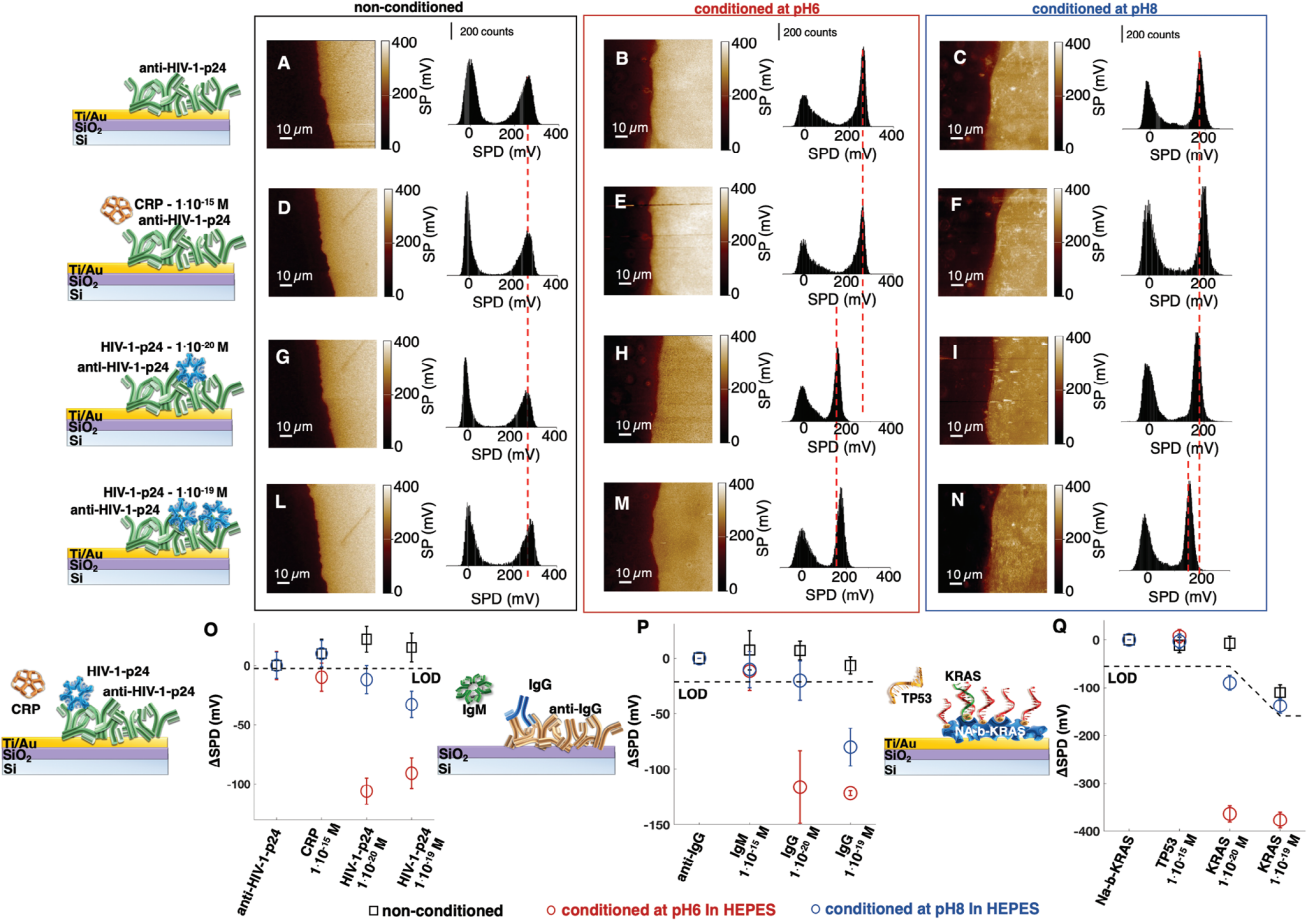
KPFM images of surface potential difference (SDP) shifts upon “single/few‐molecules” sensing of two antigens and a DNA strand. The SPDs of anti‐HIV‐1‐p24 capturing layers at different steps of the HIV‐1‐p24 sensing protocol are imaged for nonconditioned capturing biolayers ({A,D,G,L} set of panels, black frame) or on the biolayers conditioned at pH6 ({B,E,H,M}, red frame), and pH8 ({C,F,I,N}, blue frame). Images 90 × 90 µm^2^ are taken at each sensing step (first row: baseline in HEPES@pH7.4; second row: negative control experiment; third row and forth row: “single‐molecule” and “few‐molecules” sensing). The measurements are carried out in air after washing in HEPES@pH7.4/*i*_s_‐low. The samples are patterned to create a sharp interface between the biolayer and the substrate, serving as an internal standard. Each panel comprises both the image and the relevant histogram distribution. ΔSPD (SPD shifts with respect to the baseline recorded before the sensing) average values are plotted at the different stages of the sensing protocol for the anti‐HIV‐1‐p24 (O), the anti‐IgG (raw data Figure S34, Supporting Information) (P), and the NA‐b‐KRAS (raw data Figure S35, Supporting Information) (Q) biosystems, on nonconditioned (black squares), conditioned at pH6 (red circles) or pH8 (blue circles).

In **Figure** **3**A–N (Supporting Information) the KPFM images featuring the surface potential difference (SPD =$\frac{\Phi_{\mathrm{Au}}-\;\Phi_{\;\mathrm{biolayer}}}{e}\;$, SN9, Supporting Information) across the interface between the Au (taken as reference) and the biolayer covered portion of the sample, are shown along with the relevant SPD histograms. Here, the anti‐HIV‐1‐p24 capturing layers, nonconditioned or conditioned at pH6 or pH8, are inspected. After incubation, at each step of the “single/few‐molecules” sensing, the samples are washed with the low‐salinity HEPES@pH7.4/*i*_s_‐low buffer, to prevent involuntary pH‐shift, and dried. The same data are given for the anti‐IgG on SiO_2_ and NA‐b‐KARS on Au in Figures S34 and S35 (Supporting Information), respectively. In Figure 3O–Q the SPD shifts (ΔSPD) values with respect to the corresponding baseline signals, SPD_0_, recorded in the plain buffer, are summarized for all three biosystems. Figure 3B shows a KPFM SPD shift upon anti‐HIV‐1‐p24 biolayer physisorption with *Φ*_biolayer_ < *Φ*_Au_ (Figure S32b, Supporting Information) in agreement with the EGOFET data. This holds for all the three biolayers inspected (Figures S34b,S35b, Supporting Information). Like SPR and EGOFET experiments, also KPFM responses in the “single/few‐molecules” regimes are demonstrated to fall beyond the LOD only on the pH‐conditioned capturing/probe biolayers. For the HIV‐1‐p24 sensing at 10^−19^ m (Figure 3M) an ΔSPD of (−106 ± 16) mV is recorded. Moreover, also the KPFM potentiometric analyses (Figure 3O–Q, Supporting Information) show how after the sensing, *Φ*_sensing_ increases (with respect to the baseline, Figure S32b, Supporting Information) in all the three inspected biosystems.

The KPFM data provide an additional crucial insight: only on the pH‐conditioned samples, one or few binding events trigger the change in the surface potential of a large sample area hosting 10^8^ recognition elements. This means that upon pH‐conditioning, each binding event can shift the surface potential of hundred‐millions of other capturing antibodies or protein–probe complexes, thus, widely propagating the effects of the conformational change that directly affects only the antibody or protein–probe complex directly involved in the affinity binding. Moreover, the extended surface potential shift is independent of the presence of a metallic substrate as it is here demonstrated to occur also for the anti‐IgG layer deposited on Si/SiO_2_ (Figure S34, Supporting Information). Notably, none of the KPFM features here demonstrated have been related to the biolayer pH‐conditioning, before.

Another potentiometric analysis involves the measurement of the solid surface zeta (*ζ*) potential^[^ ^41^ ^]^ (Figure S36, SN10, Supporting Information). It shows for the anti‐HIV‐1‐p24 layer conditioned at pH6, an isoelectric point pI = 3.8 ± 0.1, indicating that at pH > 3.8 ± 0.1 the capturing layer, is overall negatively charged. After sensing, pI decreases significantly to 3.4 ± 0.1 while its surface *ζ*‐potential shifts by 31 ± 1 mV. Hence, also the very large surface (3.5 cm^2^) inspected by *ζ*‐potential sizably shifts, upon “few‐molecules” sensing, its *Φ*_S_ and *σ*_s_. The latter, which is located at the surface of the biolayer (SN10, Supporting Information), becomes less negative upon sensing. An electrochemical impedance spectroscopy analysis (Figure S37, SN11, Supporting Information) shows that the capacitance of the biofunctionalized gate increases after sensing by Δ*C* = 0.35 ± 0.07 µF.

A summary of the pieces of evidence gathered so far is given in Table S6a, SN12 (Supporting Information) while a diagram of *Φ*, *Φ*_S_, and *V*_T_ shifts is presented in Figure S32b (Supporting Information). The anti‐HIV‐1‐p24 system conditioned at pH6 is taken as an archetypal example, but the conclusions apply to all the three biosystems investigated. At pH = 7.4 the pH‐conditioned biolayers surface hold a negative *σ*_s‐biolayer_ (pI = 3.8 ± 0.1, Figure S36e, Supporting Information) which is lower than *σ*_s‐Au_, (pI < 2, Figure S36c, Supporting Information) with an associated polarization *P*_biolayer_ lower than *P*_Au_, both oriented in the same direction. This is compatible with a measured *Φ*_S,biolayer_ < *Φ*_S,Au_, meaning that during physisorption the capturing antibodies or protein–probe complexes dipole moments, *μ*, are statistically oriented with their positive pole pointing away from the surface. Hence, the protein and protein–probe dipoles partially compensate for the negative charge present on the bare Au‐surface. Upon “single/few‐molecule” sensing an electrostatic rearrangement withing the biolayer (pI = 3.4 ± 0.1, Figure S36f, Supporting Information) leads to *σ*_s‐sensing_ > *σ*_s‐biolayer_, *Φ*_S,sensing_ > *Φ*_S,biolayer_ and *P*_sensing_ > *P*_biolayer_. Hence, upon “single/few‐molecule” sensing, the biolayer polarization increases but the surface is still negatively charged as *P*_Au_ > *P*_sensing_. Importantly, none of these changes are associated with any mass being added to the biolayer surface. Even if a few target molecules are irreversibly attached to the biolayer, encompassing trillions of recognition elements, the mass change would be negligible. Instead, the potentiometric shifts are to be associated with a rearrangement of the tertiary structures of the recognition elements.

The overall Δ*σ*_s_ shift upon “few‐molecules” sensing can also be estimated (Table S6b, Supporting Information): a Δ*q* = Δ*ζ *× Δ*C* = 10.85 ± 2.55 × 10^−9 ^C is computed, assuming comparable Δ*C* shifts for anti‐HIV‐1‐p24 and anti‐IgG, which is reasonable as quantitatively analogous ΔSPD increases (Figure 3O,P) are measured on both biolayers. Taking *σ*_s = _Δ*q*/*A* (*A* = 3.5 cm^2^) and 1.3 × 10^12^ cm^−2^ for a physisorbed anti‐HIV‐1‐p24 (Table S3, Supporting Information), about 1.5 *e* equivalent charges are shifted each 10^2^ capturing antibodies, upon “single/few‐molecules” sensing.

### 2.3 Role of pH‐Conditioning in Self‐Propagating Proteins Aggregation

An amplification mechanism must be in place to explain the measured charge displacement occurring upon “single/few‐molecule” sensing at a millimeter‐wide interface. Without such an amplification, it would be impossible for a single molecule, whose footprint is on the order of 10^−10^ mm^2^, to generate any measurable signal on an interface that is up to 10¹¹ times larger, as the signal would otherwise fall into the noise. In this section, the role of pH‐conditioning of the antibodies or protein–probe recognition biolayer in enabling a novel and remarkable amplification effect is unveiled. Before delving into this aspect, it is important to recall that, unlike nanometric detecting interfaces where a diffusion barrier is present, a single or few molecules in 0.1 mL can effectively reach a seemingly large detecting interface. This finding is supported both experimentally and through dedicated modeling based on the Einstein equation for Brownian motion.^[^ ^42^ ^]^

**Figure 4:**
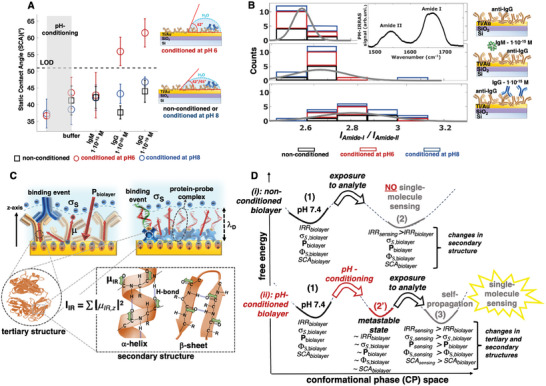
Hydrophobic interactions and self‐propagating aggregation. A) Average static contact angles (SCA, three replicates) measured at each sensing step on nonconditioned (black squares), conditioned at pH6 (red circles), or conditioned at pH8 (blue circles) anti‐IgG biolayers. The first data points (from the left) are relevant to the pristine as deposited anti‐IgG layers that undergo a pH‐conditioning or not. Afterward, the samples undergo the standard sensing steps (SN13, Supporting Information). B) Distribution of the Amide I and Amide II peak integrated areas ratios, IRR = *I*_Amide‐I_/*I*_Amide‐II_, measured on 54 samples (Table S7, SN15, Supporting Information). A typical PM‐IRRAS spectrum in the 1480–1750 cm^−1^ amide region is shown in the inset. In the top row, baseline data taken from the anti‐IgG layers are displayed, in the middle row the spectra are taken from the anti‐IgG exposed to IgM 1 × 10^−15^ m (negative control experiment), while the bottom row represents the IgG sensing at 1 × 10^−15^ m. Black, red, and blue boxes in the histograms indicate the samples conditioned according to the color code. The gray line is the gaussian fit of the data. C) Schematic representation of the structure of a capturing layer sensing its antigen (top left) and of a protein–probe complex sensing its complementary DNA target (top right). The insets at the bottom feature the tertiary and the secondary molecular structure of a generic protein. D) Energy landscapes of the conformational‐phase (CP) space in the phenomenological model of the “single/few‐molecules” at a large interface. The subscript “biolayer” indicates the Au surface modified by the deposited biolayer.

To gather more evidence in support of the amplification mechanism, another aspect investigated is the degree of hydrophilicity of a 1 cm^2^ capturing layer surface, influencing the static contact angle (SCA) measured after pH‐conditioning and at each sensing step (**Figure** **4**A) with the setup described in SN13, Figures S38 and S39 (Supporting Information). Following the sensing protocol, a non pH‐conditioned biolayer is assessed as well showing a SCA_0_ of 43 ± 7 (°). Once more the key role of the pH‐conditioning in enabling sensing at the physical limit, is proven with SCA reaching 61 ± 4 (°) in the few‐molecule sensing regime on a biolayer conditioned at pH6. This is an increase in SCA of 18 ± 7 (°) as compared to the baseline SCA_0_. No appreciable signal is recorded after conditioning at pH8 in this experiment. The bare‐eyes visible increase in the hydrophobic character of the biolayer surface after “single/few‐molecule” sensing, is complemented by a shift in the nanomechanical properties of capturing layer conditioned at a pH of ≈5.5 (Figure S40, SN14, Supporting Information). Upon binding of few antigens (antigens/capturing antibodies ratio of 10^6^), a shift in both Young's modulus, decreasing by (37 ± 8)%, and of the adhesion force distributions, increasing by (66 ± 12)%, are measured. This is typical of hydrophobic surfaces that are known to be stickier,^[^ ^43^, ^44^ ^]^ more flexible and less stiff^[^ ^45^ ^]^ as the Young's modulus decreases.^[^ ^46^ ^]^

Another piece of relevant information toward the identification of a phenomenological sensing mechanism, is provided by a polarization modulation infrared reflection‐absorption spectroscopy (PM‐IRRAS)^[^ ^47^ ^]^ investigation. A schematic view of the apparatus is shown in Figure S41, SN15 (Supporting Information). Here, the transition dipole moments μ_IR_, whose squared *z*‐component additively contributing to the PM‐IRRAS signal *I*_IR_ =∑|μ_IR,z_|^2^ , is measured. μ_IR_ relates to the backbone of the polypeptides chains, hence to the protein secondary structure. In Figure 4B, the distributions of peak area ratios, addressed as infra‐red‐ratios (IRRs) = *I*_Amide‐I_ */I*_Amide‐II_ at the different sensing steps, are shown. Relevantly, IRR is proven to quantitatively increases with a protein α‐helix content.^[^ ^48^ ^]^ The baseline samples have an IRR = 2.59 ± 0.04 that slightly increases in the negative control experiments (IRR = 2.67 ± 0.11) and reaches the value IRR = 2.81 ± 0.15 upon affinity binding. Hence a slight increase in the α‐helices content is seen while no appreciable change in the H‐bonded β‐sheet component appears to be in place. In agreement with the literature,^[^ ^49^ ^]^ no sizable dependance of IRR on pH‐conditioning is seen, either. The reliability of these results is tested with an artificial intelligence based data analysis (Figures S42 and S43, SN15, Supporting Information).

The biological recognition layer is schematically depicted in Figure 4C featuring an Au gate electrode covered by a monolayer of capturing antibodies (on the left) and of protein–probe complexes (on the right) along with their dipole moments *μ*. The affinity binding antigens and DNA stands are shown as well. The overall net dipole moment per unit volume of the Au covered by the biolayer *P*_biolayer_, is oriented with its positive pole pointing to Au; this biofunctionalized surface has a negative *σ*_s_ (Figure S32b, Supporting Information). It is to be recalled that, while *P*, *σ*_s_, *Φ*_S_, and SCA shifts reveal changes in proteins tertiary structure, PM‐IRRAS relates to changes in their secondary structure.

Protein folding and unfolding are governed by the complex and irregular nature of rugged energy landscapes, which describe the relationship between a protein free energy and its conformational phase (CP) space. This CP space encompasses all possible structural configurations that a protein can adopt as it transitions from its completely unfolded state to its native configuration. The native configuration is the optimal folding ensuring full functionality, such as the ability to selectively bind a cognate affinity partner. A protein folding process occurs spontaneously driven by various molecular interactions and the energy landscapes of the CP spaces are characterized by numerous local minima and maxima. Local minima represent relatively stable conformations, such as tertiary folded states or intermediate structures along the folding pathway. Transition states, which are higher energy configurations, must be traversed during processes such as folding, unfolding, or structural transitions between different conformations. Proteins navigate these energy landscapes through diverse pathways, often exploring multiple intermediate states before achieving their final folded or functional forms.

It is conceivable that a physisorbed layer of antibodies or protein–probe complexes has its own CP space. Figure 4D illustrates a hypothetical representation of the CP space for a generic biolayer crowded with protein‐based recognition elements. Trace (i) depicts a CP space with two relative minima corresponding to the free energy state **(1)** of a pristine, as‐deposited, non‐pH‐conditioned physisorbed biolayer and the lower energy state **(2)** reached by this biolayer after interacting with a single, or a few, affinity cognate molecules. In this case, no detectable signal is observed for “single/few‐molecule” binding events. In contrast, trace (ii) illustrates, after state **(1)**, the energy state **(2’)** assumed by the biolayer after undergoing pH‐conditioning. In this case, the biolayer reaches an even lower energy state **(3)** after a single or a few affinity binding events occur. In this case, thanks to the pH‐conditioning, a detectable signal is observed upon “single/few‐molecule” binding.

Functional proteins in their native conformation can unfold either when they are deposited on a substrate^[^ ^50^ ^]^ or when exposed to a low‐pH environment.^[^ ^23^ ^]^ Misfolding can lead to the exposure of the hydrophobic amino acids to the solvent, and this triggers the formation of ordered amyloid‐like or amorphous aggregates^[^ ^23^ ^]^ which are both known to be very stable. It is assessed that, conditioning at an acidic pH involves partially unfolded proteins in self‐propagating aggregation pathways^[^ ^46^ ^]^ that proceed via nonspecific interactions between polypeptide sequences.^[^ ^23^ ^]^ The driving force toward self‐assembly of unfolded protein films can be very strong and once the growth starts it is characterized by a very steep kinetic process.^[^ ^23^ ^]^ The amorphous aggregates are much less studied, but evidence for fast self‐propagation proceeding via a glass‐like phase transition is reported.^[^ ^51^ ^]^ In immunoglobulins aggregation at pH6, a metastable intermediate is observed that is characterized by little changes in the secondary structure, but significant tertiary structure variations.^[^ ^52^ ^]^ Moreover, it is shown that, during the first 15 min amyloids‐β aggregations are largely amorphous, characterized by α‐helix or random‐coil, rather than β‐sheet, structures.^[^ ^53^ ^]^

Based on the state‐of‐the‐art knowledge reviewed in the previous paragraph (involving almost only proteins in solution) and the data gathered in this study, a phenomenological “single/few‐molecules” sensing mechanism is proposed, whose key aspects are here described:

- Nonconditioned Biolayer **–** The CP state **(1)** of an as deposited biolayer is characterized by a specific set of properties as highlighted in Figure 4D: *σ*_s‐biolayer_, *Φ*_S,biolayer_, *P*_biolayer_, SCA_biolayer_, and IRR_biolayer_. This film, being physisorbed is known to consist of a single monolayer of partially misfolded or unfolded proteins.^[^ ^38^, ^54^ ^]^ As anticipated, protein misfolding often results in the exposure of hydrophobic amino acid residues to the surrounding solvent or to adjacent proteins within the biolayer. Under normal conditions, these hydrophobic regions are typically buried within the protein interior, shielding them from the aqueous environment to maintain structural stability and functionality. However, their exposure facilitates interactions through hydrophobic forces, which can lead to the aggregation of proteins into insoluble globular structures or fibrils.^[^ ^55^ ^]^ AFM investigation (Figure S33, Supporting Information) of the biolayers studied here, reveal the absence of fibril‐like domains. Instead, the biolayers exhibit globular features, suggesting a distinct aggregation behavior that is likely influenced by the specific conditions and interactions within the system. When “single/few‐molecule” sensing is performed in the absence of a pH‐conditioning as in state **(2)**, only a slight increase in IRR (IRR_sensing_ > IRR_biolayer_) is observed, indicating minor yet non‐negligible modifications in the proteins secondary structure. These changes are associated with a slight increase in the formation of α‐helix structures. On the other hand, the absence of significant changes in *σ*_s‐biolayer_, *Φ*_S,biolayer_, *P*_biolayer_, SCA_biolayer_ parameters, confirms that the tertiary structure of the biolayer proteins, remains unaffected.
- pH‐conditioned biolayer – The pH‐conditioning of a physisorbed layer in state **(1)** can generate a further misfolding of the capturing antibodies or of the protein–probes complexes This state is identified as CP **(2’)** in Figure 4D. Complete protein misfolding is typically associated with a loss of binding affinity, as the structural integrity of the protein is fully compromised, diminishing its ability to effectively interact with target molecules. However, in the present study, pH conditioning of the physisorbed layer concur to induce only partial misfolding, as evidenced by the pH‐conditioned biolayers ability to retain binding affinity at concentrations up to 10^−7^ m. At this concentration, a “double‐layer” is formed, consisting of the originally present biolayer of capturing antibodies (protein–probe complexes) to whom an equally large number of antigens (DNA stands), bind. Notably, no differences are observed in the number of antigens (DNA strands) attached to the capturing (protein–probe complexes) layer when comparing pH‐conditioned biolayers to non‐pH‐conditioned ones (SN3¸ Supporting Information). All the investigated properties of state (**2’),** *σ*_s‐biolayer_, *Φ*_S,biolayer_, *P*_biolayer_, IRR_biolayer_ and SCA_biolayer_, are practically unchanged as compared to those measured for the system in state **(1)** so, pH‐conditioning alone comes with little to no secondary or tertiary structural changes. This is a strong support to attributing the features that are formed, to amorphous aggregates as the formation of more ordered β‐amyloids would show a sizable increase in β‐sheet PM‐IRRAS signals. The measured IRRs signal show, instead, only weak evidence of changes in the α‐helix structure in the film. Moreover β‐amyloids fibrils are characterized by a Young's modulus of few GPa,^[^ ^23^ ^]^ while here we measure a parameter three orders of magnitude lower (Figure S39c, Supporting Information). Hence, we can summarize that, upon pH‐conditioning, amorphous aggregates form, but these are characterized by no significant detectable change in any of the physical parameters measured (apart from IRR). On the other hand, pH‐conditioning is also known to create metastable protein species in solution.^[^ ^56^, ^57^ ^]^ Extending this effect to the biolayers studied here, we can assume that state **(2’)** acts as a “silent” metastable state, enabling the transition to a different state that would not otherwise be accessible. Upon “single/few‐molecules” affinity binding the biolayer converts to CP state **(3)**, where a significant change in the tertiary structure occurs as all the related physical quantities, *Φ*_s_, *σ*_s_, *P*, *ζ*‐potential and SCA, sizably shift. All the biolayer show, in fact, an increase in *Φ*_s_, (*Φ*_S,sensing_ > *Φ*_S,biolayer_) and in the surface density of negative charges *σ*_s_ (*σ*_s‐sensing_ > *σ*_s‐biolayer_) which is associated with an increase of *P* (*P*_sensing_ > *P*_biolayer_) along with a dramatic increase in hydrophobicity, as shown by the extremely large SCA measured (SCA_sensing_ > SCA_biolayer_). A very prominent reduction of the Young's modulus is seen as well. Hence after binding the biolayer turns highly hydrophobic and due to an aggregation process^[^ ^23^ ^]^ that is fueled through very effective and rather strong short‐range hydrophobic interactions.^[^ ^24^ ^]^ The resulting aggregates may act as nucleation sites, further accelerating the clustering of additional hydrophobic residues, thereby amplifying the overall effect. Indeed, short‐range hydrophobic interactions are a key driving force in the propagation of aggregation, as they facilitate the clustering of hydrophobic regions, creating a cascade effect that accelerates the assembly of already aggregated structures.^[^ ^58^ ^]^ Indeed, the single‐binding event, involving just one recognition elements, is proven by KPFM to propagate to at least 10^8^ capturing/probe elements that are crowded on the large detecting interface. These are general features that affect both proteins and protein–probes complexes in line with the hydrophobic forces driven self‐propagating aggregations, being nonaminoacidic specific.^[^ ^23^ ^]^ Such a very fast aggregation process is at the basis of a self‐propagating mechanism that likely involves an affinity‐binding triggered transition^[^ ^51^ ^]^ occurring in each of the biolayers studied that acts as an amplification effect of the “single/few‐affinity” binding manifesting through a very large change in the proteins tertiary structure. It is the first time that: i) such a self‐propagating aggregation is seen on a biolayer that undergoes a pH‐conditioning (so far it was proven in solution); ii) hydrophobic aggregation is proven to be triggered also by an alkaline pH‐conditioning; iii) the self‐propagating effect is proven to be triggered also by an affinity binding.

## 3 Conclusions and Future Perspectives

This work demonstrates that plasmonic single‐molecule immunometric and molecular detections can be achieved with a LOD of 10^−20^ m also in human serum in just over an hour. Potentiometric and surface sensitive techniques data support an amplification effect that involves the self‐propagation of an amorphous aggregation of partially misfolded proteins, induced by pH‐conditioning (both mildly acidic or alkaline) and affinity binding. The resulting electrostatic rearrangement in the biomaterial involves a displacement of about 1.5 *e* equivalent charges each 10^2^ recognition elements.

This discovery has profound implications for understanding the rich and yet unclarified landscape of metastable states characteristic of physisorbed protein materials and opens new opportunities for highly reliable advanced biomaterials in applications such as plasmonic biosensing at the physical limit. While the “Single‐Molecule with a large Transistor – SiMoT”^[^ ^17^, ^35^ ^]^ is a qualitative electronic point‐of‐care (PoC) platform that it is now at the pre‐clinical stage (https://www.singlemolecule.center/en/home‐english/), the present study opens to the “Single‐Molecule with a Large Surface – SiMoLS” technology where a single‐molecule plasmonic platform can be developed for PoC early detections. Indeed, miniaturized SPR systems are very compact handheld devices^[^ ^59^, ^60^ ^]^ that hold significant potential for real‐time, label‐free diagnostics, by detecting protein biomarkers in blood or saliva. This is based on a novel biomaterial that can change the approach to immune and molecular sensing. Their ability to provide rapid, sensitive, and accurate results in conjunction with single‐molecule LODs, make them ideal for early disease detection and personalized medicine.^[^ ^61^ ^]^ Moreover, the portability and robustness of miniaturized SPR devices, coupled with their ability to analyze complex biological matrices such as blood or saliva without extensive sample preparation, position them as ideal candidates for PoC applications. Their capacity to deliver real‐time, label‐free detection of biomarkers with single‐molecule sensitivity is particularly advantageous for early disease diagnostics, even in resource‐limited or decentralized healthcare settings. Additionally, their compatibility with multiplexed analyses and integration into microfluidic platforms enhances their utility, enabling precise and efficient monitoring of multiple biomarkers critical for personalized medicine and timely clinical interventions.

## 4 Experimental Section

### Materials

Anti‐HIV‐1‐p24, produced in mice, is a monoclonal antibody targeting the recombinant HIV‐1‐p24 capsid protein (molecular weight M_W_ = 26 kDa), the latter produced by *Escherichia coli*, and both are purchased from Abcam (Cambridge, UK). Bovine serum albumin (BSA, M_W_ = 66 kDa) and Human C‐reactive protein (CRP) (M_W_ = 115 kDa), isolated from human fluids (ascitic/pleural), were acquired from Sigma–Aldrich. The anti‐human Immunoglobulin G (anti‐IgG) is a Fc specific polyclonal antibody produced in a goat (M_W_ = 144 kDa), while the human IgG (M_W_ = 150 kDa) affinity ligand and the human IgM (M_W_ = 950 kDa) nonbinding ligand, were extracted from human serum and are both purchased from Sigma–Aldrich. A biotinylated KRAS^G12V^ (b‐KRAS, 5’Biotin‐TGCCTACGCCAACAGCTCCAACTAC, Invitrogen, MW: 7.924 Da) serves as probe while attached to a NeutrAvidin protein (NAV, Thermo Scientific™, cod:31000, MW: 60 kDa). The KRAS^G12V^ rev is the target analyte (GTAGTTGGAGCTGTTGGCGTAGGCA, Invitrogen, MW: 7810 Da) while the TP53 (GGCGGCATGAACTGGAGGCCCATCC, Invitrogen, MW: 7694 Da) is the nonbinding target in negative control experiments. HEPES, M_W_ = 238.30 g mol^−1^) dry powder, sodium chloride (NaCl, M_W_ = 58.44 g mol^−1^, BioXtra, ≥ 99.5%, AT) and sodium hydroxide (NaOH, M_W_ = 40 g mol^−1^, reagent grade, ≥ 98%, pellets, anhydrous) were purchased from Merck. Phosphate buffer saline (PBS) tablets and poly(3‐hexylthiophene‐2,5‐diyl), P3HT (regioregularity >99%, average MW = 20 000–45 000 g × mol^−1^), were purchased from Sigma–Aldrich. Deionized water is HPLC grade (resistivity of 1 × 10^6^ Ohm). Human blood serum was produced from a healthy donor aged between 18 and 60, screened and tested negative for HIV and hepatitis B and C that is purchased from Research Donors Ltd. (London, UK). All the reagents were used as received with no further purification or processing. Pipettes were supplied by Gilson Pipetman L P100L 10–100ul Metal Ejector, cod: FA10004M. The surface plasmon resonance (SPR) gold coated optical glasses were purchased from BioNavis Ltd.

### Buffer Solutions

The HEPES buffers in water (HPLC grade) were prepared from an HEPES 100 × 10^−3^ m stock solution diluted down to 5 × 10^−3^ m. HEPES@pH7.4 (pH7.4, ionic strength *i_s = _* 150 × 10^−3^ m), was prepared by adding a 110 µL aliquot of NaOH 5 m to 250 mL of HEPES 5 × 10^−3^ m. The ionic strength was adjusted by adding 2.12 g of NaCl dry powder. The same procedure was used for the HEPES@pH6 (pH6, *i*s*_ = _* 150 × 10^−3^ m) buffer prepared by adding 8 µL of NaOH 5 m to 250 mL of HEPES 5 × 10^−3^ m. Then an aliquot of 2.12 g of NaCl was added to adjust the ionic strength. HEPES@pH8 (pH 8, *i*_s_ *_= _* 150 × 10^−3^ m, pH = 8) was prepared by adding 150 µL of NaOH 5 m and 2.12 g of NaCl to 250 mL of HEPES 5 × 10^−3^ m. A low salinity (*i*_s_ *_= _* 5 × 10^−3^–10 × 10^−3^ m) HEPES@pH7.4 buffer addressed as HEPES@pH7.4/i*_s_*‐low, was also used. The pH was controlled by a HANNA‐HI5221 advanced research grade benchtop pH/mV meter. The total ionic strength of buffer solutions was calculated according to the $i_{s}=1/2\sum \left(C_{i}\times {z_{i}}^{2}\right)$ equation, where *C*_i_ is the concentration of the i^th^ ionic species and *z*_i_ is its charge. A PBS buffer solution at ionic strength of 162 × 10^−3^ m and pH7.4, mimicking the physiological condition of human plasma, was also used. The pooled human serum was prepared by centrifugation at 10^4^ g for 5 min to remove insoluble material and it was then diluted 1:20 (v/v) with HEPES@pH7.4.

### “Single/Few‐Molecules” Standard Solutions

Standard solutions of IgG, IgM, CRP, HIV‐1‐p24 antigens as well as KRAS^G12V^ (here simply KRAS) and TP53 nucleic acids in HEPES@pH7.4 were prepared by serial dilution from mother solutions (50 × 10^−6^ m). The dilution was performed according to the equation *c*_2 =_ *V*_1_/*V*_2 _× *c*_1_ = *k* × *c*_1_, where *c*_1_ and *c*_2_ are the initial and diluted ligand concentrations, respectively, *V*_1_ and *V*_2_ are the corresponding solutions volumes, while *k* = *V*_1_/*V*_2_, is the dilution factor. The stock solution was used for subsequent dilutions in a ten‐fold serial dilution protocol. Given the factorial nature of the dilution equation, the absolute uncertainty (both random and systematic errors) on the concentration (from the mother solution down to 10^−20^ m) is the error on the sampled volume, certified to be 1% by the pipette supplier company. At very low concentrations, the Poisson sampling error must be considered.^[^ ^17^ ^]^ Indeed, at this extremely low concentration Poisson sampling limits the probability of finding 1 or 2 analytes to 64%. Accordingly, not every measurement at this concentration will produce a signal, as sometimes no target molecules are sampled in 0.1 mL.^[^ ^62^ ^]^ The Poisson error was quantified by the square root of the nominal particle count which exceeds 1% when there are less than 10^4^ particles in 0.1 mL (2 × 10^−17^ m in 0.1 mL). The number of molecules is # = *c* (concentration) × *N*_a_ (Avogadro's number) × *V* so in 0.1 mL of a 1 × 10^−20^ M or a 1 × 10^−19^ M solution 0.6 ± 0.8 (approximated to nearest whole values to be 1 ± 1) or 6 ± 2 molecules are found, respectively. The same procedure was used to prepare the standard solutions in PBS. For the preparation of the biomarker standard solutions in real fluid, the diluted human serum samples were added with the HIV‐1‐p24 capsid protein.

### Substrates Metallization and Cleaning

The following substrates are used: – i) SPR slides: optical glasses (BK7) coated with a Cr(2 nm)/Au(50 nm) bilayer; ii) Si/SiO_2_: Si(n‐type)/SiO_2_(300 nm) atomically flat wafers; iii) Si/SiO_2_/Au: Si/SiO_2_ wafers coated with a Ti(5 nm)/Au(50 nm) bilayer. Before the metallization, the substrates were treated in a Piranha solution (3:1 mixture of sulfuric acid and hydrogen peroxide) and cleaned by ultrasonic bath in acetone and 2‐propanol for 10 min. Afterward, an e‐beam evaporation of the Ti adhesion promoter and of the Au layer was carried out. The substrates were then treated for 10 min in a UV‐ozone cleaner before proceeding with the biofunctionalization. The sample for the KPFM investigation skips this last step. The nonmetallized substrates were rinsed in an ultrasonic bath with acetone, 2‐propanol in deionized water for 10 min, and then dried in a nitrogen flux. For the SPR measurements, the commercial slide was cleaned in an NH_4_OH/H_2_O_2_ aqueous solution (1:1:5 v/v) at 80–90 °C for 10 min. It was then rinsed in deionized water, dried in a nitrogen flux, treated for 10 min in a UV‐ozone cleaner. If not otherwise stated, the substrates/samples area was 0.4–0.5 cm^2^.

### Biofunctionalization

The protocol involves the physisorption of the biological recognition elements, the capturing antibodies, or the protein–probe complexes on the different substrates. For the anti‐HIV‐1‐p24 and anti‐IgG capturing antibodies, the substrate was incubated in 0.1 mL of the HEPES@pH7.4 buffer solution of the capturing antibodies (50 µg mL^−1^) for 2 h and the substrate was then rinsed with HEPES@pH7.4 to remove any unbonded protein. The biofunctionalization with the DNA probes is carried out starting with the physisorption of neutravidin (NA) from an NA 50 µg/mL HEPES@pH7.4 buffer (0.1 mL) for 2 h, followed by rinsing in HEPES@pH7.4. Then the sample was further incubated for 1 h in a 3.96 µg/mL b‐KRAS HEPES@pH7.4 buffer solution (0.1 mL). A washing step in HEPES@pH7.4 was then performed. The biorecognition elements physisorption was monitored in situ by manually injecting the recognition element solution into the SPR cell. All the biofunctionalizations were carried out at 21.0 °C. (Figures S2 and S3, SN1, Supporting Information).

### Drying Protocol

Some of the experiments were carried out in air, which required the samples to be dried. To minimize the salt precipitation on the surface, the samples were thoroughly washed in the low salinity (*i*_s = _5–10 × 10^−3^ m) HEPES@pH7.4 solution, addressed as HEPES@pH7.4/i*_s_*‐low. The subsequent drying step was carried out by spin‐coating at 1–3 × 10^3^ rpm in air for 1 min.

### pH‐Conditioning Protocol

The pH‐conditioning protocol entails incubating the as‐deposited recognition biolayer into 0.1 mL of an HEPES@pH6 or HEPES@pH8 buffer solution for 30 min, followed by thorough rinsing in HEPES@pH7.4 for about 10 min, to restore physiological conditions and to retain only the pH induced irreversible change (conditioning) (Figure S6, SN4, Supporting Information). The characterization of the recognition biolayer before and after the pH‐conditioning protocol was carried out by measuring the PM‐IRRAS spectra (see SN15, Supporting Information). The data show that the PM‐IRRAS spectra are invariant, thus demonstrating that the protonation/deprotonation equilibrium is not affected by the pH‐conditioning protocol designed as the biolayer is brought back to equilibrium at physiological conditions by exposure to the HEPES@pH7.4 buffer.

### Sensing Protocol

The sensing protocol involves the following steps, sequentially performed on the same sample: (i) baseline – the biofunctionalized sample is incubated for 10 min in 0.1 mL of an HEPES@pH7.4 solution; (ii) negative control experiment – the biofunctionalized sample is further incubated for 10 min in 0.1 mL of a 1 × 10^−15^M HEPES@pH7.4 solution of a nonbinding species, namely: IgM (on anti‐IgG), CRP (on anti‐HIV‐1‐p24), or TP53 (on NA‐b‐KRAS); (iii) “single‐molecule” sensing regime – the same sample is incubated for 10 min in 0.1 mL of 1 × 10^−20^M (≈1 ± 1 molecules) HEPES@pH7.4 solutions (including the interferents) of the affinity binding species, namely: IgG on anti‐IgG and HIV‐1‐p24 on anti‐HIV‐1‐p24 or KRAS on NA‐b‐KRAS; (iv) “few‐molecules” sensing regime – the same sample is incubated for 10 min in 0.1 mL of 1 × 10^−19^ M (6 ± 2 molecules) HEPES@pH7.4 solutions of the affinity binding species. (v) “double‐layer” sensing regime – the same sample is incubated for 10 min in 0.1 mL of a 1 × 10^−7^ m (≈10^13^ molecules) HEPES@pH7.4 solutions of the affinity binding species, including also the interferents. Sensing experiments are performed also detecting single and few HIV‐1‐p24 antigens in diluted human serum. Moreover, the sensing protocol is always carried out not only on the pH‐conditioned detecting biolayers but also on a pristine nonconditioned one. In the latter cases no “single/few‐molecule” sensing signal is recorded (SN1–SN5, Supporting Information).

### Incubation Times, Washing Time, and Volumes Used

pH conditioning, represented by a gray‐shadowing in the figures, is conducted for 30 min; incubation in 0.1 mL of the solution under assay lasts for 10 min and is denoted in the figures by a black solid arrow; rinsing or washing, marked by a black dashed arrow in the figures, is performed in a 3 mL HEPES@pH7.4 or HEPES@pH7.4/*i*_s_‐low solution for 10 min.

### Reliability Statistical Indicators

The LOD level, represented by a black dotted line, is calculated as the noise average value plus three times its standard deviation.^[^ ^16^ ^]^ This is a parameter assuring that the data falling higher than the LOD are true values (not spurious fluctuation of the noise) with a confidence level better that 99%. The noise level is taken as the data dispersion in the negative control experiments. Random errors, indicated in the figures by shadowing or a by a bar, are taken as one standard deviation over *N* = 2–6 replicates.

### Surface Plasmon Resonance

SPR data were collected using a BioNavis‐200 Multi‐parameter Surface plasmon resonance (MP‐SPR) Navi instrument in the Kretschmann configuration^[^ ^26^, ^63^ ^]^ (Figure S1, SN1, Supporting Information). The SPR slide, docked in a 0.1 mL flow‐through cell, consisted of a gold coated optical glass matching the high refractive index of the prism on which the laser beam strikes. The solutions were injected manually in batches of 0.1 mL. The SPR apparatus (vide infra) was equipped with two laser beams that simultaneously inspect two different points (3 mm apart) of the sensing interface, to assess the uniformity of the film over the 0.4 cm^2^ area inspected. The two traces measured concur to the number of replicates acquired for a given experiment. The laser incident angle *θ* was varied in the 50.290°–77.930° range with an instrumental error, or Δ*θ*_noise_, of 1 × 10^−3^ (°) corresponding to one unit shift in the SPR reflectance intensity,^[^ ^64^ ^]^ while a *θ* shift, Δ*θ*, of 86.3 (°) corresponds to one refractive index unit (RIU). The major specifications of the SPR setup are the following: sensitivity *S*_θ _= Δ*θ*/RIU = 86.3 (°)/RIU while the detection accuracy, taken as Δ*θ*_noise_/*S*_θ_, is 1.15 × 10^−5^ RIU. Typical Δ*θ*_RI‐0.25_ (plasmonic peak width at 0.25 reflectance intensity^[^ ^65^ ^]^) of 2.6 ± 0.4(°) and *θ*_0_ (plasmonic peak angle) of 69.9 ± 0.2(°) were measured (see Figures S2–S4, Supporting Information), hence a quality factor of 27 ± 4 can be computed. The figure of merit is *S*_θ_/Δ*θ*_RI‐0.25_ hence equal to 33 ± 5 RIU^−1^. All the experiments were performed through an in situ and operando control of the temperature that was oscillating in the 21.1 ± 0.1 °C range as shown in Figures S6c,d and S20 (Supporting Information). The measured sensograms were the plasmon resonant angle *θ*_SPR_ vs. time. The measurements were preformed while a 0.1 mL batch of the solution to be assayed was injected in the cell and remained stationary in the cell during the measurement of the single/few‐molecules sensing SPR transient signal. The baseline was recorded before the sensing, after incubating the layer on the SPR slide in the HEPES@pH7.4 solution that contains no binding species. The corresponding value is *θ*_SPR_ *_0_*. The *θ*_SPR_ values were recorded during the negative control or the single/few‐molecules sensing steps, after incubating the gate in the HEPES@pH7.4 solutions of the nonbinding or of the affinity binding species. For each sensing experiment, the response was given as the angle shift, *Δθ*_SPR_ = *(θ*_SPR_ – *θ*_SPR_ *_0_)*. The SPR data treatment involved a light third‐degree smoothing routine^[^ ^66^, ^67^ ^]^ of the whole curve in all the regions except those where the injection of a new batch of solution is performed. Since such a manually performed injection involves washing out all the fluid previously present in the cell, it causes a significant hydrodynamic turbulence with large changes in the refractive index. This noise, not related with the sensing experiments, is treated with a one‐degree smoothing routine.^[^ ^66^, ^67^ ^]^ For the sake of clarity both the row data and the smoothed curves are provided as supporting figures for all the experiments (SN5 and SN6, Supporting Information).

### Electrolyte Gated Organic Field‐Effect Transistors

The EGOFETs sensing devices are fabricated, as detailed elsewhere,^[^ ^17^, ^68^, ^69^ ^]^ starting from a Si/SiO_2_ substrate patterned with photolithographically defined source (S) and drain (D) interdigitated electrodes with a channel length (spacing between S – D fingers) *L* = 5 µm and a channel width (total lengths of all the fingers) *W* = 10.500 µm. A solution of P3HT (4 mg mL^−1^), in 1,2dichlorobenzene, is spin‐coated (2 × 10^3^ rpm for 20 s) on the interdigitated electrodes area and is annealed at 90 °C for 5 min. A polyurethane well is glued around the channel area and filled with the gating medium, namely 0.1 mL of HEPES@pH7.4/*i*_s_‐low. This electrolyte (*i*_s = _5–10 × 10^−3^ m) assures a Debye Length of 96–136 nm, *λ*_D = _(*ε*_r _× KT/e^2^ × *i*_s_)^1/2^ with the dielectric constant of H_2_O taken as *ε*_r_ = 78, *e* being the elementary charge and *KT* the thermal energy). Two circular electrodes (0.2 cm^2^) serving as reference and sensing gates (G) are fabricated as Ti/Au‐coating on Si/SiO_2_ substrates. The gate serving as sensing electrode is biofunctionalized with the capturing antibodies or with the protein–probe complexes. The reference gate is not biofunctionalized and allows to assess the current flowing in the FET channel at every stage of the sensing protocol to monitor the FET device stability. The source‐drain current is $I_{D}=\frac{W\;\times \;\mu_{\mathrm{FET}}\;\times \;C_{i}}{2L}\;{\left(V_{\mathrm{GS}}-V_{T}\right)}^{2}\;\mathrm{at}\;|V_{D}\left|>\right|V_{D}^{\mathrm{sat}}|$, where *C*_i_ is the gating system capacitance per unit area, *V*_T_ is the EGOFET threshold voltage, and *µ*_FET_ is the field‐effect mobility.^[^ ^19^, ^70^ ^]^ *I*_D_ is stabilized by measuring the transfer characteristics (*I*_D_ vs. *V*_GS_ ranging from 0 to −0.5 V at a fixed *V*_D_ of −0.4 V) modulated by the reference gate, every half an hour, until the *I*_D_ transient current drift measured at *V*_GS = _−0.5 and *V*_D = _−0.4 V, reduces to 1% per hour. The current point value at *V*_GS = _−0.5 V and *V*_D = _−0.4 V falls, typically, in the region of maximum transconductance of a P3HT EGOFET. The sensing follows the general protocol with the biofunctionalized gate that is incubated in an HEPES@pH7.4 solution of the target molecule. After the incubation the gate is washed in HEPES@pH7.4/*i*_s_‐low buffer and allocated in the measuring well filled with 1 mL of the same HEPES@pH7.4/*i*_s_‐low solution. Here, a set of 20 consecutive transfer characteristics (cycling) is measured and the point value current at *V*_GS = _−0.5 V and *V*_D = _−0.4 V from the last transfer characteristic, is taken as the signal level, *I*_D_, for the relevant step. The relevant threshold voltage shift is *V*_T_. The baseline, *I*_0_, is recorded before the sensing, after incubating the gate in the HEPES@pH7.4 solution that contains no binding species. The corresponding threshold voltage shift is *V*_T0_. The *I*_D_ values are recorded during the negative control or the single/few‐molecules sensing steps, after incubating the gate in the HEPES@pH7.4 solutions of the nonbinding or of the affinity binding species. For each sensing experiment, the response is given as the current variations, *ΔI = (I*_D_ **–* I*_0_) or as *ΔV*_T_ = *V*_T_ – *V*_T0_. The stability of the EGOFET channel is independently evaluated by driving the P3HT EGOFET with the reference gate and measuring I_D_, before starting and after completion of each sensing experiment. Also in this case, sets of 20 transfer characteristics are recorded. Before and after each sensing experiment, the relative current shifts are measured with the bare gold reference gate driving the same P3HT channel. Overall, the sensing experiments performed, the reference electrode shifts fall in the very narrow range of (−0.64 ± 0.79)% (average value plus one standard deviation). The extraction of *V*_T_ from the transfer characteristics is accomplished by plotting $\sqrt{I_{D}}$ vs. *V*_G_ and evaluating *V*_T_ as the intercept to the *V*_G_ abscissa^[^ ^71^, ^72^ ^]^ (SN8, Supporting Information).

### Atomic Force Microscopy (AFM) and Kelvin Probe Force Microscopy (KPFM)

The specimens for AFM and KPFM inspection are prepared either on the Si/SiO_2_ or on the Si/SiO_2_/Au substrates. All the measurements were carried out on dried samples. The recognition elements physisorption was carried out by covering part of the substrate area with a polymeric mask.^[^ ^37^ ^]^ A physisorbed layer either of capturing anti‐HIV‐1‐p24 (on Si/SiO_2_/Au) or of anti‐IgG (on Si/SiO_2_) or of the protein–probe complexes NA‐b‐KRAS (on Si/SiO_2_/Au), was formed on the mask‐free portion of the sample surface. The AFM and KPFM measurements were performed using a NT‐MDT system NTEGRA Spectra (Moscow, Russia). The morphology images were recorded in semi‐contact mode, using a silicon probe with a resonance frequency of 318 kHz, apex radius of 7 nm, spring constant of 44 N m^−1^, and a quality factor *Q* = 404 (Bruker, mod. TESPA‐V2). KPFM data were acquired with a platinum‐iridium coated tip (TipsNano, mod. FMG01/Pt) with apex size of 35 nm, resonance frequency *f* = 79 kHz, spring constant 3 N m^−1^ and quality factor *Q* = 240. The KPFM images were obtained in the two‐pass mode, involving two sequential scans per line. During the first scan, the sample surface morphology was acquired in semi‐contact mode. During the second scan, the piezo shaker remained inactive, and the tip retraced the sample profile while it was raised by LH = 250 nm and a DC + AC electrical signal was applied. The DC voltage required to nullify the electrostatic force on the tip at the AC frequency, corresponds to the tip‐sample SDP. To measure the SPD between the biofunctionalized and the pristine substrate, each KPFM image was acquired by scanning 90 × 90 µm^2^ areas across the interface between the biofunctionalized and pristine substrate zones. Following this method, the SPD remains very stable even in the presence of surface contamination from adventitious contaminants.^[^ ^37^ ^]^ Care was taken to scan the same part of the sample at each step of the sensing protocol. All images were processed with the Image Analysis software. The reliability of the comparison of the KPFM measurements carried out in dried condition and the EGOFETs was addressed in detail elsewhere.^[^ ^38^ ^]^ All the measurements were performed in air at room temperature (22 °C) (SN9, Supporting Information).

### Solid Surface Zeta Potential of the Physisorbed Capturing Layer

The zeta (*ζ*)‐potential of a capturing layer physisorbed onto a gold‐coated glass surface (17.5 cm^2^) was measured using a SurPASS 3 streaming potential technique. To this end, the current generated when an electrolyte solution flows through an adjustable gap system was measured. A polyvinylidene difluoride (PVDF) reference surface was also incorporated in the cell. During measurement, the antibody‐coated glass was secured in the cell with a gap of approximately 100 µm set between it and the PVDF reference surface. A buffered KCl electrolyte solution with a concentration of 10 × 10^−3^ m and pH 5.5 was used to facilitate ionic conduction during the measurements. The movement of the electrolyte was induced by pressurizing the liquid reservoir and both the pressure variance across the measuring cell (Δ*p*) and the streaming current (*I*_str_) are monitored (SN10, Supporting Information).

### Static Contact Angle

SCA was measured by dispensing a 2 µL droplet of deionized water on a dried sample surface, a 1 cm^2^ large Si/SiO_2_/Au substrates biofunctionalized with an anti‐IgG capturing layer. The static contact angle was registered at each sensing step with a RameHart 100 goniometer and the droplet on the surface was photographed with a GoPro Macro camera. All experiments were carried out in duplicate on two different samples and three different areas were inspected for each sample (SN13, Supporting Information).

### Polarization Modulation Infrared Reflection‐Absorption Spectroscopy

PM‐IRRAS spectra were recorded using a Nicolet iS50 Fourier transform infrared (FT‐IR) spectrometer optically coupled with an external module which includes a wire‐grid linear polarizer, a photoelastic modulator (PEM), a sample holder, and a liquid nitrogen‐cooled mercury‐cadmium‐telluride (MCT) detector. The incident beam hits the sample at a grazing angle of 82° to the normal of the sample surface. The PEM modulates the polarized incident radiation swinging the electric field from a direction parallel to the surface (s‐polarization) to a direction normal to the surface (p‐polarization) with a frequency of 100 kHz. The PEM phase settings are chosen to maximize the PM‐IRRAS response at 1450 cm^−1^ and ensure the highest signal‐to‐noise ratio in the spectral region of interest (1.000–1.800 cm^−1^). Both the spectrometer and the PM‐IRRAS module were constantly purged to keep the relative humidity below 16%, and at a negligible CO_2_ level. The sample compartment temperature was kept constant in the 22–24 °C range. Each spectrum was averaged over 1.000 interferograms acquired with 4 cm^−1^ resolution. The baseline correction on the original PM‐IRRAS signal was calculated in the 950–1.780 cm^−1^ range using a least‐square third‐order polynomial fitting method.^[^ ^73^ ^]^ The reflective metallic substrates were the Ti/Au‐coated glass slide. Each substrate was sequentially cleaned with ultrasonic baths for 10 min in water, acetone, and 2‐propanol, followed by drying under nitrogen flux and ozone cleaning for 10 min. Then, 1.5 mL of 50 mg mL^−1^ anti‐IgG in HEPES@pH7.4 was drop casted on the sample and left to rest for 2 h. Three sets of samples were investigated. For the baseline acquisition, the biofunctionalized substrates were rinsed in HEPES@pH7.4/*i*_s_‐low buffer, dried by spinning in air for 180 s at 1500 rpm, and measured. For the negative control measurements, the biofunctionalized substrates were rinsed in HEPES@pH7.4 buffer, incubated with a 1 × 10^−15^ m IgM solution in HEPES@pH7.4 buffer for 40 min, rinsed in HEPES@pH7.4/*i*_s_‐low buffer, dried, and measured. For the sensing measurements, the biofunctionalized substrates were rinsed in HEPES@pH7.4 buffer, incubated with a 1 × 10^−15^ m IgG solution in HEPES@pH7.4 buffer for 40 min, rinsed in HEPES@pH7.4/*i*_s_‐low buffer, dried, and measured. The effect of a pH shift was investigated conditioning the samples in HEPES@pH6 or HEPES@pH8 buffers (SN15, Supporting Information).

## Conflict of Interest

The authors declare no conflict of interest.

## Author Contributions

E.M. and C.D.F. contributed equally to this work. L.T. and G.S. conceived the project. The experiments were designed by E.M., C.D.F., G.S., and L.T. SPR, EGOFETs, and SCA experiments were performed by C.S., L.S., M.P., M.C., M.C., and P.B., while C.D.F. and M.P. performed AFM, KPFM, and PM‐IRRAS experiments. The data were analyzed by M.C. and E.M. The paper was written by L.T. and revised by E.M., C.D.F., and G.S. with contributions from all other authors.

## Supporting information



Supporting Information

## Data Availability

All data needed to evaluate the conclusions of this study are available in this Article and its Supporting Information. datasets generated and analyzed during the current study are available in the ReCaS (https://recascloud.ba.infn.it) repository, University of Bari.
