# Supplementary material for: Plasmonic Single‐Molecule Affinity Detection at 10−20 Molar
Source: Adv Mater. 2025 Jan 23;37(9):2418610. doi: 10.1002/adma.202418610 (PMC11881672; doi:10.1002/adma.202418610)
Supplement: Supplementary file 1 — Supporting Information [file ADMA-37-2418610-s001.docx]

Supporting Information for

Plasmonic single-molecule affinity detections at 10^-20^ molar

Eleonora Macchia^1,2,3^†, Cinzia Di Franco^4,2^†, Cecilia Scandurra^5,2^, Lucia Sarcina^5,2^ Matteo Piscitelli^6^, Michele Catacchio^1,2^, Mariapia Caputo^1,2^, Paolo Bollella^5,2^, Gaetano Scamarcio^7,6,^*, and Luisa Torsi^5,2^*

^1^Dipartimento di Farmacia-Scienze del Farmaco, Università degli Studi di Bari Aldo Moro, 70125 Bari, Italy; ^2^Centre for Colloid and Surface Science, Dipartimento di Chimica, Università degli Studi di Bari Aldo Moro, 70125 Bari, Italy; ^3^ Faculty of Science and Engineering, Åbo Akademi University, Turku, Finland; ^4^CNR IFN, 70126 Bari, Italy, ^5^Dipartimento di Chimica, Università degli Studi di Bari Aldo Moro, 70125 Bari, Italy; ^6^Dipartimento Interateneo di Fisica, Università degli Studi di Bari Aldo Moro, 70125 Bari, ^7^NEST, Istituto Nanoscienze - CNR and Scuola Normale Superiore, Pisa, I-56127 Italy.

†Equally contributing first authors

*Corresponding authors Email: luisa.torsi@uniba.it, [gaetano.scamarcio@uniba.it](mailto:gaetano.scamarcio@uniba.it)

This file includes:

Supporting Notes: **SN1** - **SN15**

Supporting Figures: **Figure S1** - **S44**

Supporting Equations: **Equation S1** - **S15**

Supporting Tables: **Table S1** - **S7**

Supporting References: **1 - 105**

| Supporting Notes (SNs) Table of Contents | | **pag.** |
| --- | --- | --- |
|  |  |  |
| ***SN1. Surface plasmon resonance (SPR) basics***  *Apparatus and monitoring of recognition elements physisorption*  *Overview of the state of the art in SPR immunosensing* | | **3** |
| *figs. S1 - S4, tab. S1* | |  |
| ***SN2. Physisorption: estimate of surface coverage and density*** | | **7** |
| *tab. S2* | |  |
| ***SN3. The SPR antigen sensing in the double-layer regime*** | | **8** |
| *fig. S5* | |  |
| ***SN4. The pH conditioning of the capturing biolayers*** | | **9** |
| *fig. S6* | |  |
| ***SN5. SPR raw data of the single/few-molecules sensing*** | | **10** |
| *The anti-HIV-1-p24 capturing biolayer sensing in HEPES@pH7.4*  *The anti-HIV-1-p24 capturing biolayer sensing in human serum*  *The anti-IgG capturing biolayer exposed to multiple pH shifts*  *The NA-b-KRAS probe biolayer*  *Surface coverage of the biological layer*  *figs. S7 – S20, tab. S3* | |  |
| ***SN6. Comparison between the single/few-molecules and the double-layer regimes*** | | **25** |
| *fig. S21* | |  |
| ***SN7. An ionic strength change does not enable single molecule detections*** | | **27** |
| *figs. S22 - S24, tab. S4* | |  |
| ***SN8.* EGOFETs single/few-molecules sensing** | | **33** |
| *Bioelectronic sensing of the HIV-1-p24*  *Bioelectronic sensing of KRAS*  *A pH shift can explain also literature papers on ultra-sensitive large-area FET biosensors*  *figs. S25 – S31, tab. S5* | |  |
| ***SN9. AFM and KPFM surface potential investigation*** | | **42** |
| *KPFM and EGOFET work function (Φ) and surface potential energy (Φs) diagrams*  *AFM of the biolayer*  *KPFM inspection of the non-conditioned and pH conditioned anti-IgG biolayer on SiO_2_*  *KPFM insp. of the non-conditioned and pH conditioned NA-b-KARAS biolayer on Au*  *figs. S32 - S35* | |  |
| ***SN10. Solid Surface Zeta Potential of the physisorbed capturing layer*** | | **47** |
| *fig. S36* | |  |
| ***SN11. Electrochemical impedance spectroscopy analysis*** | | **49** |
| *fig. S37* | |  |
| ***SN12. Plasmonic and potentiometric data on “single/few-molecules” detection*** | | **49** |
| *tab. S6* | |  |
| ***SN13. SCA measurements on pH-conditioned capturing layer*** | | **51** |
| *figs. S38, S39* | |  |
| ***SN14. Nanomechanical characterization of a conditioned anti-IgG layer*** | | **53** |
| *fig. S40* | |  |
| ***SN15. Spectroscopic characterization with PM-IRRAS*** | | **54** |
| *The PM-IRRAS principle*  *The PM-IRRAS multivariate data processing*  *Assessment of the capturing layer protonation/deprot. before and after pH-conditioning*  *figs. S41 - S44, tab. S7* | |  |
| ***Supporting References*** | | **60** |

**Supporting Notes**

***SN1. Surface plasmon resonance (SPR) basics***

*Apparatus and monitoring of recognition elements physisorption*

In **Figure S1a** and **Figure S1b** the schematics of the physisorbed anti-HIV-1-p24 and anti-IgG capturing layers are shown along with the HIV-1-p24 and IgG affinity ligands as well as of the CRP and the IgM non-binding interferents. In **Figure S1c** the NA-b-KRAS protein-probe complexes layer is featured along with its complementary KRAS target and the TP53 interferent both being mutated genes serving as markers for pancreatic cancer precursors^[1]^. The protein-probe complexes layer is composed of the NA protein and the b-KRAS probe.

Surface plasmon resonance (SPR) in the Kretschmann-Raether configuration, **Figure S1d**, is a surface-sensitive spectroscopic technique that enables the assessment the refractive index changes of an ultra-thin biological layer deposited on the approximately half a centimeter squared SPR slide allotted in the 0.1 mL flow-through cell^[2–8]^.


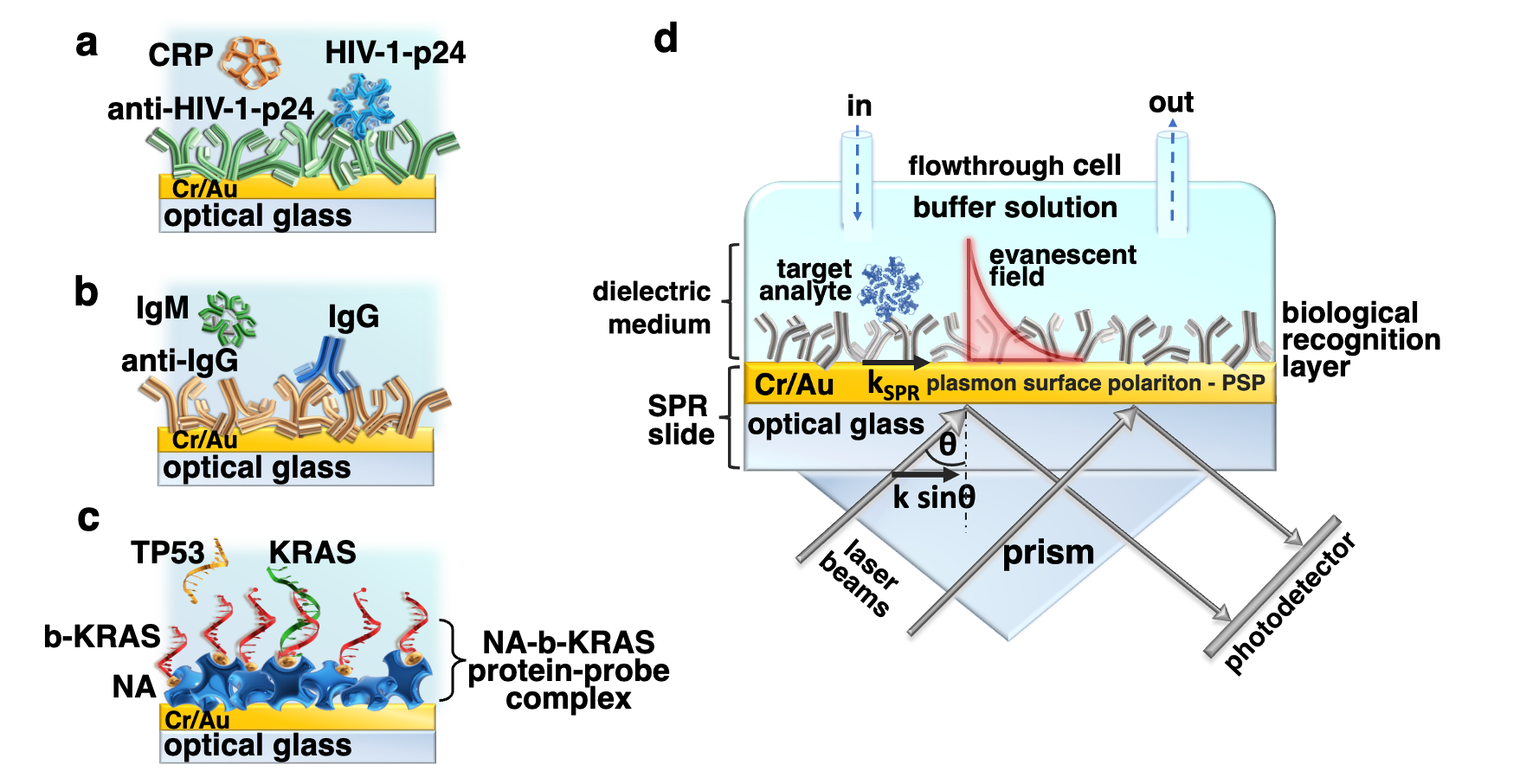


**Figure S1**. **SPR apparatus -** (**a**) the HIV-1-p24 antigen captured by the anti-HIV-1-p24 physisorbed layer in the presence of the CRP interferent; (**b**) the anti-IgG capturing layer is shown along with the IgG affinity ligand and the IgM interferent; (**c**) KRAS target DNA probed by the b-KRAS complementary probe in the presence of the TP53 interferent strand. The b-KRAS is attached to the surface *via* biotin-neutravidin binding to a physisorbed NA layer (NA-b-KRAS). (**d**) Schematic representation of the SPR apparatus in the Kretschmann configuration. laser is split into two beams that can simultaneously inspect different portion of the exposed biolayer large area located 3 mm apart. Schematic representation of the systems studied, namely:

The principle underpinning the SPR technique can be summarized as follows: *i)* a laser beam (λ = 670 nm) strikes the glass/metal interface and couples to the plasmon surface polariton (PSP) states propagating along the metal/biolayer/electrolyte interface. *ii)* PSP field amplitude decays exponentially outside the metal layer as an evanescent wave propagating for δ ≈ 100 - 300 nm in a direction perpendicular to the interface, where it can interact with the biolayer film. *iii)* The plasmon resonance (full coupling between the incident light energy and PSP) occurs when the PSP wave-vector, is equal in module and direction, to the *k⋅sinθ*_SPR_ component of the incident light wave-vector *k* in the plasmonic propagation direction. This occurs at a given incident resonant angle *θ*_SPR_ and at the plasmon resonance **Eq. S1** holds:

$\boldsymbol{k}_{\boldsymbol{0}}\boldsymbol{\cdot}\boldsymbol{n}\boldsymbol{\cdot}\boldsymbol{sin}\boldsymbol{\theta}_{\boldsymbol{SPR}}\boldsymbol{=}\boldsymbol{k}_{\boldsymbol{0}}\boldsymbol{\cdot}\sqrt{\frac{\boldsymbol{\varepsilon}_{\boldsymbol{metal}}{\boldsymbol{\cdot}\boldsymbol{n}}_{\boldsymbol{d}}^{\boldsymbol{2}}}{\boldsymbol{(}\boldsymbol{\varepsilon}_{\boldsymbol{metal}}\boldsymbol{+}\boldsymbol{n}_{\boldsymbol{d}}^{\boldsymbol{2}}\boldsymbol{)}}}$ (S1)

where *k_0_* is the incident light wave-vector in vacuum, *ε_metal_* is the real part of the plasmon dielectric constant in the metal and *n_d_* is the refractive index of the dielectric medium.^[9]^ In the present case the metallic coating is a Cr/Au interface characterized by the *ε_Cr_* and *ε_Au_* constants, while the dielectric medium comprises the gold/biolayer (characterized by an effective refractive index *n*) and the HEPES buffer or diluted human plasma solutions. The *θ*_SPR_ angle of the reflectivity dip is seen, in **Figure S2a**-**S4a** during the deposition of the capturing or protein-probe complexes layers. The SPR traces that measure, *in-situ* and *operando*, the transient angle-shift, Δ*θ*_SPR_, are referred to as sensograms**.** The angular shift is evaluated with respect to a baseline signal, for instance the *θ*_SPR_ of the plane gold slide in a biolayer deposition experiment.

Typical sensograms for the physisorption of anti-HIV-1-p24, anti-IgG and NA-b-KRAS capturing or probe layers, are shown in **Figure S2b**, **Figure S3b** and **Figure S4b**, respectively.


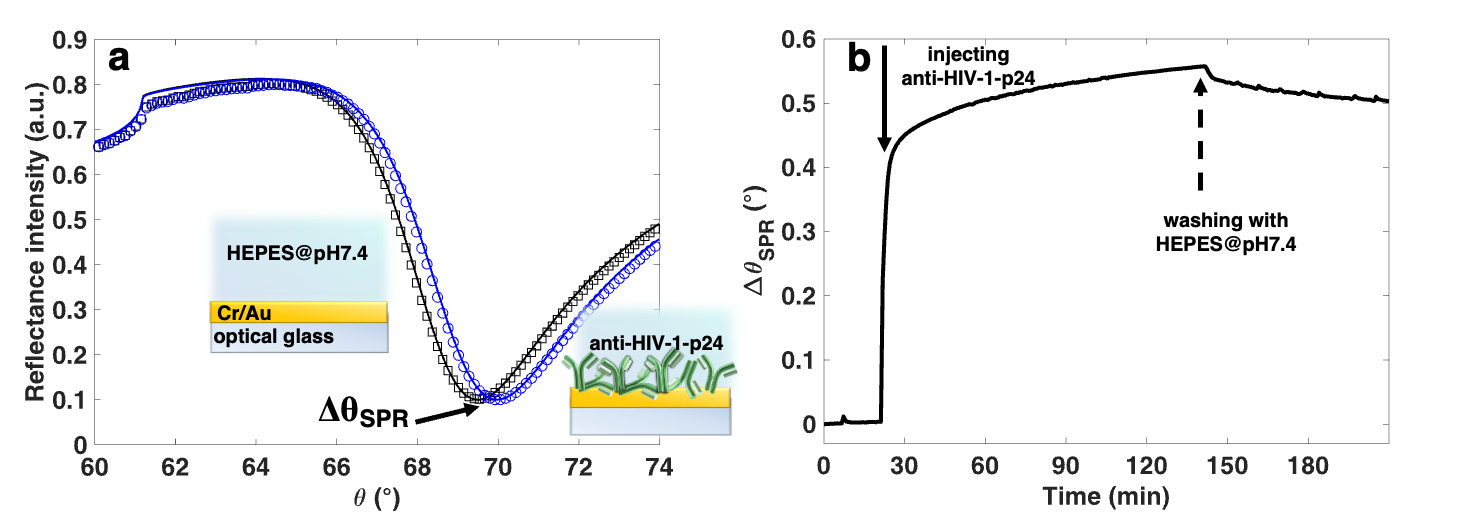


**Figure S2. SPR sensogram of the anti-HIV-1-p24 layer physisorption**: **(a)** Reflectance intensity *vs.* incident laser beam angle θ before and after the physisorption of the anti-HIV-1-p24 capturing layer from a HEPES@pH7.4 buffer solution of the capturing antibodies; the plasmonic resonance dip for the bare SPR slide (hollow black squares) and for the slide covered by the physisorbed anti-HIV-1-p24 layer (hollow blue circles) are shown. **(b)** Sensogram of the SPR angle shift (Δθ_SPR_) during the whole physisorption process.

**
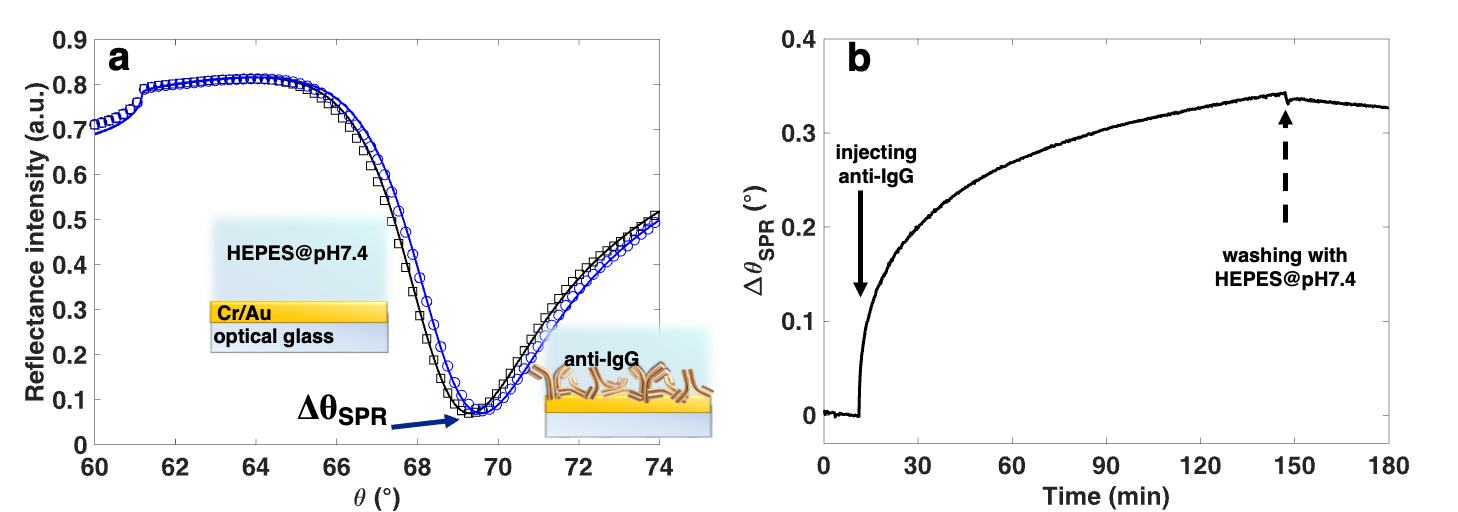
**

**Figure S3. SPR sensogram of the anti-IgG layer physisorption** **- (a)** Reflectance intensity *vs.* incident laser beam angle θ before and after the physisorption of the anti-IgG capturing layer from a HEPES@pH7.4 buffer solution of the capturing antibodies; the plasmonic resonance dip for the bare SPR slide (hollow black squares) and for the slide covered by the physisorbed anti-IgG layer (hollow blue circles) are shown. **(b)** Sensogram of the SPR angle shift (Δθ_SPR_) during the whole physisorption process.

**
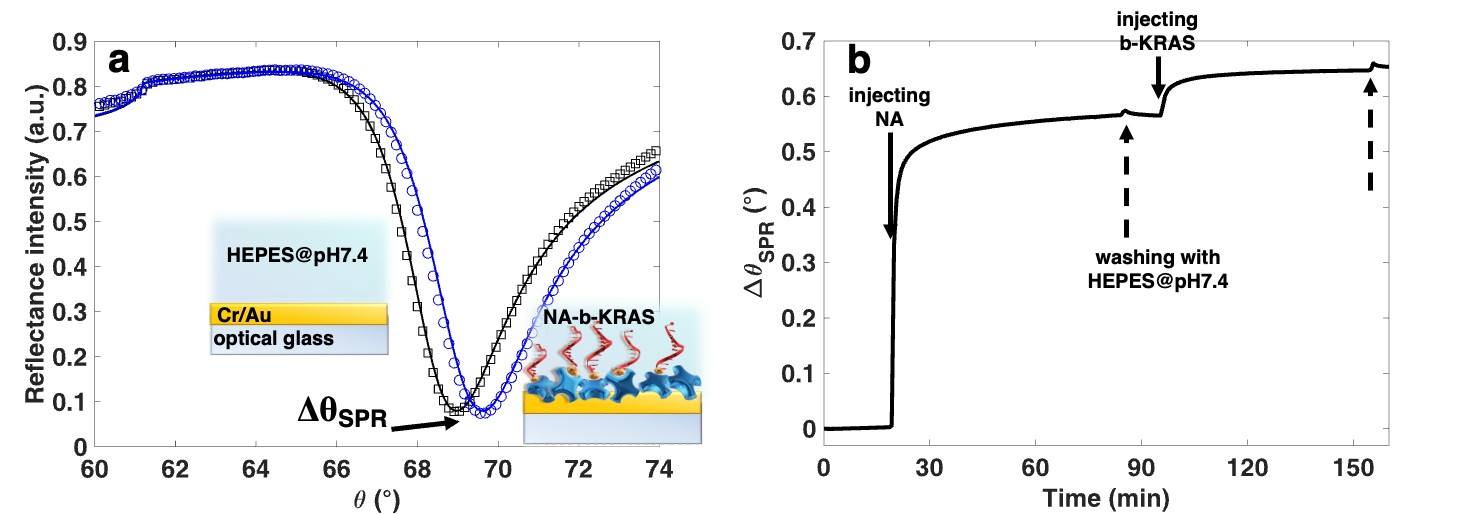
**

**Figure S4. SPR sensogram of the NA-b-KRAS layer physisorption** **- (a)** Reflectance intensity *vs.* incident laser beam angle θ before and after the physisorption of the Na-b-KRAS layer from a HEPES@pH7.4 buffer solution of the probe; the plasmonic resonance dip for the bare SPR slide (hollow black squares) and for the slide covered by the physisorbed NA-b-KRAS layer (hollow blue circles) are shown. **(b)** Sensogram of the SPR angle shift (Δθ_SPR_) during the whole physisorption process.

*Overview of the state of the art in SPR immunosensing*

In **Table S1** an overview of the Limit of Detections (LODs)^[10]^ obtained with various SPR approaches is provided, showing how the standard SPR LODs typically fall within the 10^-9^ M range. However, they can reach 10^-13^-10^-12^ M when plasmon-enhanced effects are employed. In the table a selection of different representative papers is included.

**Table S1:** Overview of the state of the art in SPR immunosensing

| **technique** | **detection range /LOD** | **fabrication/features** | **references** |
| --- | --- | --- | --- |
| Surface Plasmon Resonance (SPR) | 0.5·10^-9^M – 1.2·10^-6^M  LOD: 0.5·10^-9^M | Lysozyme detection in PBSA with gold-based SPR interface functionalized with anti-lysozyme DNA aptamer. | ^[11]^ |
| Surface Plasmon Resonance (SPR) | 8·10^-12^M – 4·10^-9^M  LOD: 8.5·10^-12^ M | Detection of total Prostate-Specific Antigen (tPSA) in human serum, with a sandwich assay using antibody-modified nanoparticles. | ^[12]^ |
| Surface Plasmon Resonance (SPR) | 0.5·10^-9^M – 1·10^-6^M  LOD: 4·10^-9^ M | Human immunodeficiency virus (HIV-1) p24 protein detection with covalently immobilized anti-p24 in PBS. | ^[13]^ |
| Surface Plasmon Resonance (SPR) | 1·10^-9^M – 0.6·10^-6^M  LOD: 2·10^-9^ M | SARS-CoV-2-Spike1 (S1) protein detection using SPR direct assay encompassing physisorbed capture antibodies in PBS. | ^[14]^ |
| Fiber-Optic SPR (FO-SPR) | (1.5 – 30) ·10^-9^ M  LOD: 1.5·10^-9^ M | Signal amplification through gold nano-particles (Au NPs) functionalized with a goat anti-mouse antibody (GAM) of anti-Progesterone (P4). Assay performed in Milk samples diluted in PBS. | ^[15]^ |
| Fiber-Optic SPR (FO-SPR) | (2 – 270) ·10^-9^ M  LOD: 2·10^-9^ M | IgG direct immunoassay with covalently immobilized anti-human IgG in PBS. | ^[16]^ |
| Multiplexing Surface Plasmon Resonance (SPR) | kappa: (2 – 500) ·10^-9^M  LOD: 2·10^-9^M  lambda: 4·10^-9^M - 1·10^-6^M  LOD: 4·10^-9^M  B2M: (3 – 185) ·10^-9^M  LOD: 3·10^-9^M  HSA: (6 – 700) ·10^-9^M  LOD: 6·10^-9^M | Multiplexed detection of Urine proteins: Kappa light chain protein (kappa), Lambda light chain protein (lambda), Beta-2-microglobulin (B2M), Human Serum Albumin (HSA) via multi-channel SPR in diluted urine samples. | ^[17]^ |
| Evanescent wave absorbance (EWA) based U-bent fiber optic sensors | 2·10^-12^ M – 700·10^-9^M  LOD: 2·10^-12^M | Human IgG detection with Gold-nanoparticle (Au-NP) labels in PBS. | ^[18]^ |
| Fiber-Optic SPR (FO-SPR) | 7·10^-12^M - 0.7·10^-9^M  LODs: 9.4·10^-12^M (serum),  7·10^-12^M (plasma),  9·10^-12^M (whole blood) | Sandwich immunoassay of a therapeutic drug antibody, infliximab (IFX), spiked in diluted serum, plasma and whole blood. | ^[19]^ |
| Multi-Walled Carbon Nanotubes (MWCNTs) enhanced SPR | 125·10^-12^M - 250·10^-9^M  LOD: 125·10^-12^M | Detection of Tau protein in a sandwich immunoassay format using capture antibodies against Tau-protein labeled with MWCNTs. | ^[20]^ |
| Indirect inhibition SPR immunoassay | 1.4·10^-12^M – 76·10^-12^M  LOD: 2·10^-12^M | Detection of female 17-β estradiol hormones in buffer through covalent immobilization of estradiol-BSA conjugate. | ^[21]^ |
| Localized Surface Plasmon Resonance (LSPR) | 200·10^-15^M - 2·10^-12^M  LOD: 1·10^-12^M | Tumor Necrosis Factor alpha (TNF-alpha) protein detection with a sandwich LSPR immunoassay in diluted human serum (1:1). | ^[22]^ |
| Localized Surface Plasmon Resonance (LSPR) | 1·10^-15^M - 70·10^-9^M  LOD: 1·10^-15^M | Bacterial cholera toxin (CT) detection through AuNPs-enhanced SPR combined with atom transfer radical polymerization (ATRP). | ^[23]^ |
| Localized Surface Plasmon Resonance (LSPR) | 300·10^-15^M - 3·10^-9^M  LOD: 300·10^-15^M | Detection of prostate-specific-antigen (PSA) using AuNPs-antibody conjugate and a sandwich assay format. | ^[24]^ |
| Localized Surface Plasmon Resonance (LSPR) | lysozyme: (2- 29) ·10^-6^M  LOD: *n.a.*  lactoferrin: (0.4 – 5) ·10^-6^M  LOD: *n.a.* | Composite detecting interfaces (AuNS/PNM hydrogel) to detect lysozyme and lactoferrin in diluted human tears. | ^[25]^ |
| Surface Plasmon Resonance microscopy (SPRM) | 25·10^-15^M - 1·10^-9^M  LOD: *n.a.* | Sandwich immunoassay of troponin T (TnT) in human serum. | ^[26]^ |

***SN2. Physisorption: estimate of surface coverage and density***

The refractive indexes and the thicknesses of all the layers are modelled with Fresnel equations (Winspall 3.02)^[27]^. In **Table S2** the relevant data, including the thicknesses and the refractive indices of the anti-HIV-1-p24 and anti-IgG capturing layers, are given.

**Table S2: Outputs of the Winspall 3.02 modelling** of the data presented in **Figures S2a,** **S3a** and **S4a** providing estimates of the refractive indices, n, or of the real part of the dielectric constant, ε, of the SPR slide (glass and plating layers) and of the dielectric media encompassing the physisorbed anti-HIV-1 p24, anti-IgG and NA-b-KRAS biolayer

| layer | thickness (10^-9^m) | real part of the dielectric constant (*n* or *ε*) | imaginary part of the dielectric constant |
| --- | --- | --- | --- |
| **glass BK7** | *n.a.* | n = 1.518* | 0 |
| **Cr/Au** | 2±1 / 40±1 | ε_Cr_ = 3.3±0.2 / ε_Au_ = 0.16±0.02 | 2.8±0.4 / 3.85±0.05 |
| **anti-HIV-1-p24 in HEPES@pH7.4** | d = 9±1 / *n.a.** | n = 1.38±0.01 / n_s_ = 1.33** | 0 / 0 |
| **NA-b-KRAS in HEPES@pH7.4** | d = 5±1 / *n.a.** | n = 1.46±0.01 / n_s_ = 1.33** | 0 /0 |
| **anti-IgG in PBS** | d = 7±1 / *n.a.** | n = 1.37±0.01 / n_s_ = 1.33** | 0 / 0 |

**n.a.:* non applicable; ** value taken from <https://refractiveindex.info/> and kept fixed during the modelling

The resulting thickness data obtained for the Cr/Au layers agree with those given by the SPR slides provider while the dielectric constants comply with tabulated data^[28]^. The thicknesses of the biolayer are in the 5 - 9 ± nm range with the error bars being one standard deviation over two sampled area of the specimen. Typical antibodies dimensions are about 14 nm · 8 nm · 4 nm, with the largest one being the distance between the binding sites. The evaluated thicknesses suggest that a single monolayer of nearly end-on antibodies is physisorbed on the surface^[14]^.

*The SPR estimate of the capturing biolayers surface coverage and density:* The resonant angular shift Δθ_SPR_ is modelled^[29]^ by the Jung function of the physisorbed antibody layer average thickness *d* and effective refractive index *n* by **Eq. S2**:

${\Delta\theta}_{SPR}=S\left( n-n_{s} \right)\left\lfloor1-exp\left( \frac{-d}{\delta} \right) \right\rfloor$ (**S2**)

where S the is the wavelength dependent SPR sensitivity coefficient which for the MP-SPR Navi^TM^ instrument at λ = 670 nm is 86.3 (°) per RIU (refractive index unit) and n_s_ is the refractive index of the bulk of the solvent. In the d ≪ δ approximation that holds as the antibody layers are less than 10 nm, $\left\lfloor1-exp\left( \frac{-d}{\delta} \right) \right\rfloor\approx\frac{d}{\delta}$, **Eq. S2** becomes:

${\Delta\theta}_{SPR}\approx S\left( n-n_{s} \right)\frac{d}{\delta}$ (**S3**).

Considering *dn/dC* the derivative of the refractive index with respect to the antibody concentration in the buffer solution, *C*, usually addressed as refractive index increment of the physisorbing biolayer^[30–32]^, can be calculated from the equation: *n = n_s_ + (dn/dC)c_b_*, with *c_b_* being the final concentration (or volume density) of the antibodies in the biolayer. The coefficient *dn/dC* can be taken in this case as 0.182 cm^3^·g^-1[33]^. The surface coverage of antibodies physisorbed on the Au surface can be assessed by the de Feijter equation^[34]^:

$c_{b}=\left( n-n_{s} \right)\cdot\left( \frac{dn}{dC} \right)^{-1}$ (**S4**)

so that the surface-mass density Γ = c_b_d, expressed in ng·cm^-2^, is approximated by:

$\Gamma= \frac{\delta}{S}\cdot{\Delta\theta}_{SPR}\cdot\left( \frac{dn}{dC} \right)^{-1}$ (**S5**)

and *Γ* can be estimated by measuring the SPR angular shift upon completion of the physisorption process, as:

$\Gamma={\Delta\theta}_{SPR}\cdot637 [{{10}^{-9}g}/{{cm}^{2}}]$ (**S6**).

From the data given in **Figure S2b, Figure S3b** and **Figure S4b**, the surface coverage estimate for the anti-HIV-1-p24 is (1.3 ± 0.1)∙10^12^ molecules/cm^2^ and the anti-IgG layer is (7.1 ± 0.4)∙10^11^ molecules/cm^2^ while for the b-KRAS on NA is (4.2 ± 0.1)∙10^12^ molecules/cm^2^ (the surface coverage of the sole NA is 3.6 ± 0.1∙10^12^/cm^2^). These data are summarized in **Table S3**.

From the Fresnel equations-based analysis (Winspall 3.02)^[27]^ of the SPR data of **Figure S2** - **S4**, an *n* between 1.37 ± 0.01 - 1.46 ± 0.01 for all the pristine biolayers is estimated (**Table S2)**. The biorecognition elements surface coverage, valued by the de Feijter equation^[34]^, is in the 10^11^-10^12^ molecules/cm^2^ range (**Table S3)** equivalent to ~10^4^ per micrometre squared which is close to the highest packing of proteins on a surface^[10]^. This is in line with literature data proving that a highly adhering deposit of a densely packed monolayer is obtained^[14,35–38]^. The physisorption can be accompanied by conformational changes and denaturation^[37]^.

***SN3. The SPR antigen sensing in the double-layer regime***

SPR typically assays at LODs^[10]^ in the 10^-9^ M concentration range^[39,40]^. Lower concentrations reaching down to 10^-16^ - 10^-12^ M^[41–46]^, rely on effects such as the localized surface plasmon resonance^[41,47]^, the plasmon-enhanced Raman spectroscopy^[42]^, or by engaging a sandwich assay with nanoparticles release^[43]^. When plasmon-enhanced effects are not present, SPR sensing is typically conducted using a plain capturing layer. In this scenario, a significant angular shift, is observed when the capturing layer is incubated in a solution containing an affinity ligand at a concentration in the 1·10^-9^ M range, at least. At these concentrations, a layer of antigens starts to form on top of the capturing biolayer. An example of the sensing in this regime is shown in **Figure S5a** where a physisorbed anti-IgG layer is exposed to the IgG ligand in the 2 - 440 ·10^-9^ M range. The relevant calibration curve is shown in **Figure S5b** along with the negative control experiment from which the noise level and its standard deviation can be estimated. The solid black line is the analytical modeling of the SPR sensing response based on the following five parameters logistic equation: *y=A_min_+(A_max_-A_min_)/(1+( x_0_/x)^h^)^s^* where *y* is the SPR angle shift *(Δθ_SPR_)*  and *x* is the IgG concentration. The fitting parameters *A_min_* and *A_max_* are, respectively, the initial response for *x* = 0 and the maximum response of the curve, *h* is defined as the Hill Coefficient, *s* is defined as the Asymmetry factor and *x_0_* is the inflection point of the curve. The fitting procedure was cross-validated, and the coefficients were adjusted depending on the residual errors in each iteration, obtaining the following parameters: A_min_ = (0 ± 0), A_max_ = (0.189 ± 0.029), x_0_ = (9·10^-9^ ± 1·10^-9^), h = (0.90 ± 0.61), s = (1.34 ± 2.37). The LOD level, computed as the negative control experiment average signal plus three times its standard deviation^[10]^, corresponds to a concentration of 2 ·10^-9^ M, being in line with literature data^[13,39,40,48–50]^. The signal in **Figure S5** saturates at 89·10^-9^ M corresponding to an IgG layer comprising (3.3 ± 0.8)∙10^11^ molecules/cm^2^, namely approximately one IgG every two anti-IgGs, as anti-IgG coverage is (7.1 ± 0.4)∙10^11^ molecules/cm^2^. This proves that with a standard SPR experiment the refracting index change is activable to the formation of a double-layer comprising a staking of the capturing antibody and of the affinity ligand.

When such pristine biolayers are exposed to their affinity ligand solutions, the SPR sensogram typically shows an appreciable angular shift only if a ligand concentration of at least few nM is injected. As an instance, in **Figure S5** the calibration curve for the SPR sensing of IgG at an anti-IgG capturing layer, shows a LOD of 2·10^-9^ M.

**
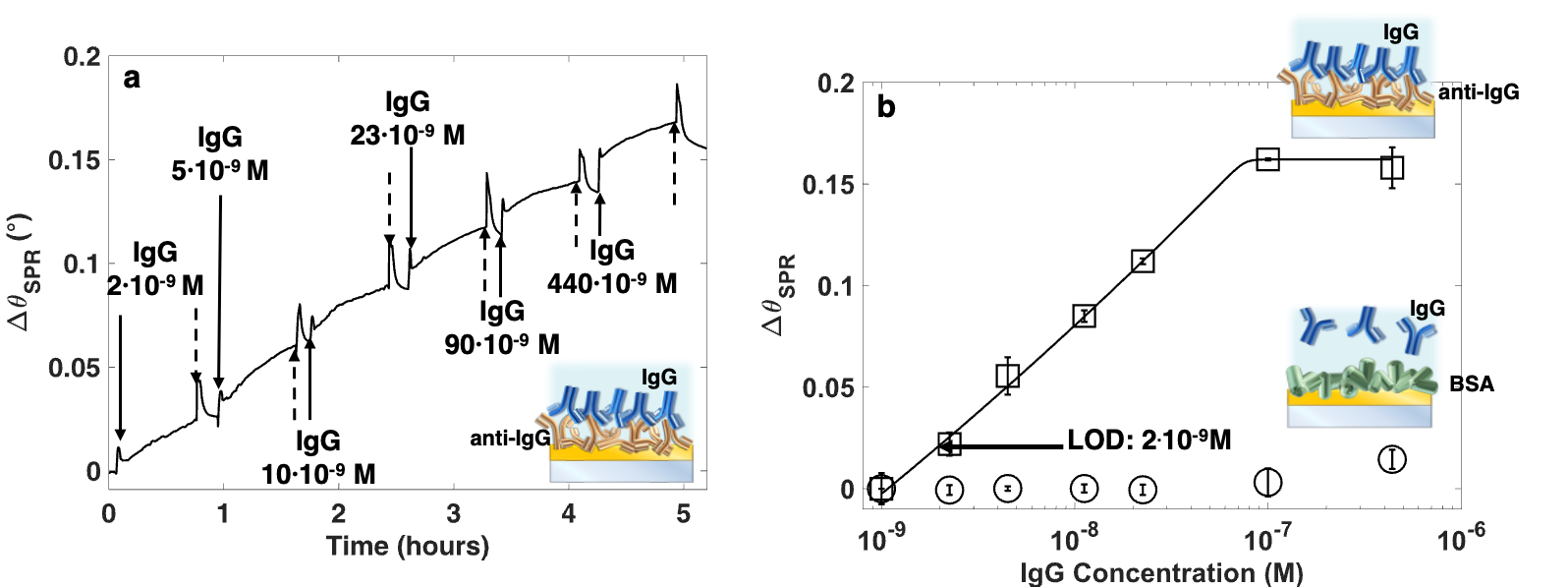
**

**Figure S5. SPR evaluation of the IgG layer binding on top of the anti-IgG capturing antibodies layer - (a)** SPR angle shift (Δθ_SPR_) during the binding of IgG at a physisorbed anti-IgG layer with a density of (7.1±0.4)∙10^11^ molecules/cm^2^. The solid arrows indicate the injection of 0.1 mL of the IgG PBS buffer solutions (pH=7.4, i_s_=162·10^-3^M) in the 2 – 440 nM range, while the dashed arrows indicate the washing with 0.1 mL of bare PBS. **(b)** IgG calibration curve (Δθ_SPR_ vs. IgG concentration, hollow black squares) along with the negative control experiment (hollow black circles) measured exposing a BSA physisorbed layer (BSA 10 mg/mL in PBS for 2 hours) exposed to the same IgG solutions.

***SN4. The pH conditioning of the capturing biolayers***

A conditioning of the capturing antibodies or of the Na-b-KRAS protein-probe complexes biolayers at a pH different from the physiological one at which physisorption occurs, can change completely their affinity binding sensitivity. In **Figure S6a** a typical conditioning experiment of the anti-HIV-1-p24 biolayer, are shown. The sensograms start with the baseline signal recorded on the pristine as deposited biolayer in HEPES@pH7.4. To condition the layer a 0.1 mL aliquot of an HEPES buffer with the same i_s_=150 mM but at pH 6 (HEPES@pH6), is injected and let to rest in contact with the film for 30 minutes. Afterwards the film is washed by injecting 0.1 mL of the physiological HEPES@pH7.4 buffer. The SPR differential signal measured in HEPES@pH7.4, before and after the pH conditioning, shows that the anti-HIV-1-p24 layer during the conditioning at pH 6, undergoes a net permanent increase in effective refractive index. A lower increase is seen when the conditioning is carried out in HEPES@pH8 (pH 8 and ionic strength i_s_=150 mM). In **Figure S6b** the same conditioning at pH 6 and pH 8 is carried out on the NA-b-KRAS probe layer. During the pH-conditioning the *in-situ* and *operando* control of the temperature in the SPR cell, allowed to actually prove that the temperature oscillates in the 21.1 ± 0.1 °C range. In **Figure S6c** and **Figure S6d** the temperature oscillations during the pH conditioning is shown. As it is apparent, the oscillations of the temperature are within the instrumental specification, namely ± 0.1 °C, and are the same at all the stages of the pH-conditioning. This proves the very high control of the temperature that was in place. Moreover, the temperature besides being very stable is also low enough (21.1 °C) not to induce, *per se*, changes in the properties of the proteins physisorbed on the detecting surface. The effect of the conditioning protocol is, hence, to be ascribed to the exposure for 10 minutes to a pH shift. Such a conditioning step is also not generating a permanent protonation/deprotonation of the proteins on the surface as this reversible equilibrium is brough back, after conditioning, by bringing the pH its original value (pH=7.4) by a thoroughly rinsing in HEPES@pH7.4. This is proven by the infrared characterization proposed in the **SN15** subsection section entitled “*Assessment of the capturing layer protonation/deprotonation before and after pH-conditioning”.* All the data gathered concur to demonstrate that the changes in the recognition elements biolayers induced by the pH-conditioning are not associated to a straightforward irreversible protonation/deprotonation effect.

**
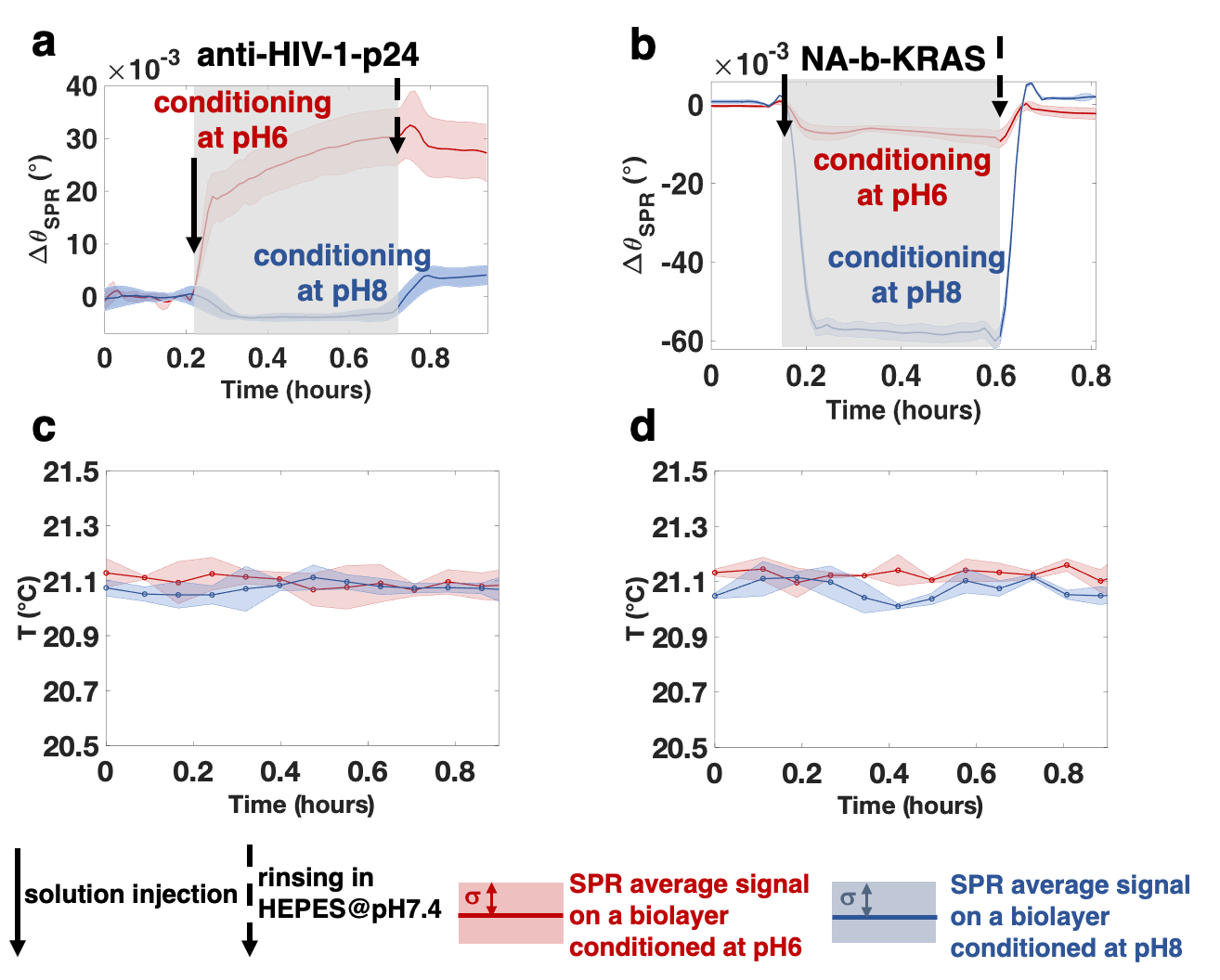
**

**Figure S6**. **The biolayers pH conditioning -** **(a)** The sensograms (each averaged over 6 replicates) of the anti-HIV-1-p24 biolayer measured during the conditioning in HEPES@pH6 and in HEPES@pH8 are presented; **(b)** same sensograms (each averaged over 4 replicates) as in panel **(a)** are shown for the conditioning of the NA-b-KRAS biolayer. Traces in **(c)** and **(d)** are the temperature controlled and measured *in-situ* in the SPR cell during the pH-conditioning.

***SN5. SPR raw data of the single/few-molecules sensing***

In the following the Δ*θ*_SPR_ *vs.* time sensograms for the sensing on the three different biosystems are shown. Each figure comprises the number of replicates performed on each sensing system. In each of the following figures it is specified if the biolayer is pH-conditioned or not. The reference is the θ_SPR_ of the biofunctionalized slide kept in HEPES@pH7.4 which is the buffer solution used during the physisorption and all throughout the experiments. Each trace is acquired in duplicates by sampling the 0.4 cm^2^ large slide in two points set 3 mm apart. After measuring the baseline in HEPES@pH7.4, the non-binding molecule (10^-15^ M in HEPES@pH7.4) is injected. This is also the interferent present in all the sensing solutions. After incubation a first dashed arrow indicates a washing step followed (second solid arrow) by the injection of 1·10^-20^ M (approximated to the nearest whole as 1 ± 1 protein, single-molecule sensing) of the affinity binding molecule. A second dashed arrow indicates a further washing step followed by the injection (third solid arrow) of 1·10^-19^M (6 ± 2 proteins, few-molecules sensing) of the affinity binding solution. After a third washing step the injection of a 1·10^-7^ M (10^13^ protein in the 0.1 mL injected volume, double-layer sensing regime) of the affinity binding solution is injected and finally washed. In the upper panels the whole measured curve is shown, while in the middle panels the sensogram region up to the 10^-19^ M analyte injection is zoomed in. Experiments are also performed in directly diluted human serum. The specificity of each set of experiments are detailed in the captions.

*The anti-HIV-1-p24 capturing biolayer sensing in HEPES@pH7.4*


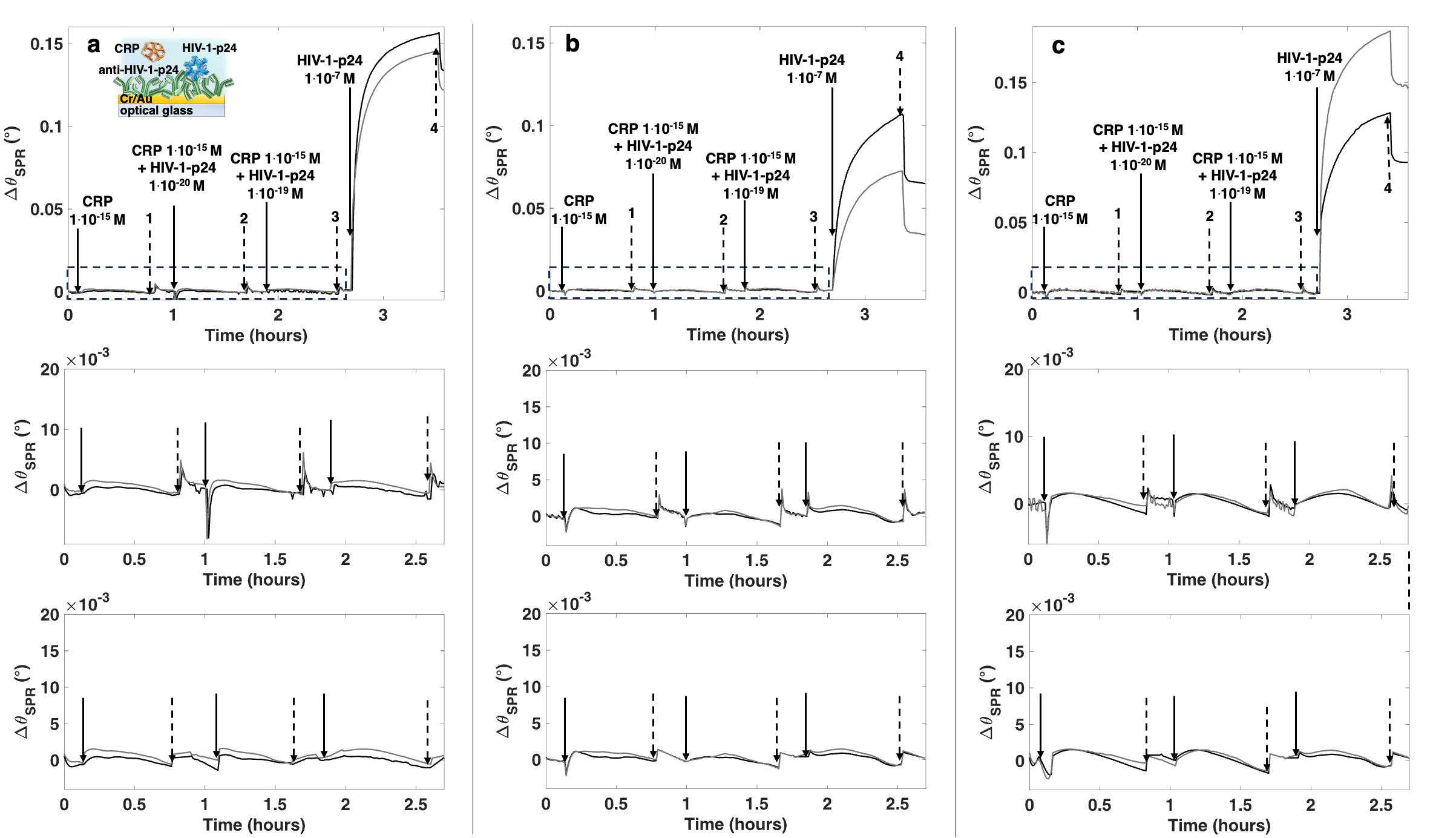


**Figure S7: Sensing raw and smoothed data on non-conditioned anti-HIV-1-p24 capturing biolayer -** First **(a)**, second **(b),** and third **(c)** SPR sensogram replicate of the pristine non-conditioned anti-HIV-1-p24 biolayer. The reference is the θ_SPR_ of the biofunctionalized slide kept in HEPES@pH7.4 which is the buffer solution used during the physisorption and all throughout the experiments. Each trace is acquired in duplicates by sampling the 0.4 cm^2^ wide slide in two points set 3 mm apart. The first solid arrow indicates the injection (0.1 mL) of a 10-^15^ M CRP HEPES@pH7.4 solution, with the non-binding CRP serving as interferent. After 40 minutes of incubation a first dashed arrow indicates a washing step (0.1 mL of HEPES@pH7.4), followed, after 10 minutes (second solid arrow) by the injection of a HIV-1-p24 10^-20^M in CRP 10-^15^M HEPES@pH7.4 solution (1±1 protein in the 0.1 mL injected volume). A second dashed arrow indicates a further washing step (0.1 mL of HEPES@pH7.4) followed by the injection (third solid arrow) of a HIV-1-p24 10^-19^M in a CRP 10-^15^M HEPES@pH7.4 solution (6±2 proteins in the 0.1 mL injected volume). After a third washing step the injection of a HIV-1-p24 10^-7^M in HEPES@pH7.4 (10^13^ protein in the 0.1 mL injected volume) is injected and finally washed. In the upper panels the whole measured curve is shown, while in the middle panels the sensogram region up to the 10^-19^M analyte injection is zoomed-in. A light third-degree smoothing routine is performed on the whole curve except the regions where the injection of a new batch occurs. Here a one-degree smoothing routine is used.

***
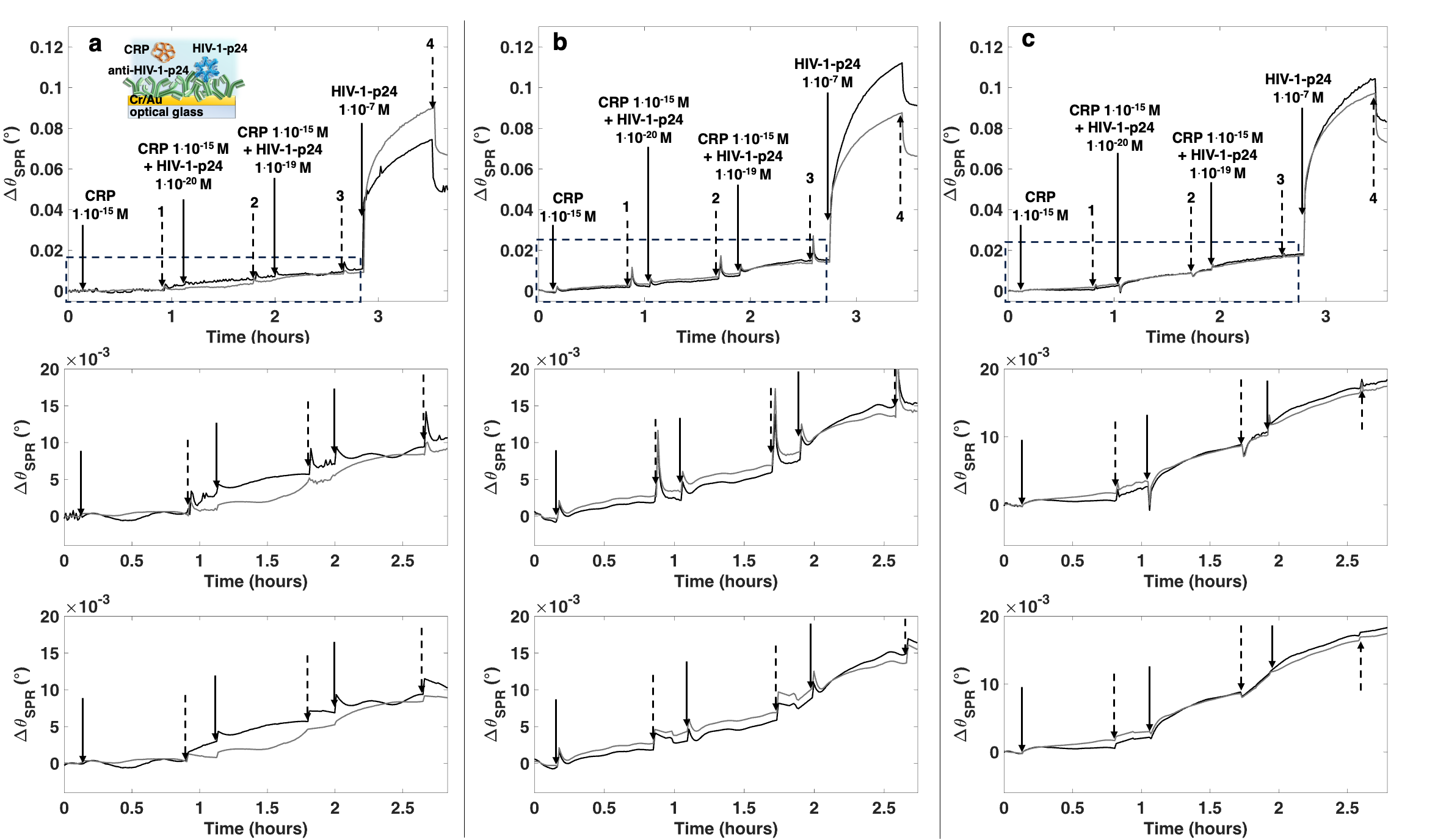
***

**Figure S8. Sensing raw and smoothed data on anti-HIV-1-p24 capturing biolayer conditioned at pH 6 -** First **(a)**, second **(b),** and third **(c)** SPR sensogram replicate of the anti-HIV-1-p24 biolayer conditioned at pH 6. The reference is the θ_SPR_ of the biofunctionalized slide kept in HEPES@pH7.4 which is the buffer solution used during the physisorption and all throughout the experiments. Each trace is acquired in duplicates by sampling the 0.4 cm^2^ wide slide in two points set 3 mm apart. The first solid arrow indicates the injection (0.1 mL) of a 10-^15^M CRP HEPES@pH7.4 solution. The non-binding CRP serves as interferent. After 40 minutes of incubation a first dashed arrow indicates a washing step (0.1 mL of HEPES@pH7.4), followed, after 10 minutes (second solid arrow) by the injection of a HIV-1-p24 10^-20^M in CRP 10-^15^M HEPES@pH7.4 solution (1±1 protein in the 0.1 mL injected volume). A second dashed arrow indicates a further washing step (0.1 mL of HEPES@pH7.4) followed by the injection (third solid arrow) of a HIV-1-p24 10^-19^M in a CRP 10-^15^M HEPES@pH7.4 solution (6±2 protein in the 0.1 mL injected volume). After a third washing step the injection of a HIV-1-p24 10^-7^M in HEPES@pH7.4 (10^13^ protein in the 0.1 mL injected volume) is injected and finally washed. In the upper panels the whole measured curve is shown, while in the middle panels the sensogram region up to the 10^-19^M analyte injection is zoomed-in. A light third-degree smoothing routine is performed on the whole curve except the regions where the injection of a new batch occurs. Here a one-degree smoothing routine is used.

***
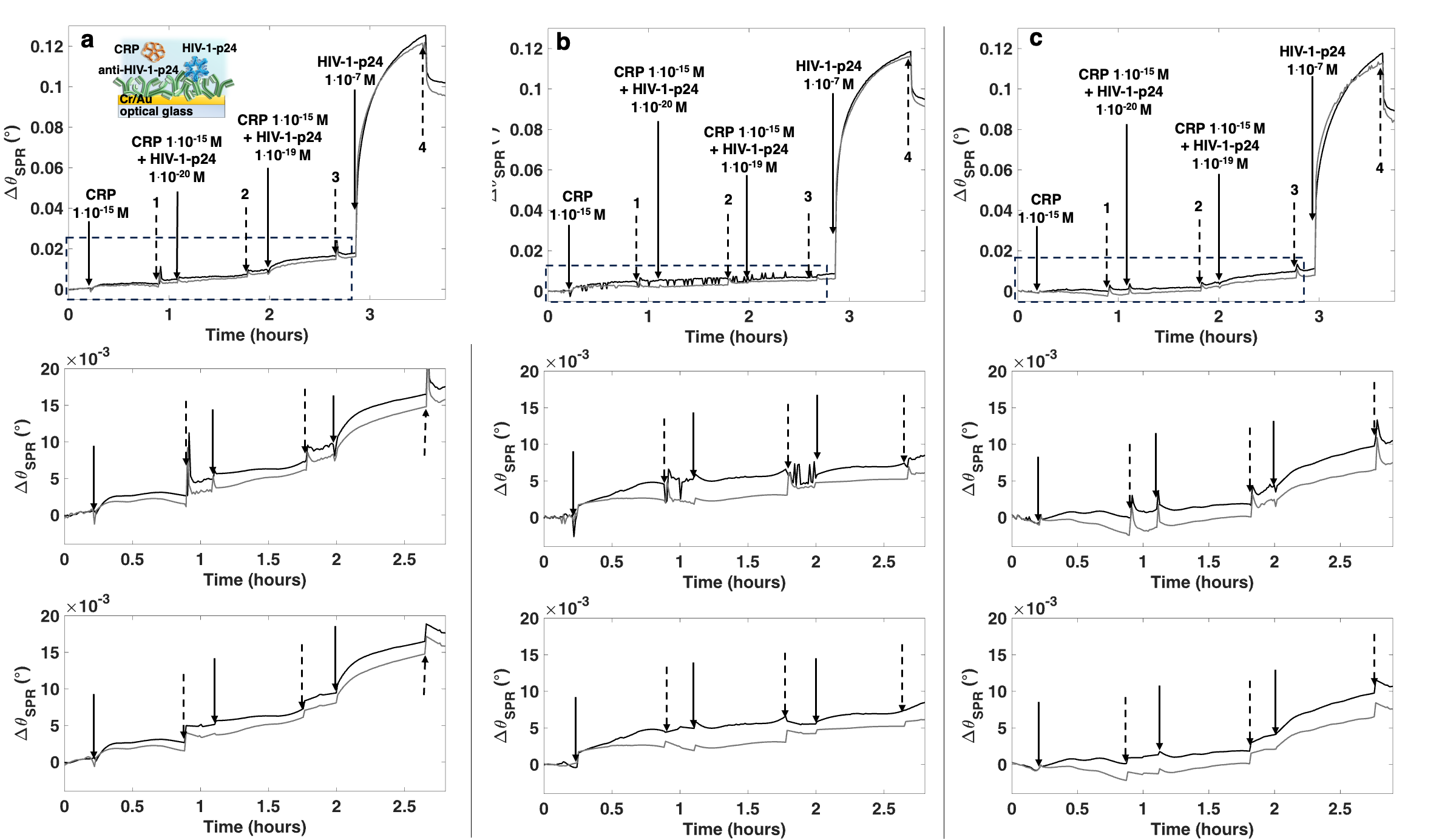
***

**Figure S9. Sensing raw and smoothed data on anti-HIV-1-p24 capturing biolayer conditioned at pH 8 -** First **(a)**, second **(b),** and third **(c)** SPR sensogram replicate of the anti-HIV-1-p24 biolayer conditioned at pH 8. The reference is the θ_SPR_ of the biofunctionalized slide kept in HEPES@pH7.4 which is the buffer solution used during the physisorption and all throughout the experiments. Each trace is acquired in duplicates by sampling the 0.4 cm^2^ wide slide in two points set 3 mm apart. The first solid arrow indicates the injection (0.1 mL) of a 10-^15^M CRP HEPES@pH7.4 solution. The non-binding CRP serves as interferent. After 40 minutes of incubation a first dashed arrow indicates a washing step (0.1 mL of HEPES@pH7.4), followed, after 10 minutes (second solid arrow) by the injection of a HIV-1-p24 10^-20^M in CRP 10-^15^M HEPESpH@7.4 solution (1±1 protein in the 0.1 mL injected volume). A second dashed arrow indicates a further washing step (0.1 mL of HEPES@pH7.4) followed by the injection (third solid arrow) of a HIV-1-p24 10^-19^M in a CRP 10-^15^M HEPES@pH7.4 solution (6±2 protein in the 0.1 mL injected volume). After a third washing step the injection of a HIV-1-p24 10^-7^M in HEPES@pH7.4 (10^13^ protein in the 0.1 mL injected volume) is injected and finally washed. In the upper panels the whole measured curve is shown, while in the middle panels the sensogram region up to the 10^-19^M analyte injection is zoomed-in. A light third-degree smoothing routine is performed on the whole curve except the regions where the injection of a new batch occurs. Here a one-degree smoothing routine is used.

*The anti-HIV-1-p24 capturing biolayer sensing in human serum*


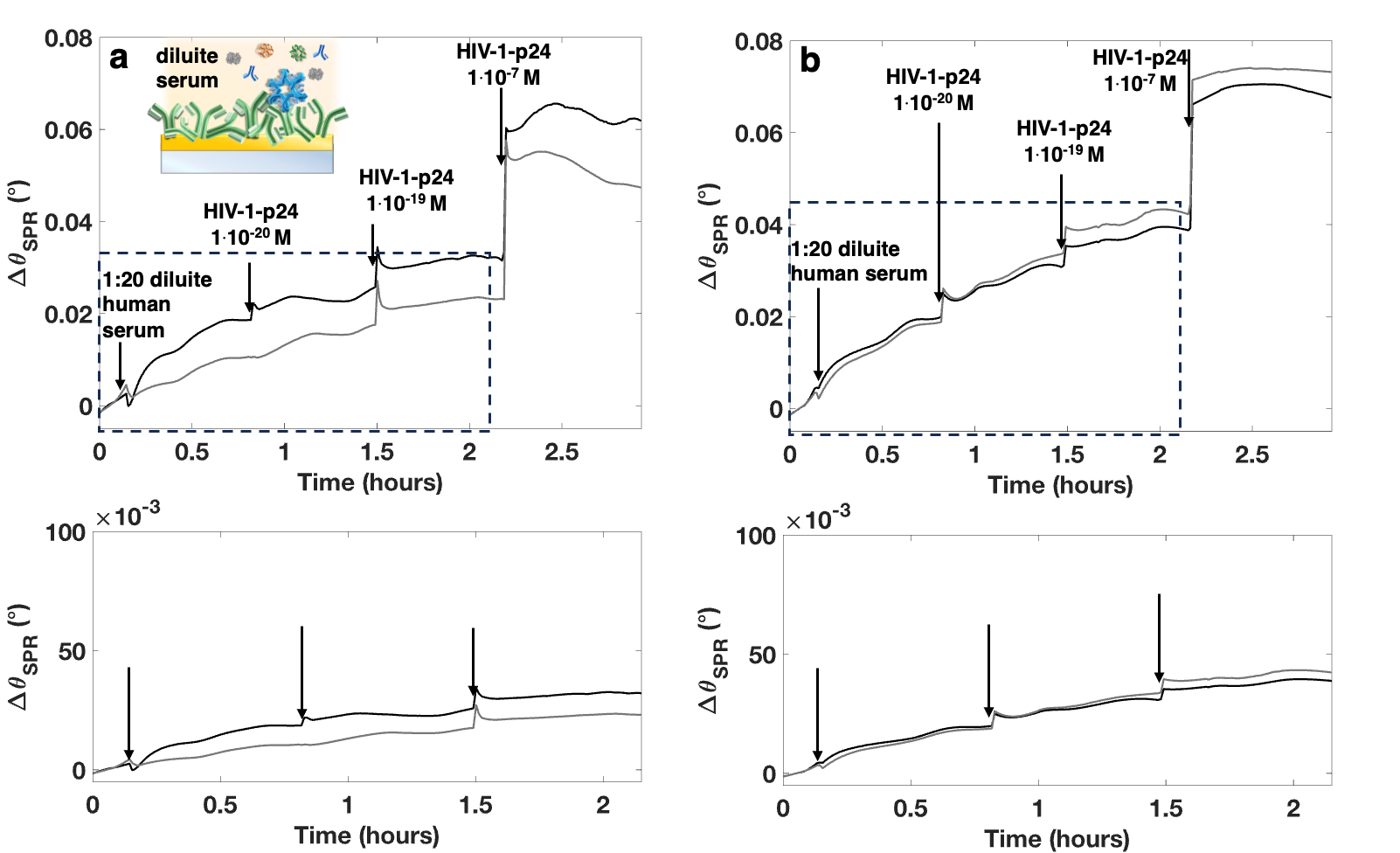


**Figure S10. Sensing raw data on non-conditioned anti-HIV-1-p24 capturing biolayer assessed in diluted human serum -** First **(a)** and second **(b)** SPR sensogram replicate of the pristine non-conditioned anti-HIV-1-p24 biolayer tested in human serum diluted 1:20 in HEPES@pH7.4. The reference is the θ_SPR_ of the biofunctionalized slide kept in the diluted serum used all throughout the experiments. Each trace is acquired in duplicates by sampling the 0.4 cm^2^ wide slide in two points set 3 mm apart. The first solid arrow indicates the injection (0.1 mL ) of the human serum diluted 1:20 in HEPES@pH7.4 solution. After 40 minutes of incubation a 0.1 mL diluted serum solution of HIV-1-p24 10^-20^M (1±1 protein in the 0.1 mL injected volume) is injected and let to incubate for 40 minutes. Afterwards a 0.1 mL solution of the HIV-1-p24 10^-19^M (6±2 proteins) in the diluted serum is injected and incubated for 40 minutes. The experiment concludes with the injection of the 0.1 mL solution of diluted serum added with the HIV-1-p24 10^-7^M. In the upper panels the whole measured curve is shown, while in the middle panels the sensogram region up to the 10^-19^M analyte injection is zoomed-in.

**
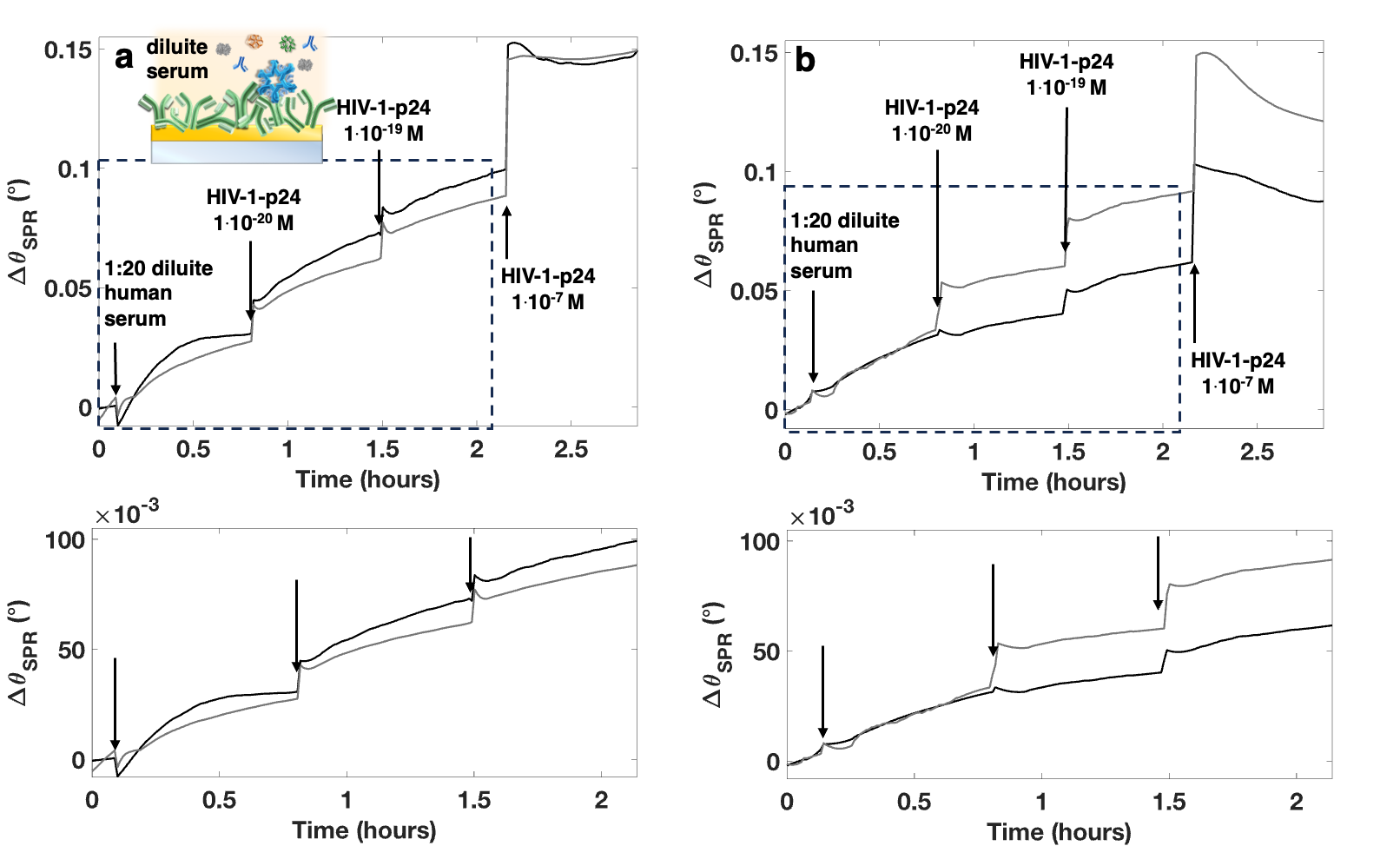
**

**Figure S11. Sensing raw data on anti-HIV-1-p24 capturing** **biolayer conditioned at pH 6 assessed in diluted human serum -** First **(a)** and second **(b)** SPR sensogram replicate of an anti-HIV-1-p24 biolayer conditioned at pH 6 and tested in human serum diluted 1:20 in HEPES@pH7.4. The reference is the θ_SPR_ of the biofunctionalized slide kept in the diluted serum used all throughout the experiments. Each trace is acquired in duplicates by sampling the 0.4 cm^2^ wide slide in two points set 3 mm apart. The first solid arrow indicates the injection (0.1 mL) of the human serum diluted 1:20 in HEPES@pH7.4 solution. After 40 minutes of incubation a 0.1 mL diluted serum solution of HIV-1-p24 10^-20^M (1±1 protein in the 0.1 mL injected volume) is injected and let to incubate for 40 minutes. Afterwards a 0.1 mL solution of the HIV-1-p24 10^-19^M (6±2 proteins) in the diluted serum is injected and incubated for 40 minutes. The experiment concludes with the injection of the 0.1 mL solution of diluted serum added with the HIV-1-p24 10^-7^M. In the upper panels the whole measured curve is shown, while in the middle panels the sensogram region up to the 10^-19^M analyte injection is zoomed-in.

The single-molecule sensing takes just 3 steps: *i)* the conditioning (6 minutes), *ii)* the baseline in serum (32 minutes to reach 95% of the signal) and the *iii)* single-molecule sensing (32 minutes to reach 95% of the signal). Overall lasting one hour and 10 minutes.

**
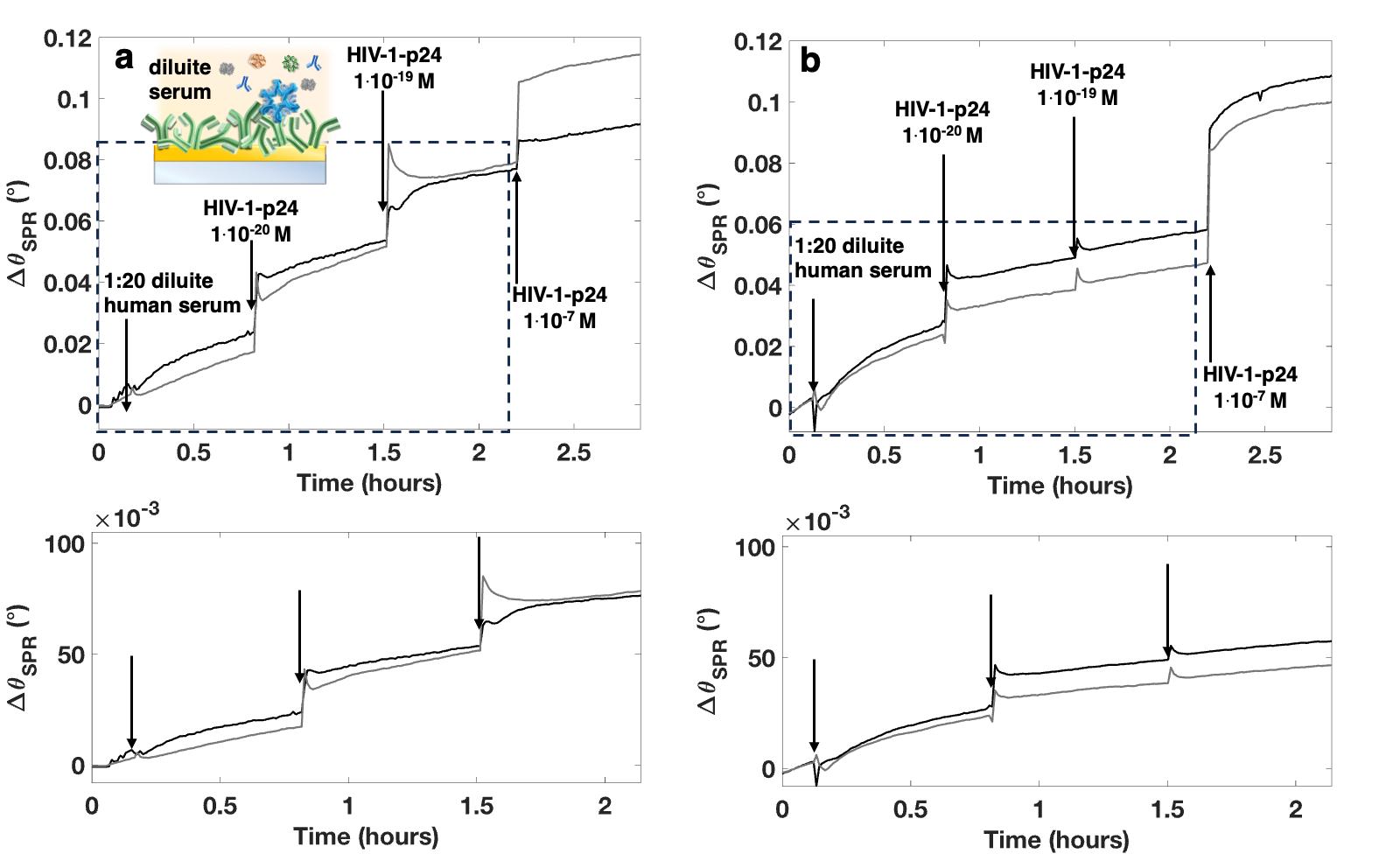
**

**Figure S12. Sensing raw data on anti-HIV-1-p24 capturing biolayer conditioned at pH 8 assessed in diluted human serum -** First **(a)** and second **(b)** SPR sensogram replicate of an anti-HIV-1-p24 biolayer conditioned at pH 8 and tested in human serum diluted 1:20 in HEPES@pH7.4. The reference is the θ_SPR_ of the biofunctionalized slide kept in the diluted serum used all throughout the experiments. Each trace is acquired in duplicates by sampling the 0.4 cm^2^ wide slide in two points set 3 mm apart. The first solid arrow indicates the injection (0.1 mL) of the human serum diluted 1:20 in HEPES@pH7.4 solution. After 40 minutes of incubation a 0.1 mL diluted serum solution of HIV-1-p24 10^-20^M (1±1 protein in the 0.1 mL injected volume) is injected and let to incubate for 40 minutes. Afterwards a 0.1 mL solution of the HIV-1-p24 10^-19^M (6±2 proteins) in the diluted serum is injected and incubated for 40 minutes. The experiment concludes with the injection of the 0.1 mL solution of diluted serum added with the HIV-1-p24 10^-7^M. In the upper panels the whole measured curve is shown, while in the middle panels the sensogram region up to the 10^-19^M analyte injection is zoomed-in.

*The anti-IgG capturing biolayer exposed to multiple pH shifts*

**
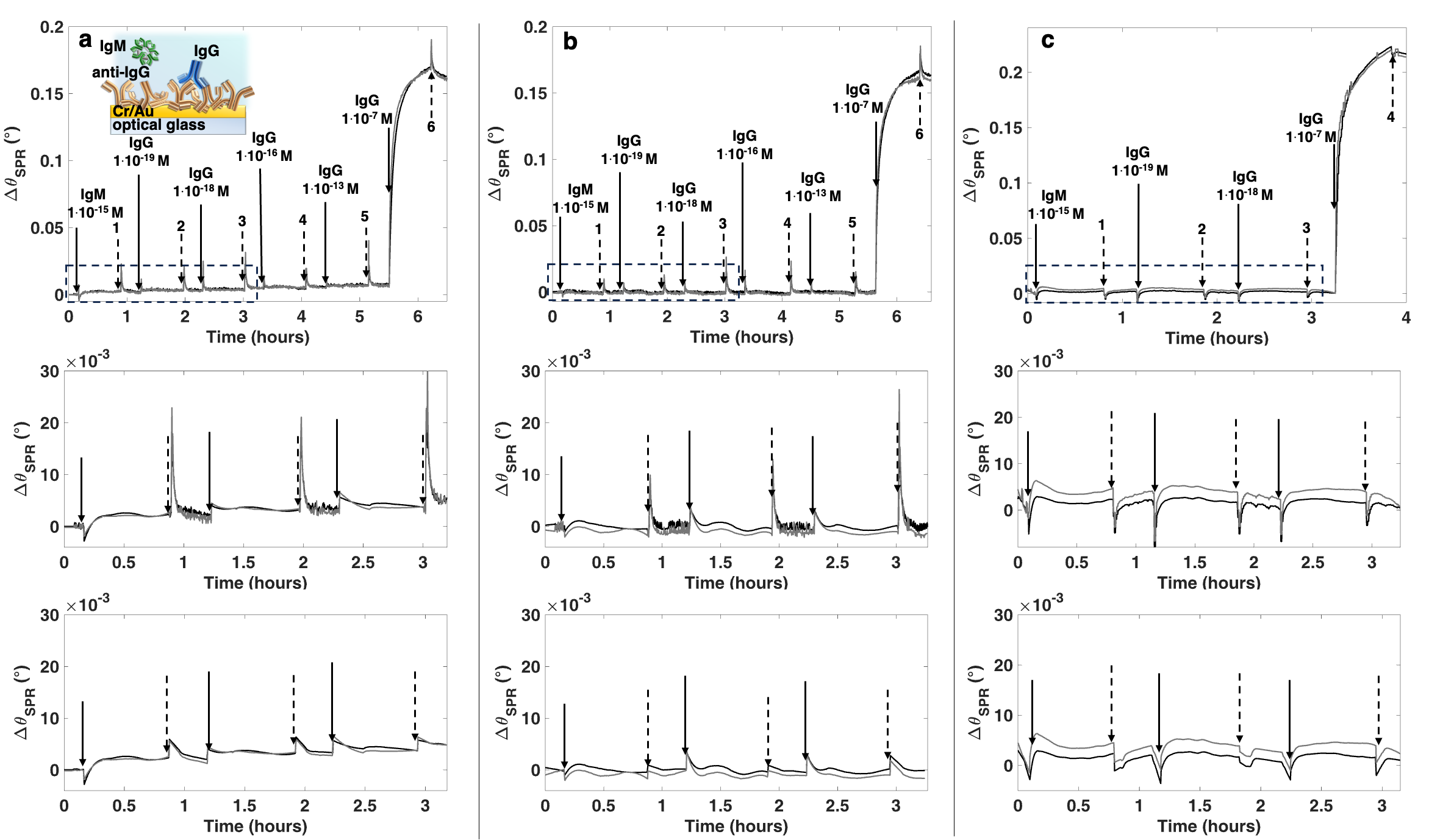
**

**Figure S13. Sensing raw and smoothed data on non-conditioned anti-IgG capturing biolayer -** First **(a)**, second **(b),** and third **(c)** SPR sensogram replicate of the non-conditioned anti-IgG biolayer. The reference is the θ_SPR_ of the biofunctionalized slide kept in HEPES@pH7.4 which is the buffer solution used during the physisorption and all throughout the experiments. Each trace is acquired in duplicates by sampling the 0.4 cm^2^ wide slide in two points set 3 mm apart. The biolayers are exposed to a sensing protocol encompassing at first the injection of the non-binding IgM (10^-15^M) in 0.1 mL of HEPES@pH7.4. After 40 minutes, a rinsing in HEPES@pH7.4 for 10 minutes. Then, IgG 10^-20^M (1±1), 10^-19^M (6±2), 10^-18^M, 10^-17^M, 10^-13^M, and 10^-7^M solutions in 0.1 mL of HEPES@pH7.4 are injected and incubated for 40 minutes and then rinsed in HEPES@pH7.4 for 10 minutes. Black solid arrows indicate the analyte injection step while the dashed lines indicate the starting of a washing step in HEPES@pH7.4. In the upper panels the whole measured curve is shown, while in the middle panels the sensogram region up to the 10^-19^M analyte injection is zoomed-in. A light third-degree smoothing routine is performed on the whole curve except the regions where the injection of a new batch occurs. Here a one-degree smoothing routine is used.

**
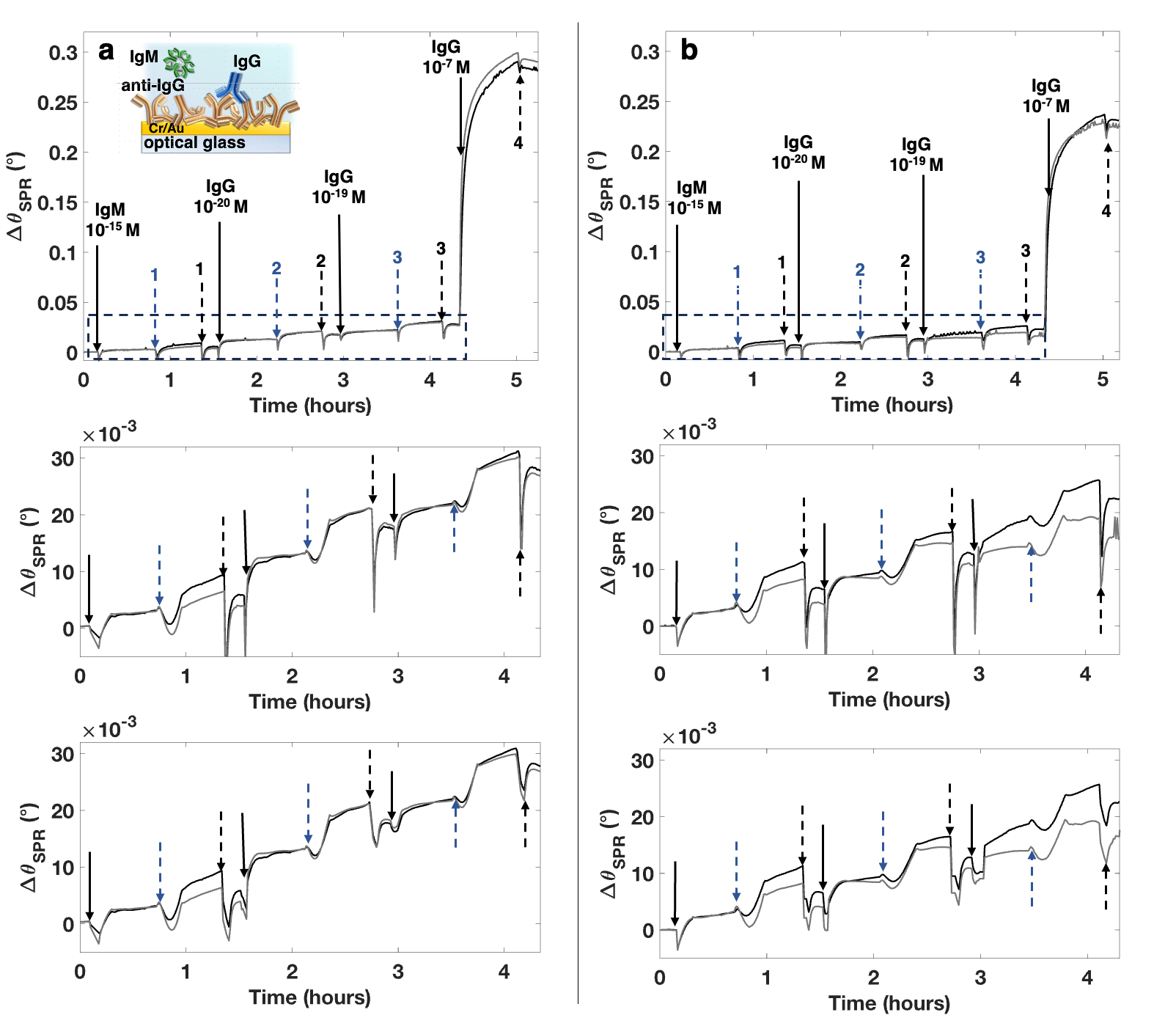
**

**Figure S14. Sensing raw and smoothed data on anti-IgG capturing biolayer conditioned at pH 6 at each sensing step (multiple conditioning) -**  First **(a)** and second **(b)** SPR sensogram replicate of the anti-IgG biolayer undergoing multiple conditioning at pH 6. The reference is the θ_SPR_ of the biofunctionalized slide kept in HEPES@pH7.4 which is the buffer solution used during the physisorption and all throughout the experiments. Each trace is acquired in duplicates by sampling the 0.4 cm^2^ wide slide in two points set 3 mm apart. The biolayers, after a first conditioning at pH 6 for 30 minutes and washing in HEPES@pH7.4, are exposed to a sensing protocol encompassing at first the injection of the non-binding IgM (10^-15^M) in 0.1 mL of HEPES@pH7.4. After incubating for 40 minutes, a further conditioning step in HEPES@pH6 is carried out lasting 30 minutes, which is followed by a rinsing in HEPES@pH7.4 for 10 minutes. Then, IgG 10^-20^M (1±1), 10^-19^M (6±2), 10^-18^M, 10^-17^M, 10^-13^M, and 10^-7^M solutions in 0.1 mL of HEPES@pH7.4 are injected and each one is followed by a conditioning at pH 6 and a rinsing step. Black solid arrows indicate the analyte injection step while the dashed lines indicate the starting of a washing step in HEPES@pH7.4. The dashed blue arrows indicate the starting of the conditioning step at pH 6. In the upper panels the whole measured curve is shown, while in the middle panels the sensogram region up to the 10^-19^M analyte injection is zoomed-in. A light third-degree smoothing routine is performed on the whole curve except the regions where the injection of a new batch occurs. Here a one-degree smoothing routine is used.


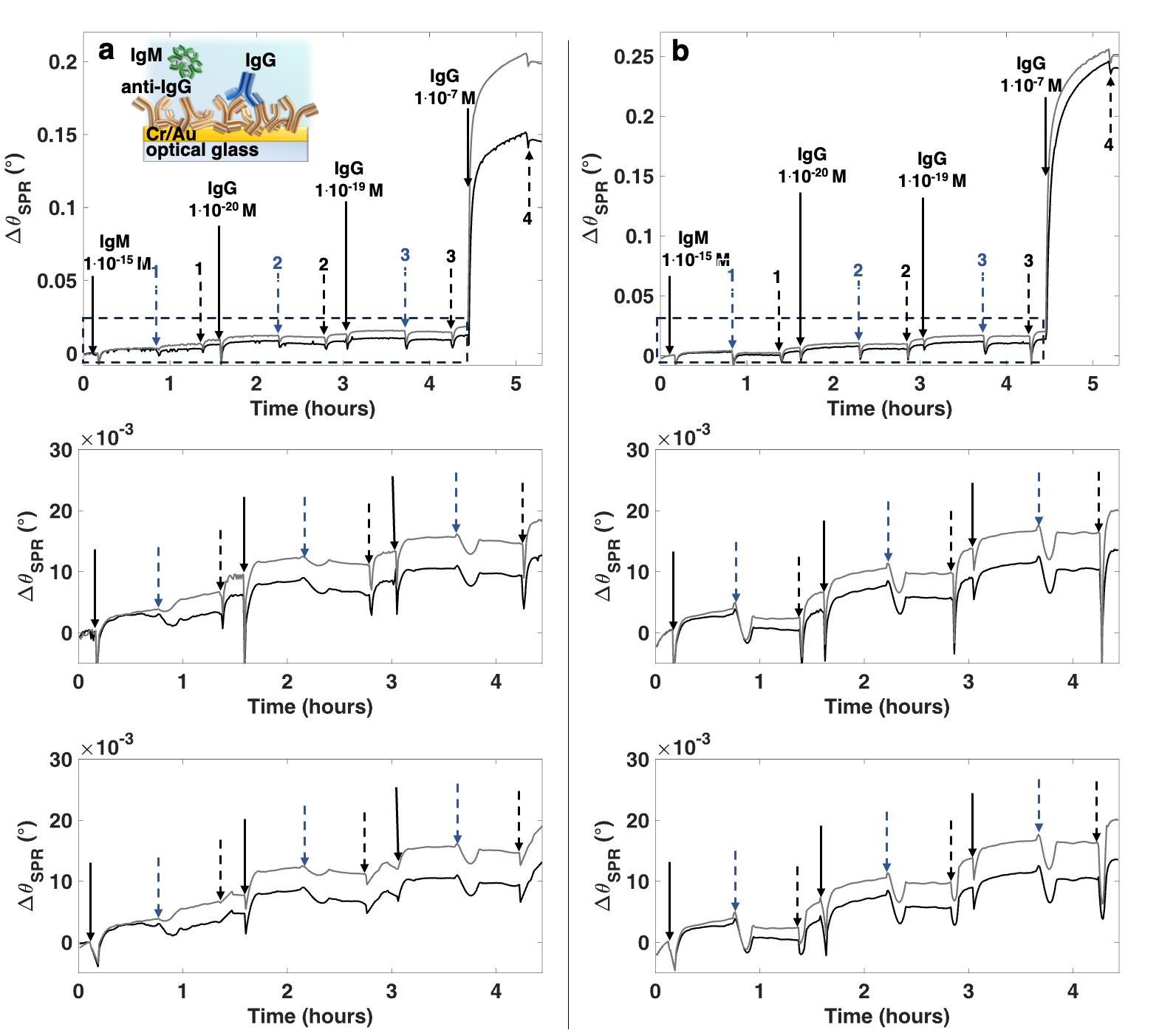


**Figure S15. Sensing raw and smoothed data on anti-IgG capturing biolayer conditioned at pH 8 at each sensing step (multiple conditioning)-** First **(a)** and second **(b)** SPR sensogram replicate of the anti-IgG biolayer undergoing multiple conditioning at pH 8. The reference is the θ_SPR_ of the biofunctionalized slide kept in HEPES@pH7.4 which is the buffer solution used during the physisorption and all throughout the experiments. Each trace is acquired in duplicates by sampling the 0.4 cm^2^ wide slide in two points set 3 mm apart. The biolayers, after a first conditioning at pH 8 for 30 minutes and washing in HEPES@pH7.4, are exposed to a sensing protocol encompassing at first the injection of the non-binding IgM (10^-15^M) in 0.1 mL of HEPES@pH7.4. After incubating for 40 minutes, a further conditioning step in HEPES@pH8 is carried out lasting 30 minutes, which is followed by a rinsing in HEPES@pH7.4 for 10 minutes. Then, IgG 10^-20^M (1±1), 10^-19^M (6±2), 10^-18^M, 10^-17^M, 10^-13^M, and 10^-7^M solutions in 0.1 mL of HEPES@pH7.4 are injected and each one is followed by a conditioning at pH 8 and a rinsing step. Black solid arrows indicate the analyte injection step while the dashed lines indicate the starting of a washing step in HEPES@pH7.4. The dashed blue arrows indicate the starting of the conditioning step at pH 8. In the upper panels the whole measured curve is shown, while in the middle panels the sensogram region up to the 10^-19^M analyte injection is zoomed-in. A light third-degree smoothing routine is performed on the whole curve except the regions where the injection of a new batch occurs. Here a one-degree smoothing routine is used.


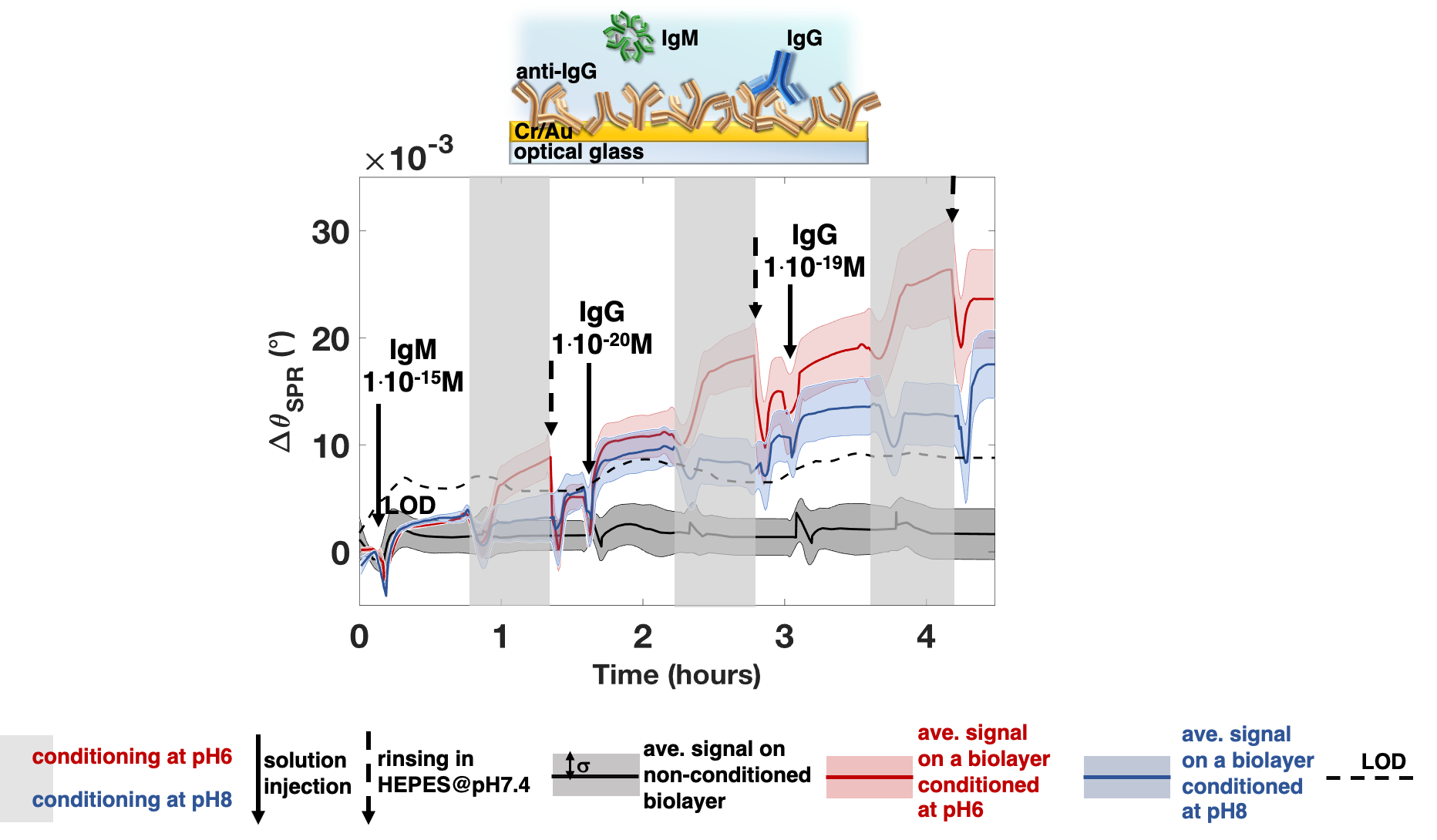


**Figure S16. SPR single/few-molecules detections of IgG in a physiological buffer -** Average sensing curves of non-conditioned (black-lines) and pH-conditioned biolayers (red-lines: pH6, blue lines: pH8). Anti-IgG capturing layer exposed to the non-binding IgM (1·10^-15^M) and to IgG (1·10^-20^M and 1·10^-19^M); vertical shadowing indicates pH conditioning carried out before each sensing step (4 or 6 replicates, raw-data in **figs.S13-S15**).The colored shadings represent one standard deviation, while the dashed black-lines are the LOD levels, calculated as the average values of each black-curve (taken as the measure noise) plus three times the standard deviation. All the solutions are in an HEPES@pH7.4 buffer (i_s_ = 150 mM) or in diluted human serum. The solid arrows indicate the injection of 1 mL of a given solution, while the rinsing steps are indicated by dashed arrows.

*The NA-b-KRAS probe biolayer*


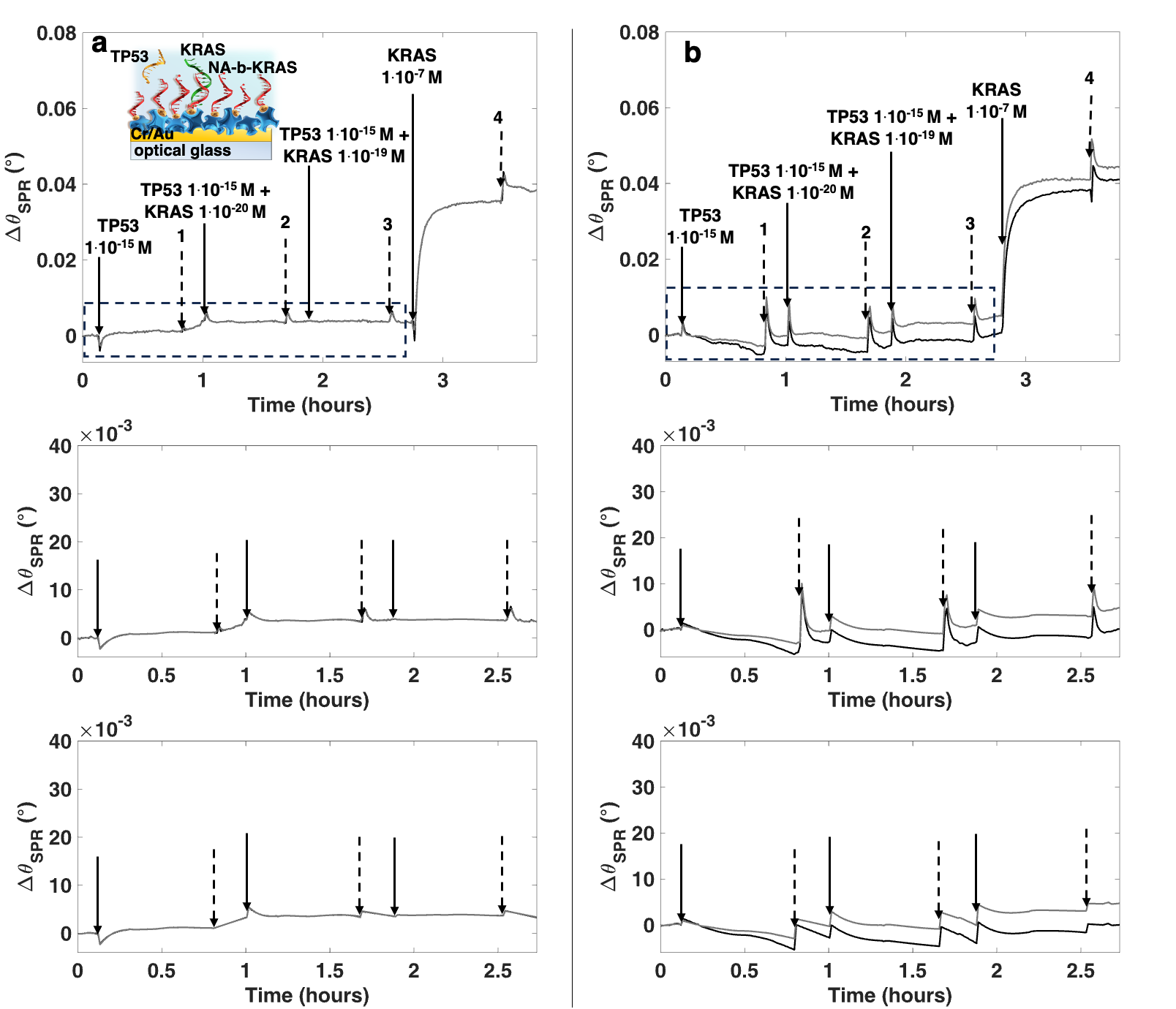


**Figure S17. Sensing raw and smoothed data on non-conditioned NA-b-KRAS probe biolayer -** First **(a)**, second **(b),** and third **(c)** SPR sensogram replicate of the pristine non-conditioned NA-b-KRAS biolayer. The reference is the θ_SPR_ of the biofunctionalized slide kept in HEPES@pH7.4 which is the buffer solution used during the physisorption and all throughout the experiments. Each trace is acquired in duplicates by sampling the 0.4 cm^2^ wide slide in two points set 3 mm apart. The first solid arrow indicates the injection (0.1 mL) of a 10-^15^M TP53 HEPES@pH7.4 solution, with the non-binding TP53 serving as interferent. After 40 minutes of incubation a first dashed arrow indicates a washing step (0.1 mL of HEPES@pH7.4), followed, after 10 minutes (second solid arrow) by the injection of a KRAS 10^-20^M in TP53 10-^15^M HEPES@pH7.4 solution (1±1 protein in the 0.1 mL injected volume). A second dashed arrow indicates a further washing step (0.1 mL of HEPES@pH7.4) followed by the injection (third solid arrow) of a KRAS 10^-19^M in a TP53 10-^15^M HEPES@pH7.4 solution (6±2 proteins in the 0.1 mL injected volume). After a third washing step the injection of a KRAS 10^-7^M in HEPES@pH7.4 is injected and finally washed. In the upper panels the whole measured curve is shown, while in the middle panels the sensogram region up to the 10^-19^M analyte injection is zoomed-in. A light third-degree smoothing routine is performed on the whole curve except the regions where the injection of a new batch occurs. Here a one-degree smoothing routine is used.

***
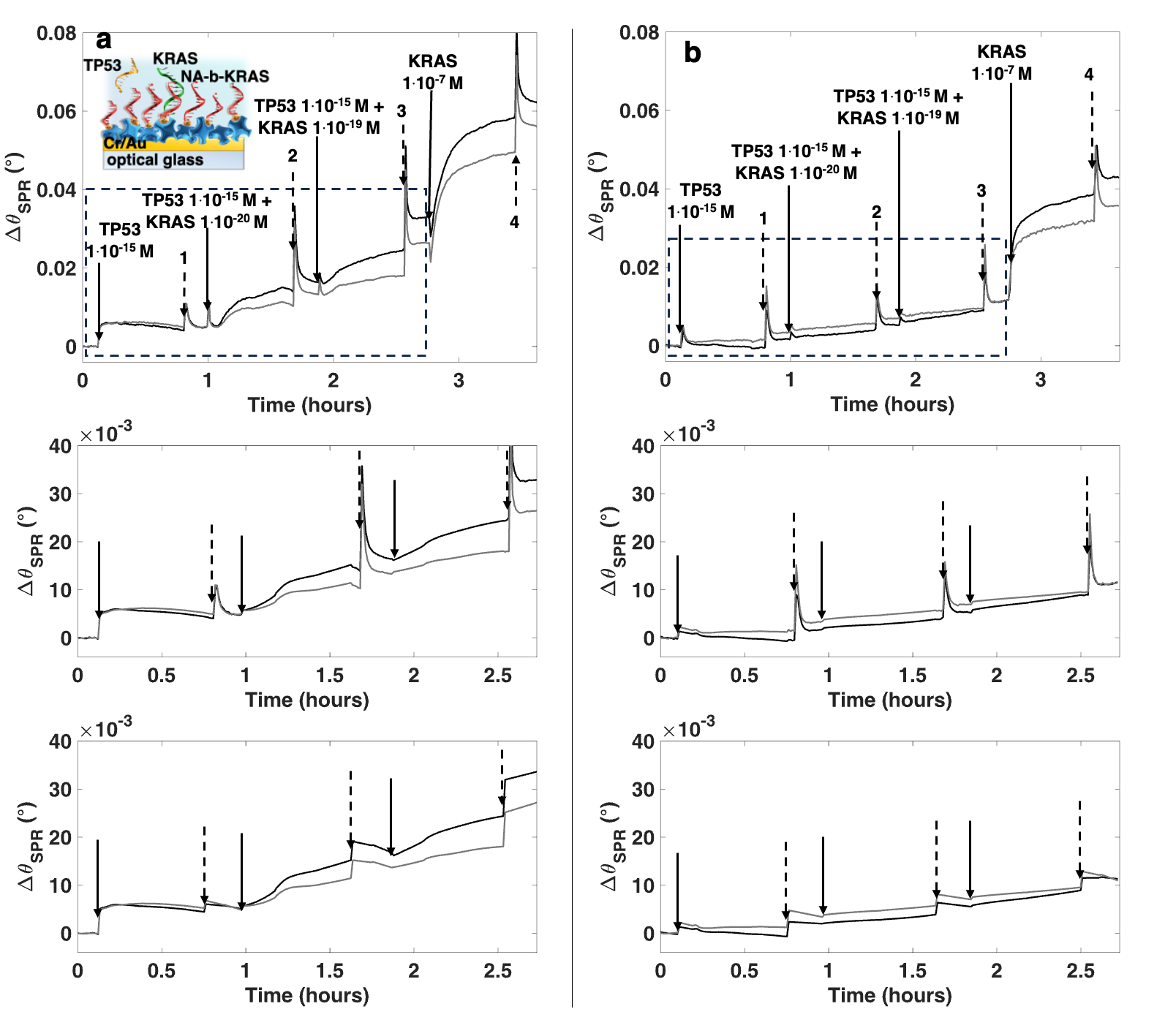
***

**Figure S18. Sensing raw and smoothed data on a NA-b-KRAS probe biolayer conditioned at pH 6 -**  First **(a)**, second **(b),** and third **(c)** SPR sensogram replicate of the NA-b-KRAS biolayer conditioned at pH 6. The reference is the θ_SPR_ of the biofunctionalized slide kept in HEPES@pH7.4 which is the buffer solution used during the physisorption and all throughout the experiments. Each trace is acquired in duplicates by sampling the 0.4 cm^2^ wide slide in two points set 3 mm apart. The first solid arrow indicates the injection (0.1 mL) of a 10-^15^M TP53 HEPES@pH7.4 solution, with the non-binding TP53 serving as interferent. After 40 minutes of incubation a first dashed arrow indicates a washing step (0.1 mL of HEPES@pH7.4), followed, after 10 minutes (second solid arrow) by the injection of a KRAS 10^-20^M in TP53 10-^15^M HEPES@pH7.4 solution (1±1 protein in the 0.1 mL injected volume). A second dashed arrow indicates a further washing step (0.1 mL of HEPES@pH7.4) followed by the injection (third solid arrow) of a KRAS 10^-19^M in a TP53 10-^15^M HEPES@pH7.4 solution (6±2 proteins in the 0.1 mL injected volume). After a third washing step the injection of a KRAS 10^-7^M in HEPES@pH7.4 is injected and finally washed. In the upper panels the whole measured curve is shown, while in the middle panels the sensogram region up to the 10^-19^M analyte injection is zoomed-in. A light third-degree smoothing routine is performed on the whole curve except the regions where the injection of a new batch occurs. Here a one-degree smoothing routine is used.

***
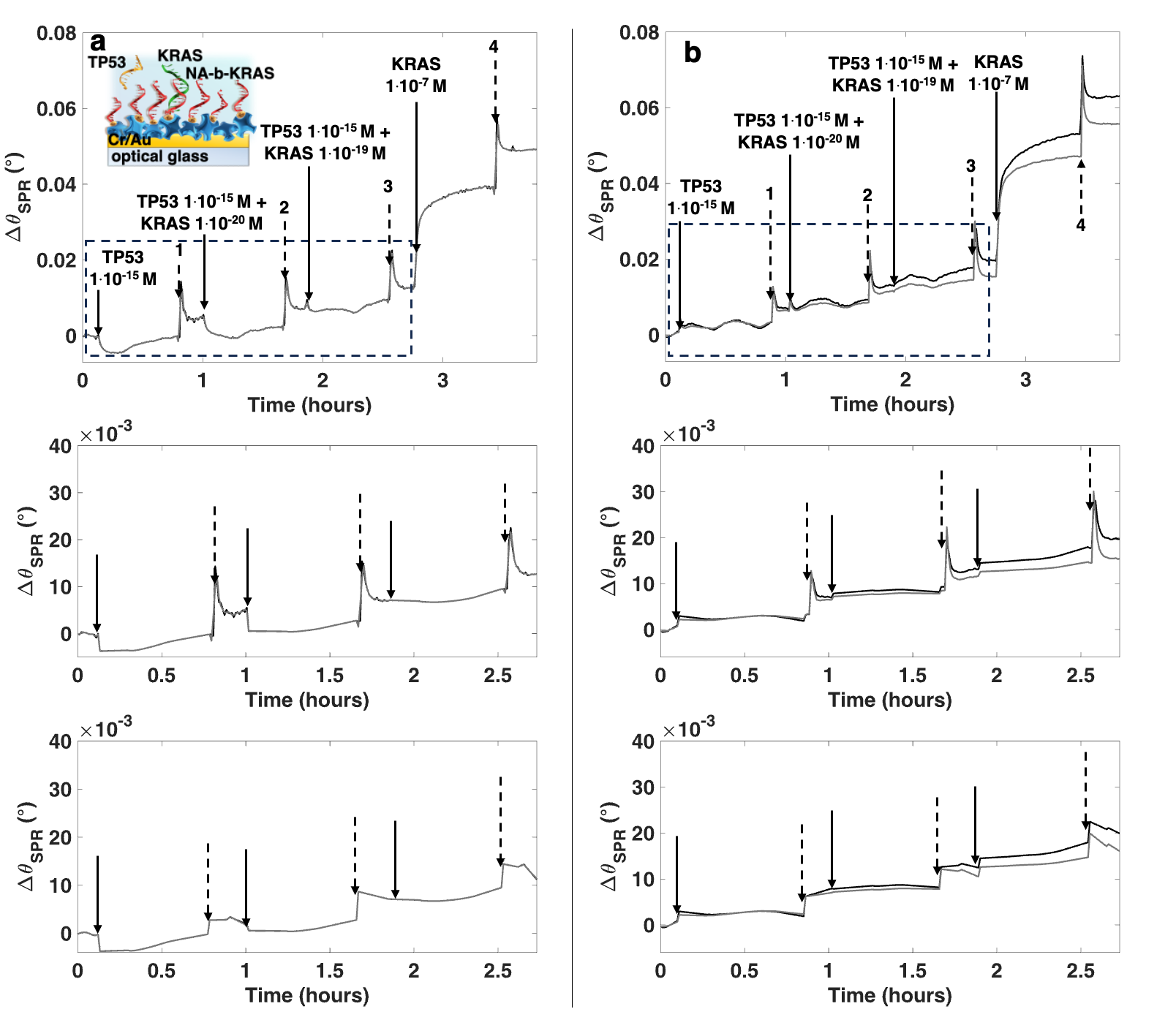
***

**Figure S19. Sensing raw and smoothed data on a NA-b-KRAS probe biolayer conditioned at pH 8 -** First **(a)**, second **(b),** and third **(c)** SPR sensogram replicate of the NA-b-KRAS biolayer conditioned at pH 8. The reference is the θ_SPR_ of the biofunctionalized slide kept in HEPES@pH7.4 which is the buffer solution used during the physisorption and all throughout the experiments. Each trace is acquired in duplicates by sampling the 0.4 cm^2^ wide slide in two points set 3 mm apart. The first solid arrow indicates the injection (0.1 mL) of a 10-^15^M TP53 HEPES@pH7.4 solution, with the non-binding TP53 serving as interferent. After 40 minutes of incubation a first dashed arrow indicates a washing step (0.1 mL of HEPES@pH7.4), followed, after 10 minutes (second solid arrow) by the injection of a KRAS 10^-20^M in TP53 10^-15^M HEPES@pH7.4 solution (1±1 protein in the 0.1 mL injected volume). A second dashed arrow indicates a further washing step (0.1 mL of HEPES@pH7.4) followed by the injection (third solid arrow) of a KRAS 10^-19^M in a TP53 10-^15^M HEPES@pH7.4 solution (6±2 proteins in the 0.1 mL injected volume). After a third washing step the injection of a KRAS 10^-7^M in HEPES@pH7.4 is injected and finally washed. In the upper panels the whole measured curve is shown, while in the middle panels the sensogram region up to the 10^-19^M analyte injection is zoomed-in. A light third-degree smoothing routine is performed on the whole curve except the regions where the injection of a new batch occurs. Here a one-degree smoothing routine is used.

In **Figure S20** the temperature oscillations during the SPR sensing experiments for all the systems assessed are shown. This is the temperature measured *in-situ* and *operando* in the SPR cell during the experiments shown in **Figure S7-S19**. The temperature shift (ΔT) is the temperature variation at a given time of the sensing experiment compared to the baseline. The ΔT curves are given for all the sensogram measured at each stage of the sensing protocol, namely on the non-conditioned layer (black circles) and on the layer conditioned at both pH6 (red circles) and pH8 (blue circles). The temperature oscillations are within the SPR instrument specifications, specifically within ± 0.1 °C, and remain consistent throughout all stages of sensing. This clearly proves that any temperature variation - assuming such minor oscillations could affect the properties of the capturing layer - would impact both the negative control and the sensing experiment equally. Therefore, they fall within the noise of the sensing angular shift and hence are not relevant towards the single/few-molecule sensing signal.

**
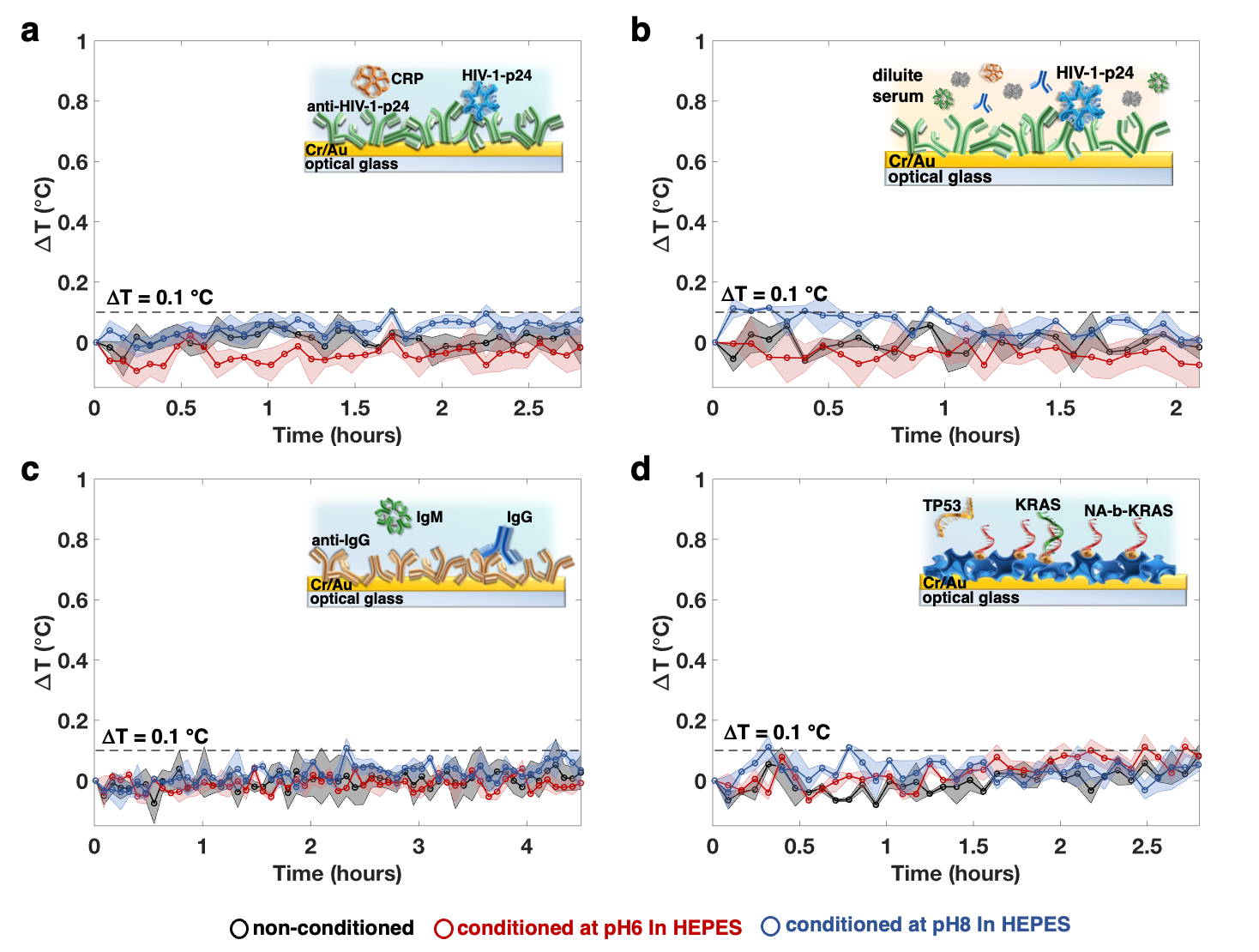
**

**Figure S20. Control of the temperature during all the SPR sensing experiments -** Temperature shifts (ΔT) relative to the baseline measured during SPR sensing experiments for the detection of **(a)** HIV-1-p24 in HEPES@pH7.4 buffer, **(b)** HIV-1-p24 in diluted human serum, **(c)** IgG in HEPES@pH7.4 and **(d)** KRAS in HEPES@pH7.4. Black-circles represent the non-conditioned biolayers, red-circles and blue-circles correspond to the biolayers conditioned at pH6 and pH8 respectively. The colored shadings represent one standard deviation, while the dashed black-lines corresponds to the instrumental error.

*Surface coverage of the biological layer*

This table summarizes the surface density of the biological systems deposited as evaluated by the SPR data with **Eq S6**. Both the capturing and probes layers as well as the analyte in the double-layer sensing regimes are shown.

Table S3: Summary of the coverages of all the layers investigated (both physisorbed recognition biolayer and the layers of antigens or KRAS in the double-layer regime), computed from the measured SPR angle shifts using Eq. S6

| biological layer | deposition conditions in HEPES@pH7.4 | molecules  surface density  (molecules/cm^2^) | figure(s) where data are shown |
| --- | --- | --- | --- |
| **anti-HIV-1-p24** | *physisorption from* 50 μg/mL of anti-HIV-1-p24 for 2 hours | (1.3 ± 0.1) ·10^12^ | **Figure S2b** |
| **anti-IgG** | *physisorption from* 50 μg/mL of anti-IgG for 2 hours | (7.1 ± 0.4) ·10^11^ | **Figure S3b** |
| **NA-b-KRAS** | *physisorption from* 50 μg/mL of NA-b-KRAS for 2 hours | (4.2 ± 0.1) ·10^12^ | **Figure S4b** |
| **HIV-1-p24 (on non-conditioned anti-HIV-1-p24)** | *deposition from*  *HIV-1-p24 10^-7^M*  *(double-layer regime)* | (1.5±0.6) ·10^12^ | **Figure S7** |
| **HIV-1-p24 (on anti-HIV-1-p24conditioned at pH 6)** | *deposition from*  *HIV-1-p24 10^-7^M*  *(double-layer regime)* | (1.1 ± 0.2) ·10^12^ | **Figure S8** |
| **HIV-1-p24 (on anti-HIV-1-p24 conditioned at pH 8)** | *deposition from*  *HIV-1-p24 10^-7^M*  *(double-layer regime)* | (1.4 ± 0.1) ·10^12^ | **Figure S9** |
| **IgG (on non-conditioned**  **anti-IgG)** | *deposition from IgG 10^-7^M (double-layer regime)* | (4.6 ± 0.7) ·10^11^ | **Figure S13** |
| **IgG (on anti-IgG**  **conditioned at pH 6)** | *deposition from IgG 10^-7^M (double-layer regime)* | (6.2 ± 0.9) ·10^11^ | **Figure S14** |
| **IgG (on anti-IgG**  **conditioned at pH 8)** | *deposition from IgG 10^-7^M (double-layer regime)* | (5 ± 1) ·10^11^ | **Figure S15** |
| **KRAS (on**  **non-conditioned NA-b-KRAS)** | *deposition from KRAS 10^-7^M (double-layer regime)* | (1.9 ± 0.1) ·10^12^ | **Figure S17** |
| **KRAS (on NA-b-KRAS conditioned at pH 6)** | *deposition from KRAS 10^-7^M (double-layer regime)* | (2.4 ± 0.6) ·10^12^ | **Figure S18** |
| **KRAS (on NA-b-KRAS conditioned at pH 8)** | *deposition from KRAS 10^-7^M (double-layer regime)* | (2.7 ± 0.3) ·10^12^ | **Figure S19** |

***SN6. Comparison between the single/few-molecules and the double-layer regimes***

In **Figure S21** the sensing response of a non-conditioned anti-IgG layer to the IgG affinity ligand in the very broad 10^-21^ - 10^-7^ M concentration range is shown as black squares, while red and blue circles are relevant to anti-IgG biolayers conditioned at pH 6 and pH 8, respectively. It is confirmed that, while the non-conditioned biolayer shows an appreciable signal only beyond nM concentrations, single or few molecules are detected when the biolayer is conditioned at pH 6 or pH 8. Saturation is measured around 10^-7^ M that corresponds to an IgG surface density of (4.6 ± 0.7)∙10^11^ molecules/cm^2^, namely as anti-IgG coverage is (7.1 ± 0.4)∙10^11^ molecules//cm^2^, approximately one IgG binds every two anti-IgGs. HIV-1-p24 and KRAS at 10^-7^ M concentration bind to non-conditioned biological recognition layer with surface coverages of (1.5 ± 0.6)∙10^12^ molecules/cm^2^ and (1.9 ± 0.1)∙10^12^ molecules/cm^2^ which are, also, comparable to those of the biolayer underneath. Thus, proving that at ligand concentrations of at least in the nM range, the SPR signal change, associate with a Δn shift, is ascribable to the formation of a double-layer structure. The data in **Table S3** further show how the pH conditioning enabling the sensing at extremely low concentration, does not inhibit the binding ability at much higher ligand concentrations for all the biorecognition layers investigated. Comparable or even higher surface coverages of antigens or nucleic acids are, in fact, measured on the pH-conditioned recognition elements layers. Meaning that the pH conditioning protocol does not fully denature the capturing antibodies or the probes in the biolayer. However, a much larger associated error bar is seen on the responses from the conditioned biolayers, evidencing a lower reproducibility of the binding capabilities at high concentrations. Conversely, the Δθ_SPR_ shifts measured in the 10^-20^ - 10^-16^ concentration range are, due to the severe mass diffusion-limited process, necessarily to be ascribed to a change in the refractive index of the biological recognition layers upon single/few-molecules sensing. The two completely different sensing regimes are addressed as “single/few-molecules” and “double-layer” as evidenced in **Figure S21**.


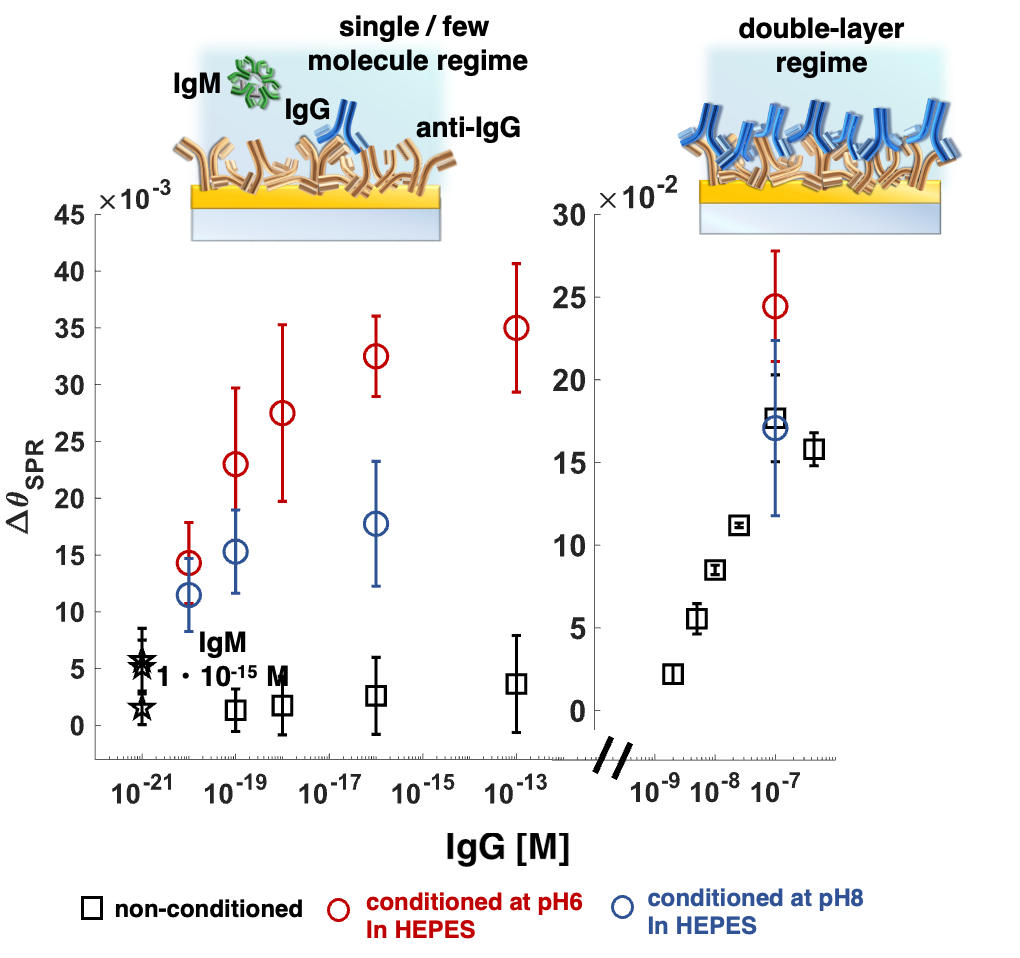


**Figure S21**. **Surface Plasmon Resonance single/few-molecules and double-layer regimes -** IgG sensing (10^-21^-10^-7^ M range) Δθ_SPR_ data-points measured in HEPES@pH7.4 at a non-conditioned (black squares), conditioned at pH 6 (red circles) and conditioned at pH 8 (blue circles) anti-IgG capturing layers. The vertical axis scale is magnified in the 10^-21^-10^-13^ M range as Δθ_SPR_ is about one order of magnitude lower than at higher concentrations. The sensing protocol involves the exposure of the anti-IgG coated SPR slide to 0.1 mL of HEPES@pH7.4 to set the θ_SPR_ reference angle. Afterword, a 0.1 mL aliquot of an IgM 10^-15^ M solution in HEPES@pH7.4 is injected and the relevant Δθ_SPR_ shift (hollow star symbol) is measured. Then, 0.1 mL aliquots of IgG solution in HEPES@pH7.4 at different concentrations are injected and assessed. All the data points panels are averaged over at least 3 replicates, and the error bars are taken as one standard deviation.

***SN7. An ionic strength change does not enable single molecule detections***

An important aspect is to decouple the role of the pH shift from that of an ionic strength change during the biolayer conditioning. To this end, variants in the conditioning protocols encompassing both i_s_ and/or pH shifts, are scrutinized. In **Figure S22** the Δθ_SPR_ data measured for the sensing of IgG at 1·10^-20^M , 1·10^-19^ M, 1·10^-16^ M concentrations along with the response to the non-binding of IgM at 1·10^-15^ M (star symbol) from which the LOD level is estimated.


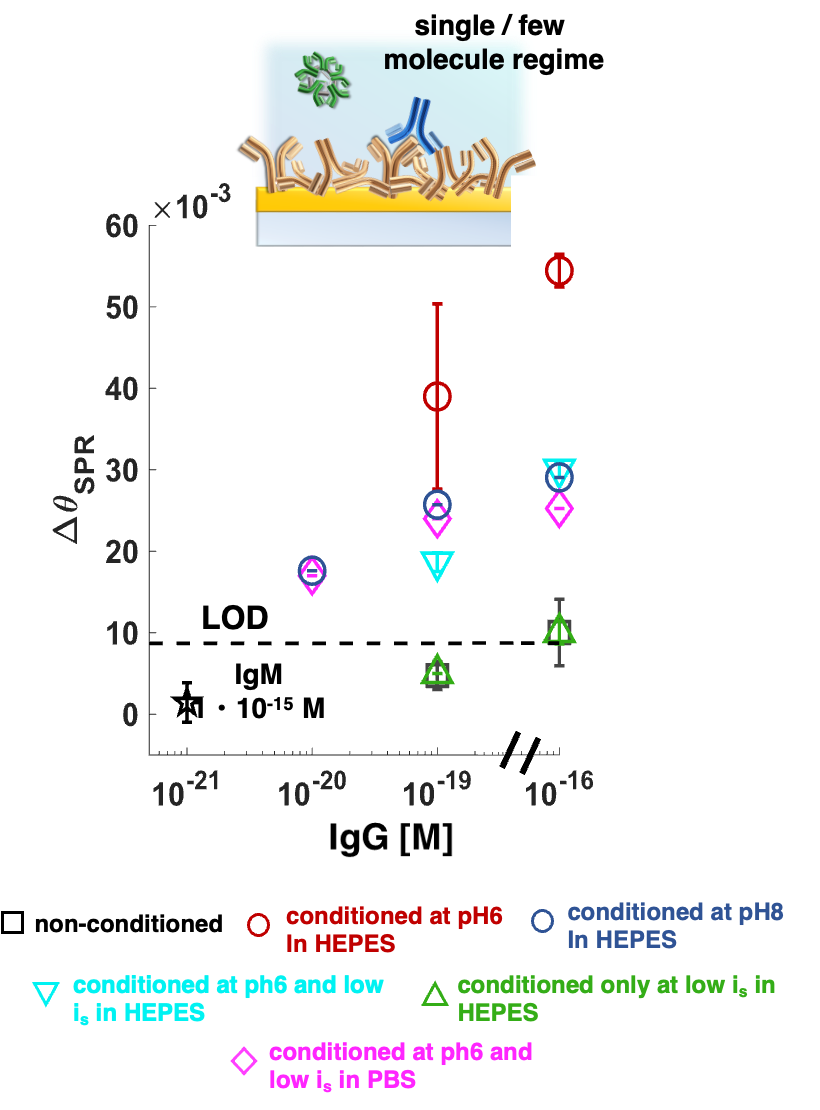


**Figure S22** **IgG sensing at anti-IgG biolayers conditioned for 30 minutes according to different protocols** **-** Black squares are relevant to a non-conditioned anti-IgG layers; red and blue circles are measured on the anti-IgG layers conditioned at pH 6 and pH 8 respectively; cyan down-triangles are measured for an anti-IgG conditioned in an HEPES buffer at pH=6 and i_s_=5 mM (HEPES@pH6/i_s_-low) hence exposed to a decrease in pH (from 7.4 to 6), and in i_s_ (from 150mM to 5 mM ); green up-triangles represent the biolayer conditioned into an HEPES buffer at pH=7.4 and i_s_=5mM (HEPES@pH7.4/i_s_-low) hence only to a decrease in i_s_ (from 150mM to 5mM); the magenta diamonds are relevant to an anti-IgG biolayer deposited from a phosphate buffer saline (PBS) solution (pH=7.4, i_s_=163 mM) and rinsed in HPLC-grade deionized water (pH~ 5.5 and i_s_~ 5 μM). The Δθ_SPR_ data are measured, for all the anti-IgG biolayers, according to the IgG (10^-20^M, 10^-19^M, and 10^-16^M in HEPES@pH7.4 or PBS) sensing protocol described in **Figure S19** along with the negative control experiment (IgM 10^-15^M, 17 replicates) indicated also as a black star. All the data points in both panels are averaged over at least 3 replicates, and the error bars are taken as one standard deviation.

The pH-conditioning processes are accomplished according to the protocol. The red and the blue circles are Δθ_SPR_ replicates for the capturing layer conditioned in HEPES@pH6 and HEPE@pH8 at fixed ionic strength. The green up-triangles represent the anti-IgG biolayer exposed to a sole decrease in i_s_ going from 150 mM to 5 mM in HEPES at pH 7.4. In this case the refractive index shift upon single/few molecules binding is comparable to that of a non-conditioned layer, proving the non-effectiveness of this treatment. The cyan down-triangles are data-points measured on an anti-IgG exposed to both a decrease in pH, from 7.4 to 6, and in i_s_ from 150 mM to 5 mM in HEPES. A similar conditioning protocol (magenta diamonds) involves an anti-IgG biolayer deposited from a phosphate buffer saline (PBS) solution (pH = 7.4, i_s_ = 163 mM) and conditioned in deionized water (pH ~ 5.5 *ca.* and i_s_ ~ 5 μM). Despite the change in buffer composition, these two protocols result in comparable Δθ_SPR_ values falling all beyond the LOD level. This further prove the generality of the pH-conditioning step in enabling single/few-molecules sensing effect to a broaden extent, namely in different buffer solutions. In summary, the data in **Figure S22** show that the protocols involving both a pH and an i_s_ shifts leads to signals comparable to those measured on the recognition layer conditioned at pH 8 and they are all lower than the highest signal detected when the anti-IgG is conditioned shifting the sole pH from 7.4 to 6. On the other hand, if no pH shift is in place no significant Δθ_SPR_ response is recorded in the whole inspected range, thus proving the single-molecule sensing enabling nature of the pH-conditioning, particularly when the pH shift leads to a higher proton concentration in the conditioning buffer solution.

A more systematic evaluation involving an experimental design study leads to the same conclusions, namely, it is the pH shift that enable the single/few-molecules sensing at the millimeter large interface. The multiple condition steps protocol used for IgG detection on the anti-IgG as illustrated in **Figures S14 - S16,** is employed to investigate the impact of both ionic strength (i_s_) and pH conditioning in enabling single/few-molecule sensing at a millimeter wide SPR interface. It is further demonstrated that this effect is observable not only when using an HEPES buffer but also when engaging a PBS solution. The study encompasses different variants of the multiple condition steps protocol involving pH and/or i_s_ change. For all these protocols, the first step sees the deposited pristine anti-IgG biolayer exposed to the non-binding IgM (1·10^-15^ M) in 0.1 mL of HEPES@pH7.4 (i_s_ = 150 mM). Moreover, the θ_SPR_ baseline (against which the Δθ_SPR_ shifts are measured) is measured while the anti-IgG is at rest in HEPES@pH7.4. The protocols used are the followings:

- ***Protocol A (no change in pH or i_s_) - non conditioned biolayer***: after 30 minutes of stabilization of the θ_SPR_ baseline in HEPES@pH7.4 (i_s_ = 150 mM), a new 0.1 mL batch of HEPES@pH7.4, entailing non pH or i_s_ change, is injected in the SPR cell, and let to rest on the biolayer for 40 minutes. Then, 0.1 mL of IgG 1·10^-19^M, 1·10^-16^M and 1·10^-7^M in HEPES@pH7.4 aliquots are sequentially injected and let to rest in the SPR cell for 40 minutes. Each step is followed by a 10 minutes rinsing in HEPES@pH7.4.

- ***Protocol B (change in pH at fixed i_s_) - biolayer conditioned at pH 6***: after 30 minutes of stabilization of the θ_SPR_ baseline in HEPES@pH7.4, a conditioning step in HEPES@pH6 (i_s_ = 150 mM) is carried out in the SPR cell for 30 minutes, followed by a rinsing in HEPES@pH7.4 for 10 minutes. Then, 0.1 mL of IgG 1·10^-19^ M, 1·10^-16^ M and 1·10^-7^ M in HEPES@pH7.4 aliquots are sequentially injected and let to rest in the SPR cell for 40 minutes. Each step is followed by a 10 minutes rinsing in HEPES@pH7.4 and a further conditioning for 30 minutes.

- ***Protocol C (change in pH and i_s_) - biolayer conditioned at pH 6 and i_s_ = 5mM***: after 30 minutes of stabilization of the θ_SPR_ baseline, a conditioning step in HEPES@pH6/i_s_-low (pH = 6, i_s_ = 5 mM) is carried out in the SPR cell for 30 minutes, followed by a rinsing in HEPES@pH7.4 for 10 minutes. Then, 0.1 mL of IgG 1·10^-19^ M, 1·10^-16^ M and 1·10^-7^ M in HEPES@pH7.4 aliquots are sequentially injected and let to rest in the SPR cell for 40 minutes. Each step is followed by a 10 minutes rinsing in HEPES@pH7.4 and a further conditioning for 30 minutes.

- ***Protocol D (change in i_s_ at fixed pH) - biolayer conditioned at pH 7.4 and i_s_ = 5 mM***: after 30 minutes of stabilization of the θ_SPR_ baseline, a conditioning step in HEPES@pH7.4/i_s_-low (pH = 7.4, i_s_ = 5 mM) is carried out for 30 minutes in the SPR cell, followed by a rinsing in HEPES@pH7.4 for 10 minutes. Then, 0.1 mL of IgG 1·10^-19^ M, 1·10^-16^ M and 1·10^-7^ M in HEPES@pH7.4 aliquots are sequentially injected and let to rest in the SPR cell for 40 minutes. Each step is followed by a 10 minutes rinsing in HEPES@pH7.4 and a further conditioning for 30 minutes. The LOD level is calculated from the negative control experiment as the average over 15 replicates plus 3 times the standard deviation.

To systematically investigate the role of pH and i_s_ shift in the single/few-molecules sensing, an experimental design is set up according to the scheme given in **Figure S23**. The study involves results from **Protocols A, B, C, and D** entailing only HEPES-based buffers. The ∆θ_SPR_ shifts measured, are listed in **Table S4**. The experimental design is carried out according to a 2^2^ full factorial design^[10,51–53]^ based on the decrease of ionic strength (∆i_s_) from 150 mM to 5 mM and the decrease of the pH (∆pH) from 7.4 to 6. Following a two-level design, the two quantitative variables have been encoded as X_1_ and X_2_, representing the ∆i_s_ and ∆pH respectively, settled at two levels, namely -1 (no variation) and +1 (full variation). The 2^2^ factorial design explores the corners of a square where both variables to change simultaneously. The output of the experiment performed at the corners of the experimental domain, marked by the black circles in **Figure S24**, are used to feed the model. In addition, two experiments have been performed at the center point, shown as a gray circle, to assess the model prediction capability.


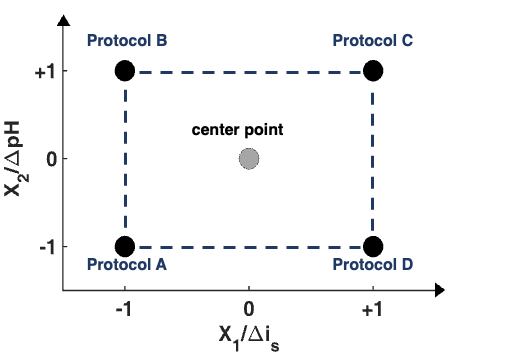


**Figure S23. Two-level experimental design -** Graphical representation of a 2^2^ factorial design entailing protocols A-D.

Table S4: ∆θ_SPR_ shifts, with an associated error of 1⋅10^-3^ (°), of IgG sensing at 1·10^-19^ M, 1·10^-16^ M, and 1·10^-7^ M under different i_s_ and pH change conditioning as described in protocols A-D. The ∆θ_SPR_ shifts are estimated against the baseline measured on the pristine biolayer in HEPES@7.4. The X_1_ and X_2_ encoded values representing the ∆i_s_ and ∆pH respectively.

|  |  |  |  |  |  | **∆θ_SPR_ shift for**  **IgG as 10^-3^ (°)** | | | **θ_SPR_ for**  **IgM as 10^-3^ (°)** |
| --- | --- | --- | --- | --- | --- | --- | --- | --- | --- |
| **# replicates** | **protocol** | **i_s_ (10^-3^M)** | **pH** | **X_1_** | **X_2_** | **10^-19^M** | **10^-16^M** | **10^-7^M** | **10^-15^M** |
| 1 | **A** | 150 | 7.4 | -1 | -1 | 0.003 | 0.006 | 0.160 | 0.002 |
| 2 |  | 150 | 7.4 | -1 | -1 | 0.004 | 0.007 | 0.160 | 0.003 |
| 3 |  | 150 | 7.4 | -1 | -1 | 0.005 | 0.013 | 0.160 | 0.003 |
| 4 |  | 150 | 7.4 | -1 | -1 | 0.007 | 0.014 | 0.160 | 0.005 |
| 1 | **B** | 150 | 6.0 | -1 | +1 | 0.052 | 0.075 | 0.246 | 0.001 |
| 2 |  | 150 | 6.0 | -1 | +1 | 0.034 | 0.053 | 0.143 | 0.001 |
| 3 |  | 150 | 6.0 | -1 | +1 | 0.031 | 0.035 | 0.104 | 0.001 |
| 1 | **C** | 5 | 6.0 | +1 | +1 | 0.015 | 0.015 | 0.115 | -0.001 |
| 2 |  | 5 | 6.0 | +1 | +1 | 0.013 | 0.011 | 0.147 | 0.003 |
| 3 |  | 5 | 6.0 | +1 | +1 | 0.013 | 0.016 | 0.141 | 0.001 |
| 4 |  | 5 | 6.0 | +1 | +1 | 0.011 | 0.012 | 0.092 | -0.007 |
| 5 |  | 5 | 6.0 | +1 | +1 | 0.018 | 0.020 | 0.040 | -0.007 |
| 6 |  | 5 | 6.0 | +1 | +1 | 0.018 | 0.021 | 0.080 | 0.002 |
| 7 |  | 5 | 6.0 | +1 | +1 | 0.014 | 0.019 | 0.060 | -0.008 |
| 1 | **C** | 5 | 7.4 | +1 | -1 | 0.020 | 0.020 | 0.100 | 0.006 |
| 2 |  | 5 | 7.4 | +1 | -1 | 0.010 | 0.010 | 0.160 | 0.009 |
| 3 |  | 5 | 7.4 | +1 | -1 | 0.005 | 0.013 | 0.134 | 0.002 |

The analysis based on multivariate linear regression provides the model, correlating the ∆θ_SPR_ at 10^-19^ M with X_1_ and X_2_, given in **Eq. S7**:

$\Delta\theta_{SPR}@{10}^{-19}M =0.017-0.005 X_{1}^{(**)}+0.010 X_{2}^{(***)}-0.007 X_{1}X_{2}^{(***)}$ (**S7**)

where the significance level is indicated according to the usual convention: * = p < 0.05, ** = p < 0.01, *** = p < 0.001. Those p-values define the confidence interval of the Student’s t-test used to evaluate the significance of each coefficient in the regression model. In this single-molecule regime the selected design, computed with 15 degrees of freedom, gives a maximum leverage of 0.33, namely response can be predicted with better precision than the experimental data collected under the same conditions^[51–53]^. Moreover, the experimental value of the ∆θ_SPR_ registered at the center point (0.017 ± 0.005) (°) is not significantly different from the predicted value 0.017(°). Thus, the model is validated and accepted in the whole experimental domain. The data in **Figure S24a** reducing the conditioning from 7.4 to 6 at fixed physiological i_s_, leads to an increase in ∆θ_SPR_ of one order of magnitude. On the other hand, a rinsing buffer with a lower ionic strength has a limited impact on the ∆θ_SPR_. The coefficient for each term represents the change in the mean response associated with an increase of one coded unit in that term, while the other terms are kept constant. By changing the ionic strength of the rinsing buffer from 150 mM to 5mM, the SPR angle shift variation falls below the LOD, while the pH change results in a ∆θ_SPR_ higher than the LOD, being hence significant. The isoresponse contour plot, reported in **Figure S24b,** provides information about the interactions among the two variables X_1_ and X_2_. The geometrical shape of a linear model without interactions is a plane, leading to isoresponse lines that are parallel, while if relevant interactions are present, the contour plot shows a distorted plane with the isoresponse lines that are not parallel. This implies that the effect of a lower ionic strength of the rinsing buffer becomes more relevant only at higher pH differences. In comparison, at much lower pH values of the rinsing buffer, the ionic strength has little to no effect. Moreover, **Figure S24a** confirms that the optimal condition to get an SPR response at very low IgG concentrations (in the explored experimental domain) corresponds to lowering the pH of the rinsing buffer. On the other hand, a change in the ionic strength of the rinsing buffer is not significantly improving the SPR angle shift.

For the ∆θ_SPR_ registered with sensing at 1·10^-16^ M, the analysis based on multivariate linear regression provided the following model correlating and the two coded variables as in **Eq. S8** below:

$\Delta\theta_{SPR}@{10}^{-16}M=0.024-0.009 X_{1}^{(***)}+0.012 X_{2}^{(***)}-0.010 X_{1}X_{2}^{(***)}$ (**S8**).

This can be assimilated to a few-molecules regime as only 10^4^ molecules are found in 0.1 mL. Indeed, **Eq. S8** is in line with the main features of the model computed for the sensing at 1·10^-19^ M. The selected design, computed with 15 degrees of freedom, also produced a maximum leverage of 0.33. Moreover, the experimental value of SPR angle shift registered at the center point is (0.020 ± 0.015) (°), which is not significantly different from the predicted value 0.024 (°). Thus, the model is validated and accepted in the whole experimental domain. The coefficient of the model, reported in **Figure S24c**, are all significant terms, and even in this case, the linear term of X_2_ holds an absolute value larger than the other ones. The isoresponse contour plot, reported in **Figure S24d**, shows also in this case that the preferred (as well as providing the best results) condition to enhance the SPR angle shift in the explored experimental domain entails the lowering pH of the rinsing buffer, while reducing the ionic strength of the rinsing buffer does not produce an improvement in the SPR angle shift.

The analysis performed on the ∆θ_SPR_ registered upon exposure to 1·10^-7^ M of IgG returns the model given by **Eq. S9** below:

$\Delta\theta_{SPR}@{10}^{-7}M=0.135-0.02 X_{1}^{\left( * \right)}-0.007 X_{2}-0.009 X_{1}X_{2}$ (**S9**).

This is the double-layer regime where a layer of IgG is built on top of the anti-IgG capturing layer. In **Figure S19e** the X_1_, associated with the change in ionic strength is the only significant coefficient in this case, implying that the dependence on the ionic strength of the rinsing buffer is the only aspect that matters. Also in this case, the experimental value of SPR angle shift Δθ_SPR_ registered at the center point is (0.118 ± 0.040)(°), which is not significantly different from the predicted value 0.135 (°). Remarkably, in this case, reducing the ionic strength of the rinsing buffer from 150 mM to 5mM produces a lowering of the SPR angle shift of about 15 %, while the effect of the pH becomes negligible. The very different models that are entailed at low concentrations (1·10^-19^ M and 1·10^-16^ M) and at 1·10^-7^ M, fully account for the need to consider two completely different regimes in the SPR IgG assay at a capturing anti-IgG layer, namely: a “single/few-molecules regime” where the change of the dielectric function involves an electrostatic/dielectric rearrangement in the anti-IgG biolayer and a “double-layer regime”, where the dielectric function is modified by the building of a new layer on top of the capturing anti-IgG one. Finally, the SPR angle shifts registered during the negative control experiments are negligible, as it can be seen in **Figure S24f**, independently from the measuring protocol, namely, neither the ionic strength nor the pH changes of the rinsing buffer, matter.


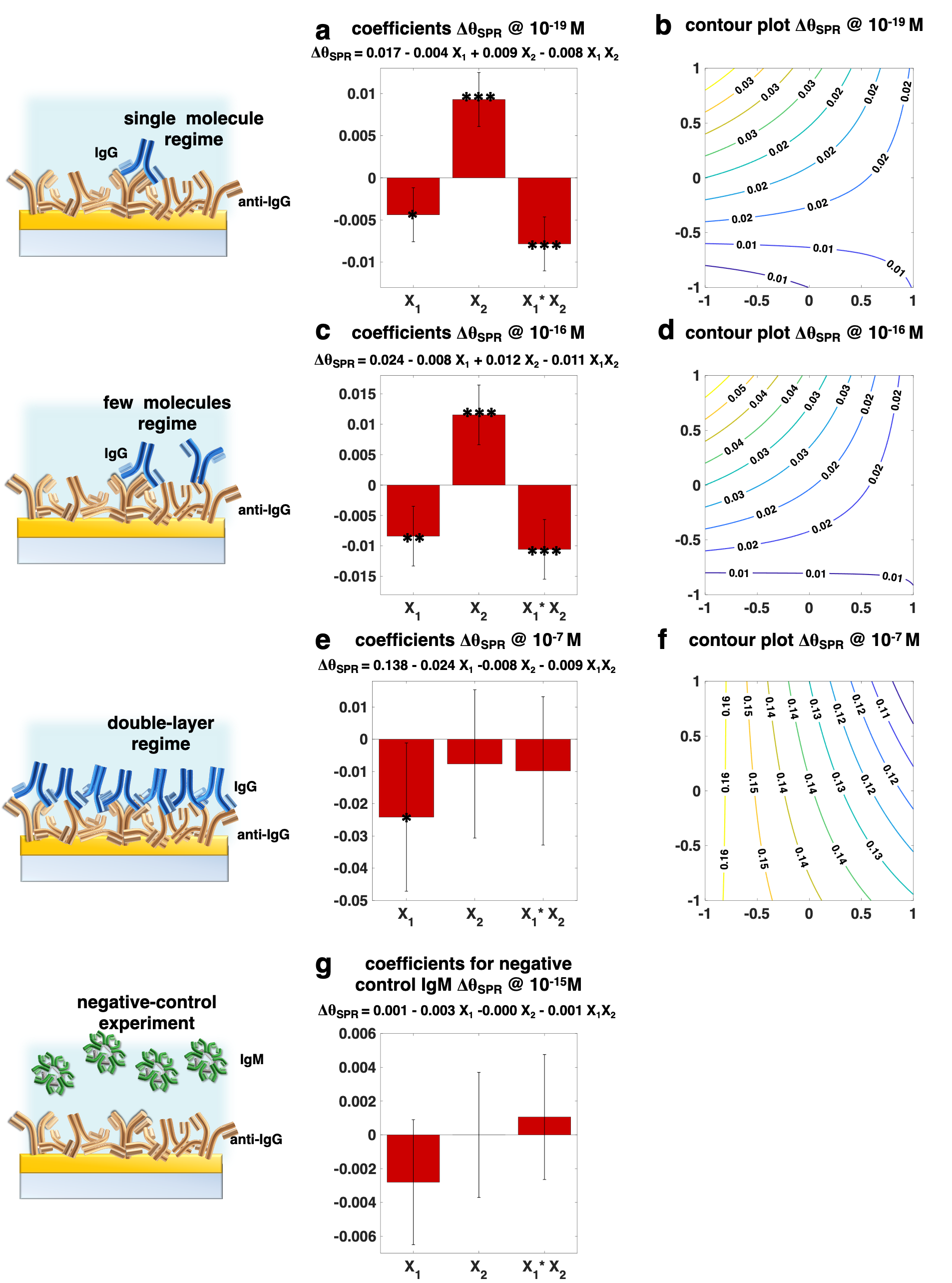


**Figure S24: Multivariate linear regression analysis -** Representation of the coefficients (**a, c**) and of the isoresponse contour plots (**b, d**) of a Δθ_SPR_ model in the 2^2^ full factorial design of the sensing with an anti-IgG biolayer at 10^-19^ M and at 10^-16^ M IgG concentrations. The coefficients are given also for the sensing at IgG 10^-7^ M (**e**) and for the exposure to IgM 10^-15^ M, this being the negative control experiment (**f**). Error bars are the confidence level at p=0.05, while the stars indicate the significance of the coefficients (*=p<0.05, **=p<0.01, ***=p<0.001).

***SN8.* EGOFETs single/few-molecules sensing**

The “Single-Molecule with a large Transistor (SiMoT)” bioelectronic qualitative sensing platform^[35,54–60]^ is based also on P3HT, EGOFETs. It can systematically detect proteins or nucleic acids selectively and reliably at an ultimate limit of 1·10^-20^ M, within an hour. It consists of a palmar reusable reader and a disposable cartridge integrated into two technologies tested in clinical settings. The 96-array can identify markers at the single-molecule level in the plasma of pancreatic cancer patients with a diagnostic sensitivity exceeding 96%^[56]^. The single-sensor technology detects a bacterium in olive sap^[61]^ or a virion in human saliva with an incidence of false negatives and false positives below 1%^[35]^. The performance level surpasses state-of-the-art methods and offers a faster, cost-effective alternative, creating new possibilities in point-of-care screening for early diagnosis.

In the present study a P3HT EGOFET whose sensing gate is biofunctionalized with an anti-HIV-1-p24 or a NA-b-KRAS biolayer. These layers are conditioned at pH 6 or pH 8 or not conditioned at all. The apparatus used is detailed in **Figure S25a** while the schematics of the anti-HIV-p24 or a NA-b-KRAS biolayers are detailed in **Figure S25b** and **Figure S25c**, respectively. The stability of the EGOFET channel is independently evaluated by driving the P3HT EGOFET with the reference gate (**Figure S25d**) and measuring V_T_ before starting and after completion of each sensing experiment. The gates are capacitively coupled to the p-type P3HT FET channel via the HEPES@pH7.4/i_s_-low electrolyte and the current flowing is modulated by the gate work function. The sensing protocol foresees the incubation of the gate in the HEPES@pH7.4 solutions to be assayed, then the gate is washed with the low salinity HEPES@pH7.4/i_s_-low and immersed into the transistor well to measure the transfer characteristic.


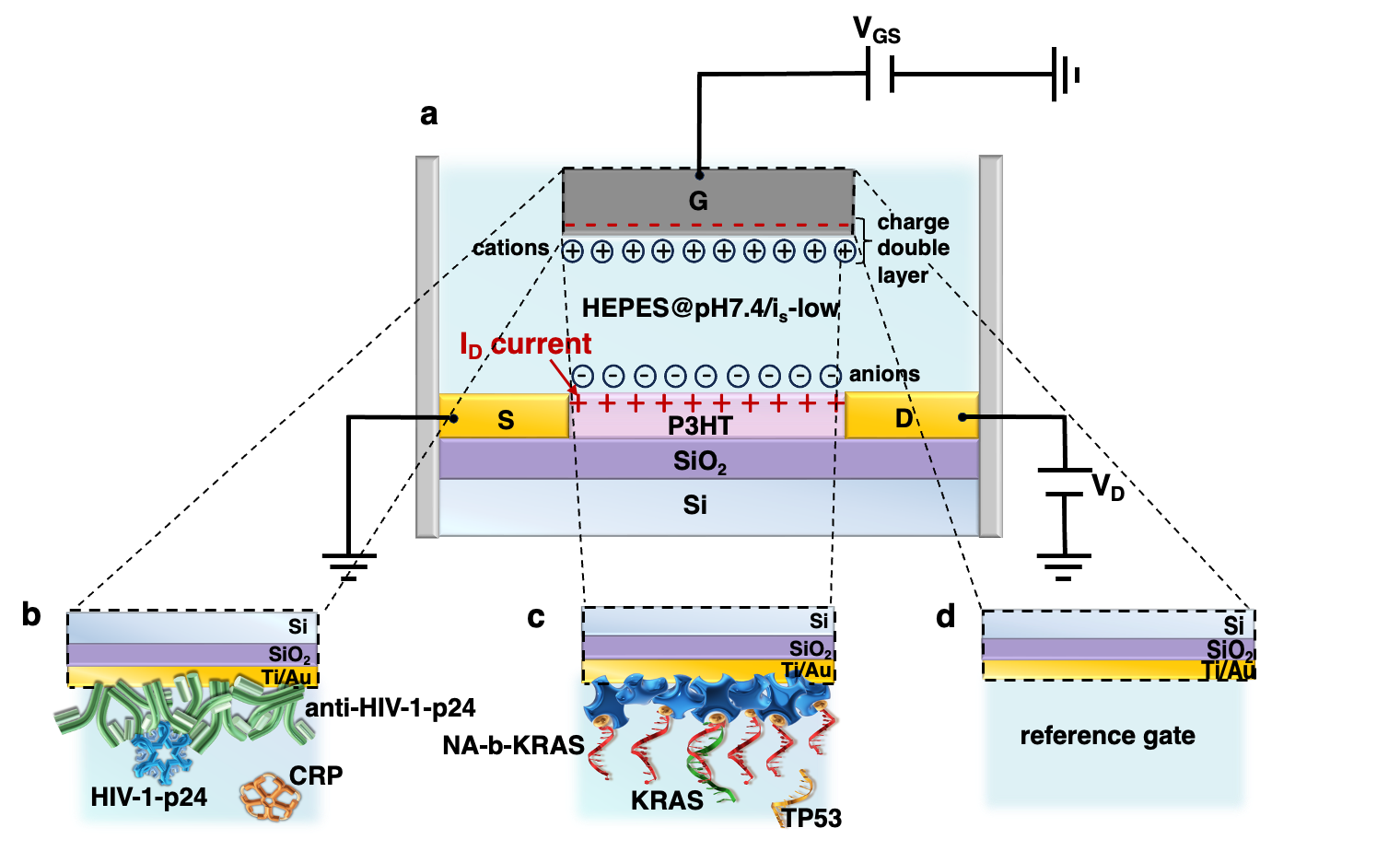


**Figure S25: EGOFET transistor configuration - (a)** Schematic representations of the EGOFET device comprising a biofunctionalized gate electrode (G) and a poly(3-hexylthiophene-2,5-diyl), P3HT, channel contacted through the source (S) and drain (D) electrodes. The electrolyte is a low salinity HEPES@pH7.4/i_s_-low (i_s_=5 mM); **(b)** the anti-HIV-p24 and **(c)** the NA-b-KRAS gate biolayers are also detailed; **(d)** the bare gold reference gate.

*Bioelectronic sensing of the HIV-1-p24*

**
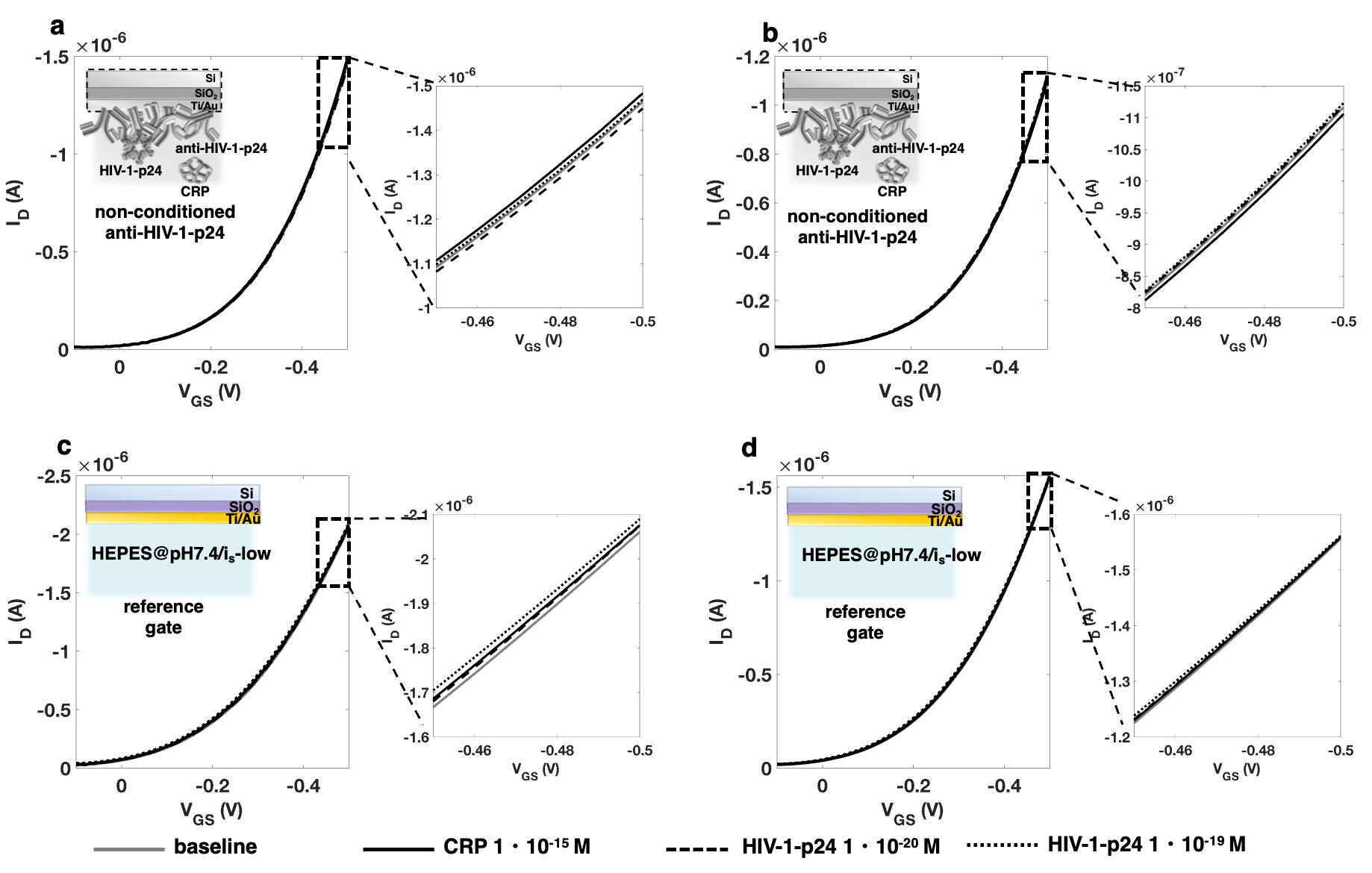
**

**Figure S26: EGOFET transfer characteristics row data for the HIV-1-p24 bioelectronic sensing at a non-conditioned anti-HIV-1-p24 functionalized gate –** In panels **(a)** and **(b)** the two replicates of the I_D_ *vs.* V_GS_ transfer characteristics (V_GS_ swiped from 0V to -0.5V while V_D_ is kept fixed at -0.4V) biased by a non-conditioned anti-HIV-1-p24 functionalized gate, are displayed. The curves are measured in HEPES@7.4/i_s_-low (i_s_ = 5 mM) while all the assayed solutions are in HEPES@pH7.4. For each sample, four curves are measured, namely: *-i)* the baseline I_0_ (gate incubated in HEPES@pH7.4); *-ii)* I_D_ of the negative control experiment (gate incubated in CRP 1·10^-15^M in HEPES@pH7.4); *-iii)* I_D_ of the single-molecule sensing (gate incubated in HIV-1-p24 1·10^-20^M and CRP 10^-15^M in HEPES@pH7.4); *-iv)* I_D_ of the few-molecules sensing (gate incubated in HIV-1-p24 1·10^-19^M and CRP 10^-15^M in HEPES@pH7.4). The 20^th^ curve of the cycling is shown for each step of the sensing protocol. Panels **(c)** and **(d)** display the transfer characteristic curves measured under same conditions of in the above panels, but with the bare gold reference electrode. The panels on the right magnify the region of the currents measured in the high gate voltage region, where the transconductance of the device is maximized.

**
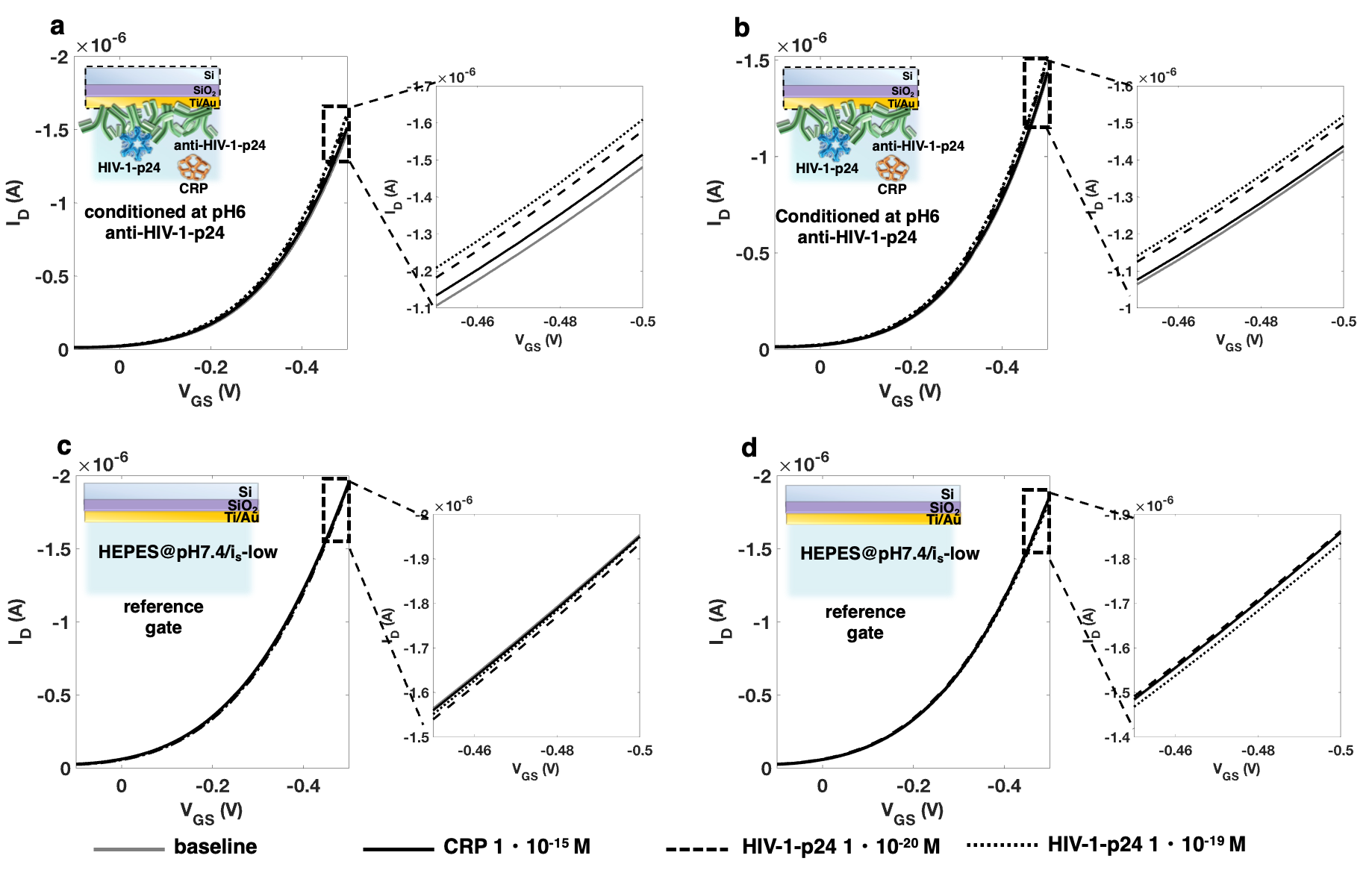
**

**Figure S27: EGOFET transfer characteristics row data for the HIV-1-p24 bioelectronic sensing at an anti-HIV-1-p24 functionalized gate conditioned at pH 6 –** In panels **(a)** and **(b)** the two replicates of the I_D_ *vs.* V_GS_ transfer characteristics (V_GS_ swiped from 0V to -0.5V while V_D_ is kept fixed at -0.4V) biased with an anti-HIV-1-p24 functionalized gate conditioned at pH 6, are displayed. The curves are measured in HEPES@7.4/i_s_-low (i_s_ = 5 mM) while all the assayed solutions are in HEPES@pH7.4. For each sample, four curves are measured, namely: *-i)* the baseline I_0_ (gate incubated in HEPES@pH7.4); *-ii)* I_D_ of the negative control experiment (gate incubated in CRP 1·10^-15^M in HEPES@pH7.4); *-iii)* I_D_ of the single-molecule sensing (gate incubated in HIV-1-p24 1·10^-20^M and CRP 10^-15^M in HEPES@pH7.4); *-iv)* I_D_ of the few-molecules sensing (gate incubated in HIV-1-p24 1·10^-19^M and CRP 10^-15^M in HEPES@pH7.4). The 20^th^ curve of the cycling is shown for each step of the sensing protocol. Panels **(c)** and **(d)** display the transfer characteristic curves measured under same conditions of in the above panels, but with the bare gold reference electrode. The panels on the right magnify the region of the currents measured in the high gate voltage region, where the transconductance of the device is maximized.


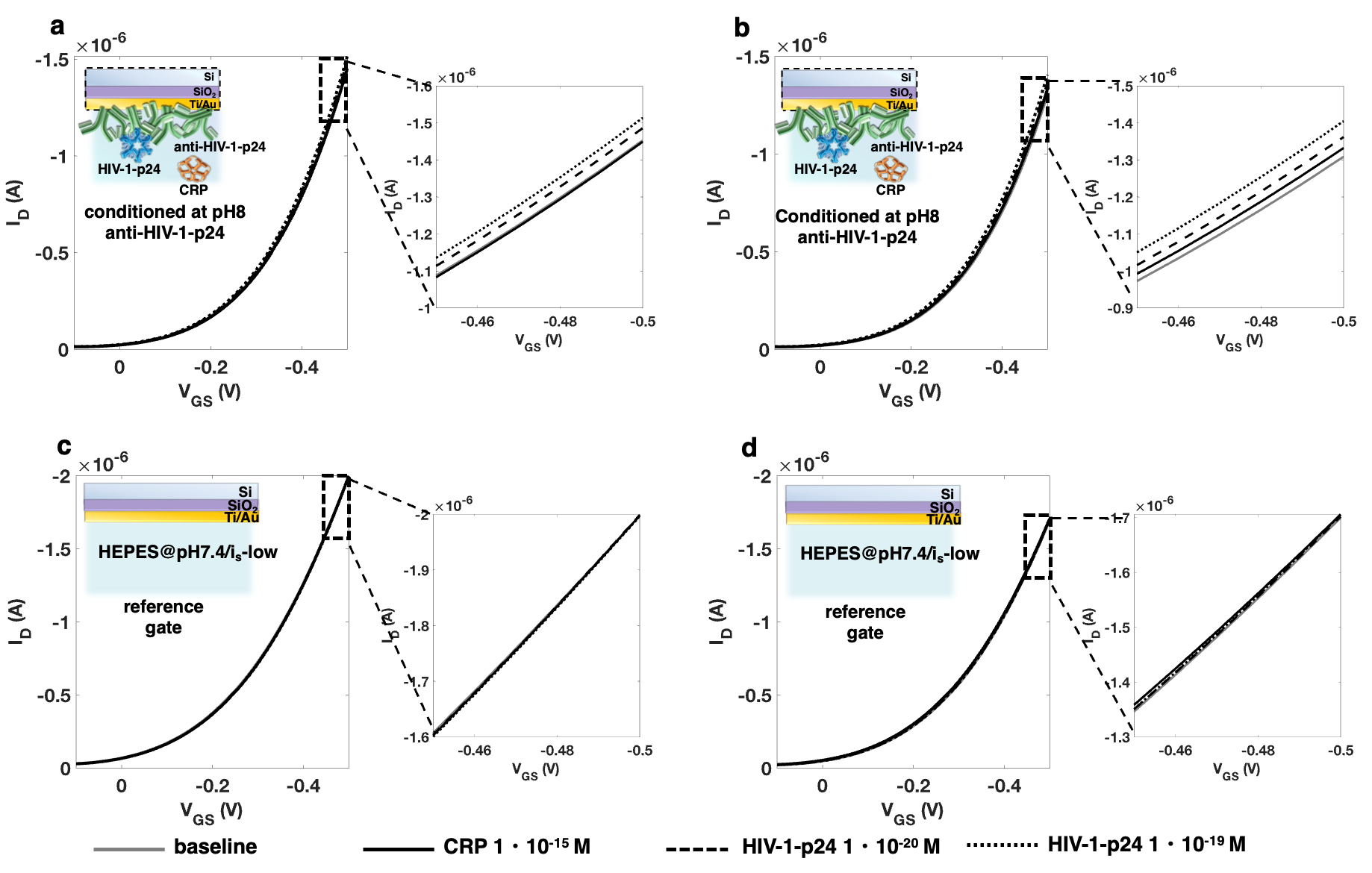


**Figure S28: EGOFET transfer characteristics row data for the HIV-1-p24 bioelectronic sensing at an anti-HIV-1-p24 functionalized gate conditioned at pH 8 -** In panels **(a)** and **(b)** the two replicates of the I_D_ *vs.* V_GS_ transfer characteristics (V_GS_ swiped from 0V to -0.5V while V_D_ is kept fixed at -0.4V) biased with an anti-HIV-1-p24 functionalized gate conditioned at pH 8, are displayed. The curves are measured in HEPES@7.4/i_s_-low (i_s_ = 5 mM) while all the assayed solutions are in HEPES@pH7.4. For each sample, four curves are measured, namely: *-i)* the baseline I_0_ (gate incubated in HEPES@pH7.4); *-ii)* I_D_ of the negative control experiment (gate incubated in CRP 1·10^-15^M in HEPES@pH7.4); *-iii)* I_D_ of the single-molecule sensing (gate incubated in HIV-1-p24 1·10^-20^M and CRP 10^-15^M in HEPES@pH7.4); *-iv)* I_D_ of the few-molecules sensing (gate incubated in HIV-1-p24 1·10^-19^M and CRP 10^-15^M in HEPES@pH7.4). The 20^th^ curve of the cycling is shown for each step of the sensing protocol. Panels **(c)** and **(d)** display the transfer characteristic curves measured under same conditions of in the above panels, but with the bare gold reference electrode. The panels on the right magnify the region of the currents measured in the high gate voltage region, where the transconductance of the device is maximized.

*Bioelectronic sensing of KRAS*

**
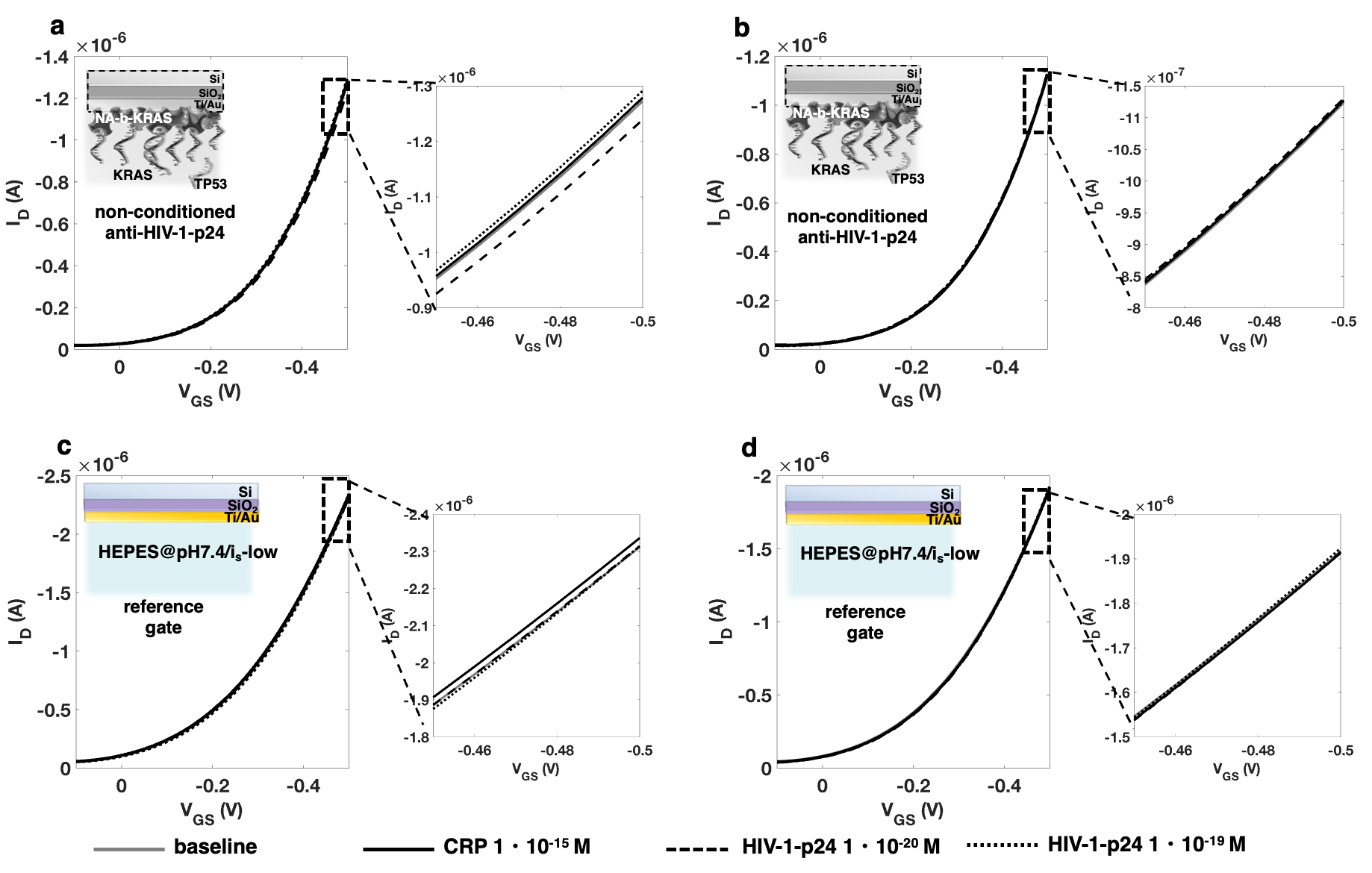
**

**Figure S29: EGOFET transfer characteristics row data for the KRAS bioelectronic sensing at a non-conditioned NA-b-KRAS functionalized gate –** In panels **(a)** and **(b)** the two replicates of the I_D_ *vs.* V_GS_ transfer characteristics (V_GS_ swiped from 0V to -0.5V while V_D_ is kept fixed at -0.4V) biased by a non-conditioned NA-b-KRAS functionalized gate, are displayed. The curves are measured in HEPES@7.4/i_s_-low (i_s_ = 5 mM) while all the assayed solutions are in HEPES@pH7.4. For each sample, four curves are measured, namely: *-i)* the baseline I_0_ (gate incubated in HEPES@pH7.4); *-ii)* I_D_ of the negative control experiment (gate incubated in TP53 1·10^-15^M in HEPES@pH7.4); *-iii)* I_D_ of the single-molecule sensing (gate incubated in KRAS 1·10^-20^M and TP53 10^-15^M in HEPES@pH7.4); *-iv)* I_D_ of the few-molecules sensing (gate incubated in KRAS 1·10^-19^M and TP53 10^-15^M in HEPES@pH7.4). The 20^th^ curve of the cycling is shown for each step of the sensing protocol. Panels **(c)** and **(d)** display the transfer characteristic curves measured under same conditions of in the above panels, but with the bare gold reference electrode. The panels on the right magnify the region of the currents measured in the high gate voltage region, where the transconductance of the device is maximized.

**
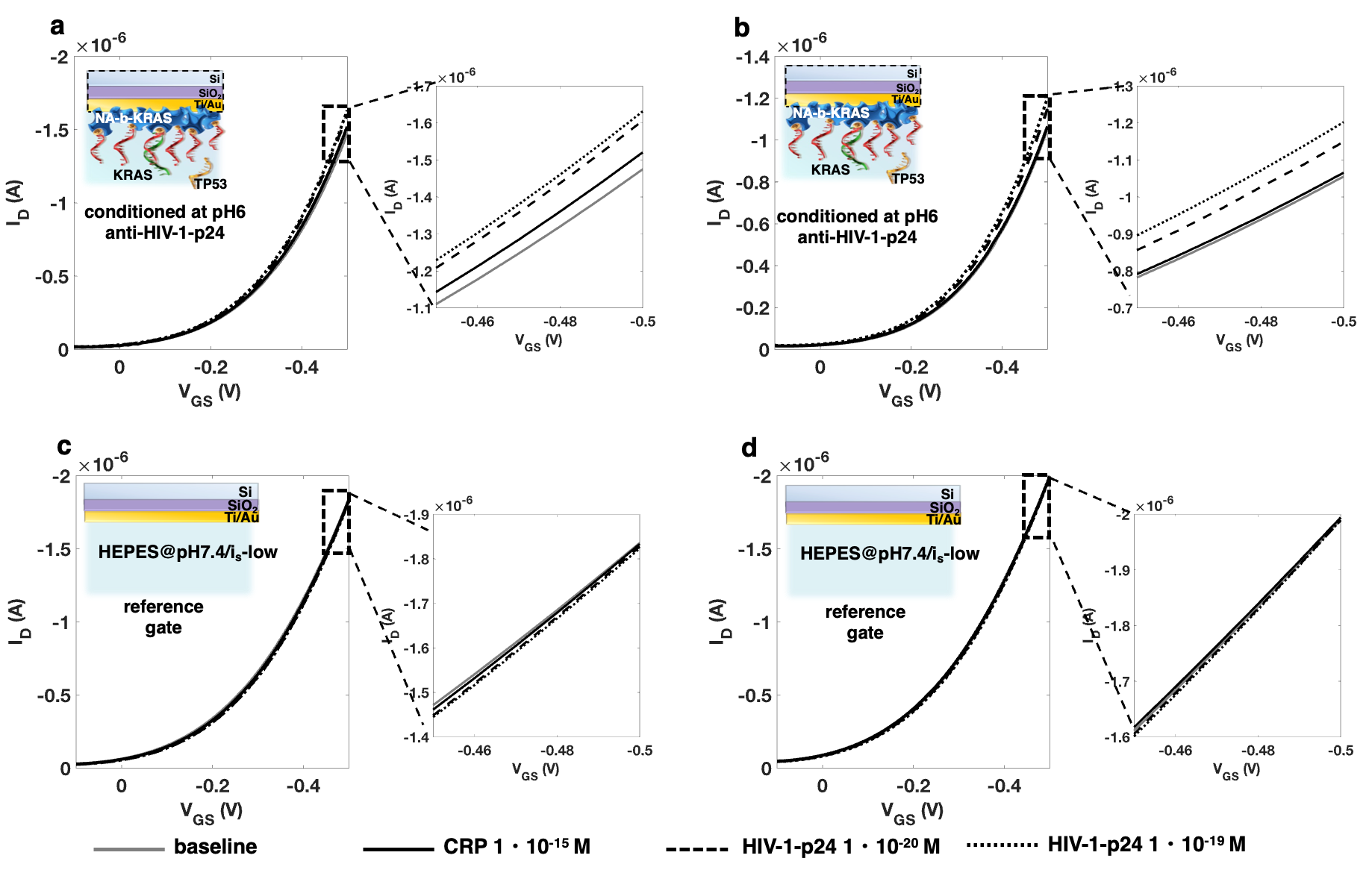
**

**Figure S30: EGOFET transfer characteristics row data for the KRAS bioelectronic sensing at a NA-b-KRAS functionalized gate conditioned at pH 6 –** In panels **(a)** and **(b)** the two replicates of the I_D_ *vs.* V_GS_ transfer characteristics (V_GS_ swiped from 0V to -0.5V while V_D_ is kept fixed at -0.4V) biased by an NA-b-KRAS functionalized gate conditioned at pH 6, are displayed. The curves are measured in HEPES@7.4/i_s_-low (i_s_ = 5 mM) while all the assayed solutions are in HEPES@pH7.4. For each sample, four curves are measured, namely: *-i)* the baseline I_0_ (gate incubated in HEPES@pH7.4); *-ii)* I_D_ of the negative control experiment (gate incubated in TP53 1·10^-15^M in HEPES@pH7.4); *-iii)* I_D_ of the single-molecule sensing (gate incubated in KRAS 1·10^-20^M and TP53 10^-15^M in HEPES@pH7.4); *-iv)* I_D_ of the few-molecules sensing (gate incubated in KRAS 1·10^-19^M and TP53 10^-15^M in HEPES@pH7.4). The 20^th^ curve of the cycling is shown for each step of the sensing protocol. Panels **(c)** and **(d)** display the transfer characteristic curves measured under same conditions of in the above panels, but with the bare gold reference electrode. The panels on the right magnify the region of the currents measured in the high gate voltage region, where the transconductance of the device is maximized.

**
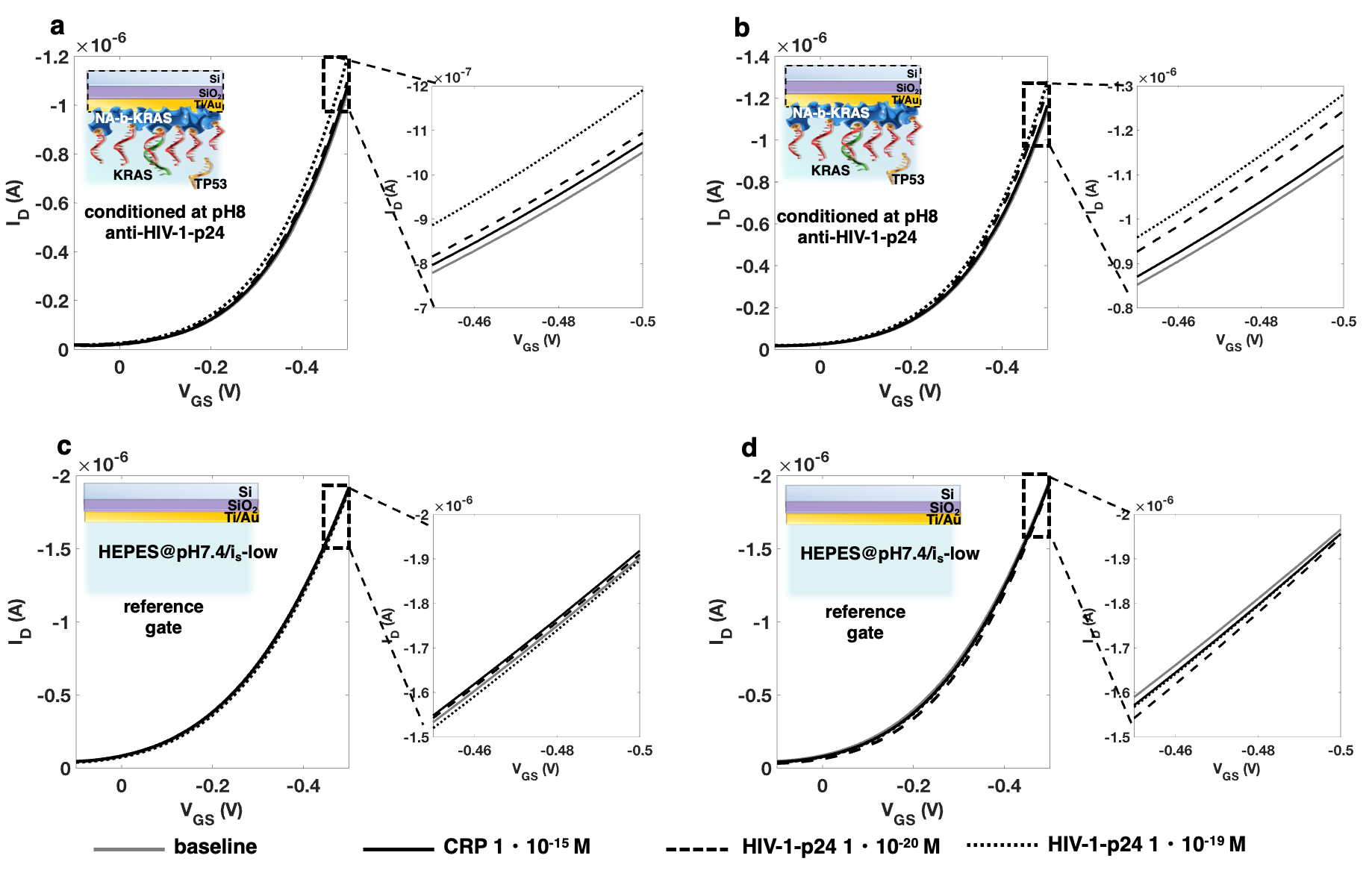
**

**Figure S31: EGOFET transfer characteristics row data fo**  **r the KRAS bioelectronic sensing at a NA-b-KRAS functionalized gate conditioned at pH 8 –** In panels **(a)** and **(b)** the two replicates of the I_D_ *vs.* V_GS_ transfer characteristics (V_GS_ swiped from 0V to -0.5V while V_D_ is kept fixed at -0.4V) biased by an NA-b-KRAS functionalized gate conditioned at pH 8, are displayed. The curves are measured in HEPES@7.4/i_s_-low (i_s_ = 5 mM) while all the assayed solutions are in HEPES@pH7.4. For each sample, four curves are measured, namely: *-i)* the baseline I_0_ (gate incubated in HEPES@pH7.4); *-ii)* I_D_ of the negative control experiment (gate incubated in TP53 1·10^-15^M in HEPES@pH7.4); *-iii)* I_D_ of the single-molecule sensing (gate incubated in KRAS 1·10^-20^M and TP53 10^-15^M in HEPES@pH7.4); *-iv)* I_D_ of the few-molecules sensing (gate incubated in KRAS 1·10^-19^M and TP53 10^-15^M in HEPES@pH7.4). The 20^th^ curve of the cycling is shown for each step of the sensing protocol. Panels **(c)** and **(d)** display the transfer characteristic curves measured under same conditions of in the above panels, but with the bare gold reference electrode. The panels on the right magnify the region of the currents measured in the high gate voltage region, where the transconductance of the device is maximized.

*A pH shift can explain also several literature papers on ultra-sensitive large-area FET biosensors*

Several studies involve ultra-sensitive biological detection using a large-area field-effect device1.

^[57,59,61–64]^ with different technologies sensing several analytes, both proteins and nucleic acid stands. Often, the very high-performance level is attributed to the amplification capabilities of FET devices. However, in a correctly performed sensing experiment, the sensing signal is to be evaluated with respect to a negative control experiment. Both need to be performed in the same setting, and hence, in a FET transduction, both are measured with the same amplification. This means that in the comparison, the advantage of the amplification is partially or totally is lost. This is the reason why the LOD level should be only marginally affected by the transistor gain. So, to explain several data from the literature the amplification effect must be sought in a binding-induced propagation effect that takes place within the biolayer of recognition elements.

In **Table S5** the experimental conditions used in several large-area FET-based sensing experiments are listed considering elements such as the transducing interface area that ranges between 10^-3^ - 10^2^ (mm)^2^. Assuming that a biofunctionalization leads to the highest packing of 10^10^ molecules/(mm)^2 [65]^ the number of recognition elements on such wide interfaces ranges between 10^7^ and 10^12^. From the lowest detected concentration (or the LOD) and the volume assayed the total number of ligands present in solution can be calculated; this figure ranges between 1 and 8·10^3^. Hence, the ratio between the maximum number of binding events (equal to the number of targets in the assayed solution) and the total number of recognition elements is computed to range between 10^4^ and 6·10^11^. Meaning that to measure a readable signal of at least 3% each binding event needs to propagate the effect of a single binding event to at least hundreds of other recognition elements. The same conclusion can be reached by considering the average footprint of a protein as 10^-10^·(mm)^2^ and compare it to the large transducing surface.

This discussion leads to the conclusion that, even from literature data tracing back to at least 2017, evidence for the necessity of an amplification effect to explain the extremely high sensitivity (in fact low LODs) in FET transduction can be found. Very relevantly, in all the manuscripts analyzed in **Table S5**, a pH change occurs when transitioning from the incubating solution to the measuring one, which is necessary to enhance the Debye length in the transistor detection. Hence, a pH shift, rather than the FET amplification or gain can explain also all these data and can be assumed to be at the basis of bioelectronic ultra-sensitivity.

**Table S5**: Detecting interfacial area dimensions and sensing protocols in large-area FET-based sensing technologies

| large-area  FET sensing technology | ref. | ligand-type | sensing  area (mm)^2^  # of recognition elements* | lowest  concentration  or LOD | # ligands | # recognition elements / ligand | incubation solution | washing /  measuring solution | is there a pH shift? |
| --- | --- | --- | --- | --- | --- | --- | --- | --- | --- |
| biofunctionalized insulator | ^[66]^ | *DNA* | *8·10^-3^*  *8·10^7^* | *6·10^-17^ M* | *4·10^2^* | *2·10^5^* | PBS buffer | Water | YES |
| biofunctionalized  gate | ^[67]^ | *RNA miR-182* | *20*  *2·10^11^* | *1·10^-20^ M* | *1* | *2·10^11^* | *PBS buffer pH 7.4* | *Water* | *YES* |
| biofunctionalized  gate | ^[54]^ | *IgG* | *60*  *6·10^11^* | *1·10^-20^ M* | *1* | *6·10^11^* | *PBS buffer pH 7.4* | *Water* | *YES* |
| biofunctionalized  gate | ^[58]^ | *IgM* | *60*  *6·10^11^* | *2.4·10^-20^ M* | *1* | *6·10^11^* | *PBS buffer pH 7.4* | *Water* | *YES* |
| biofunctionalized  gate | ^[64]^ | *HIV-1-p24* | *60*  *6·10^11^* | *3·10^-20^ M* | *2* | *6·10^11^* | *PBS buffer pH 7.4* | *Water* | *YES* |
| biofunctionalized  gate | ^[68]^ | *CRP* | *60*  *6·10^11^* | *2.5·10^-19^ M* | *15* | *6·10^11^* | *PBS buffer pH 7.4* | *Water* | *YES* |
| biofunctionalized  gate | ^[69]^ | *IgG* | *60*  *6·10^11^* | *3·10^-18^ M* | *181* | *6·10^11^* | *PBS buffer pH 7 .4* | *Water* | *YES* |
| biofunctionalized  gate | ^[70]^ | *anti-drug antibody* | *100*  *10^12^* | *1·10^-12^ M* | *6·10^7^* | *2·10^4^* | *PBS buffer* | *PBS buffer* | *N.D.* |
| biofunctionalized  gate | ^[71]^ | *α-Synuclein* | *100*  *10^12^* | *2.5·10^-13^ M* | *8·10^6^* | *1·10^5^* | *PBS buffer pH 5.5* | *PBS buffer*  *pH 7.2* | *YES* |
| biofunctionalized  gate | ^[60]^ | *spike-S SARS-Cov2* | *0.7*  *7·10^9^* | *1.8·10^-20^ M* | *2* | *3·10^9^* | *Saliva / binding buffer pH 8* | *PBS buffer*  *pH 7.4* | *YES* |
| biofunctionalized channel | ^[72]^ | *cadaverine* | *2·10^-3^*  *2·10^7^* | *1·10^-16^ M* | *6·10^3^* | *3·10^4^* | *HEPES buffer pH 8* | *PBS buffer p*  *H 7.4* | *YES* |
| biofunctionalized channel | ^[73]^ | *DNA* | *81*  *8·10^11^* | *1.7·10^-15^ M* | *4·10^4^* | *2·10^7^* | *MgCl_2_ buffer pH 8.4* | *PBS buffer*  *pH 7.4* | *YES* |
| biofunctionalized channel | ^[74]^ | *cTnI* | *4*  *4·10^10^* | *3.2·10^-14^ M* | *1·10^6^* | *4·10^4^* | *PBS buffer pH 7.4* | *Water* | *YES* |
| biofunctionalized channel | ^[75]^ | *serotonin, dopamine* | *3*  *3·10^10^* | *1·10^-13^ M* | *3·10^6^* | *10^4^* | *PBS buffer pH 7.4* | *MES buffer*  *pH 5.8* | *YES* |
| biofunctionalized channel | ^[76]^ | *Spike-S SARS-Cov2* | *1·10^-3^*  *1·10^7^* | *4·10^-18^ M* | *242* | *4·10^4^* | *UTM buffer pH 7.3* | *Water* | *YES* |
| biofunctionalized channel | ^[77]^ | *NMP22/10^8^* | *2·10^-2^*  *2·10^8^* | *2.7·10^-18^ M* | *49* | *4·10^6^* | *PBS buffer pH 7.4* | *Water* | *YES* |
| biofunctionalized extended-gate | ^[75]^ | *dopamine/10^12^* | *100*  *10^12^* | *5.2·10^-16^ M* | *3·10^2^* | *3·10^9^* | *PBS buffer pH 7 .4* | *MES buffer*  *pH 5.8* | *YES* |
| biofunctionalized extended-gate | ^[78]^ | *PSA/10^11^* | *35*  *3·10^11^* | *1·10^-15^ M* | *4·10^4^* | *7·10^6^* | *PBS buffer pH 7.4* | *PBS buffer* (*106*)*.4* | *N.D.* |
| biofunctionalized extended-gate | ^[79]^ | *PSA* | *N.D.* | *1·10^-15^ M* | *4·10^4^* | *N.D.* | *PBS buffer pH 7.4* | *Water* | *YES* |
| biofunctionalized extended-gate | ^[80]^ | *streptavidin/10^11^* | *13*  *1·10^11^* | *1·10^-13^ M* | *3·10^6^* | *3·10^4^* | *PBS buffer pH 7.4* | *PBS buffer pH 3* | *YES* |

*Assuming a density of 10^4^ recognition elements per (10^-6^m)^2^ equivalent to 10^10^ recognition elements per (mm)^2^

***SN9. Atomic Force Microscopy (AFM) - Kelvin Probe Force Microscopy (KPFM) apparatus and measurements***

*KPFM and EGOFET work function (*Φ*) and surface potential energy (*Φ_s_*) diagrams*

A scheme of the AFM/KPFM apparatus is shown in **Figure S32a**. A diode laser beam is reflected by the cantilever towards a position-sensitive photodetector (PSPD), which detects the displacement of the cantilever caused by the interaction of the cantilever tip with the sample surface. The PSPD signal recorded in different operational modes is processed by the system electronics to extract the tip-sample interaction force, or to generate images of the surface morphology. For the KPFM the conductive tip-sample contact potential difference^[81]^ is measured. To maintain a constant force or height during scanning, an electronic feedback system controls the height of the sample holder through piezoelectric scanners. Further details can be found in the Method section and elsewhere^[81,82]^.


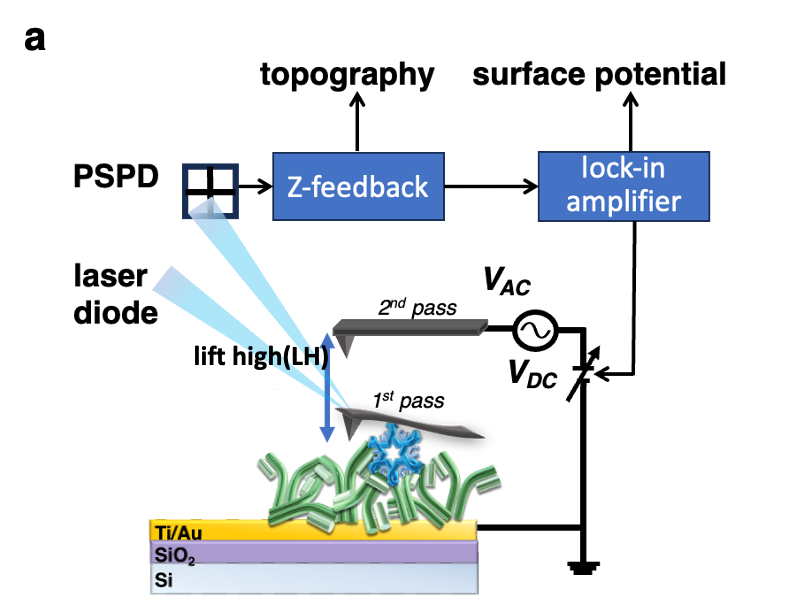


**Figure S32a. AFM/KPFM apparatus -** Schematics of two-pass AFM and Kelvin probe microscopy. The sample consists of an area covered by the biolayer of recognition elements and an area where the substrate (Au or SiO_2_) remains exposed. These two regions are separated by a sharp interface.

The surface potential signal, SP = Φ_S_/e, measured by KPFM on a biolayer is given by, SP_biolayer_ (V) = (Φ_tip_ - Φ_biolayer_)/*e*, with Φ_tip_ being the work function of the inspecting tip and *e* the elemental charge. The Au/anti-HIV-1-p24 biolayer work function, Φ_biolayer_, extracted from the measured SP_biolayer_ signal and the known Φ_tip_, is: Φ_biolayer_ (eV) = Φ_tip_ (eV) - e·SP_biolayer_ (V). On the patterned sample featuring the interface between the uncovered gold substrate and the anti-IgM layer, the surface potential measured on the gold part, SP_Au_ (V) = (Φ_tip_ - Φ_Au_)/*e*, is taken as reference. Hence the relevant Surface Potential Difference (SPD) value plotted in **Figure S32b**, is given by **Eq.**:

$SPD \left( V \right)={SP}_{biolayer}-{SP}_{Au} = \frac{\Phi_{Au -}\Phi_{biolayer}}{e}$ (**S10**).

The SPD is a very reliable parameter as it remains very stable even in the presence of surface contamination from adventitious contaminants^[81]^. In the present case $\Phi_{Au}> \Phi_{biolayer}$ therefore the SPD shifts (ΔSPD) upon physisorption of the anti-HIV-1-p24 on Au, are positive values, as it can be seen in **Figures 3A**-**3F, Figures S33a** - **S34f** and **Figures S35a** - **S36f**. The ΔSPD of the sensing with respect to the biolayer are, conversely, negative values as it can be seen in **Figures 3O** - **3Q, Figures S34m-S34q**, **Figures S34n-S34r**, **Figures S35m-S35q** and **Figures S35n-S35r**.


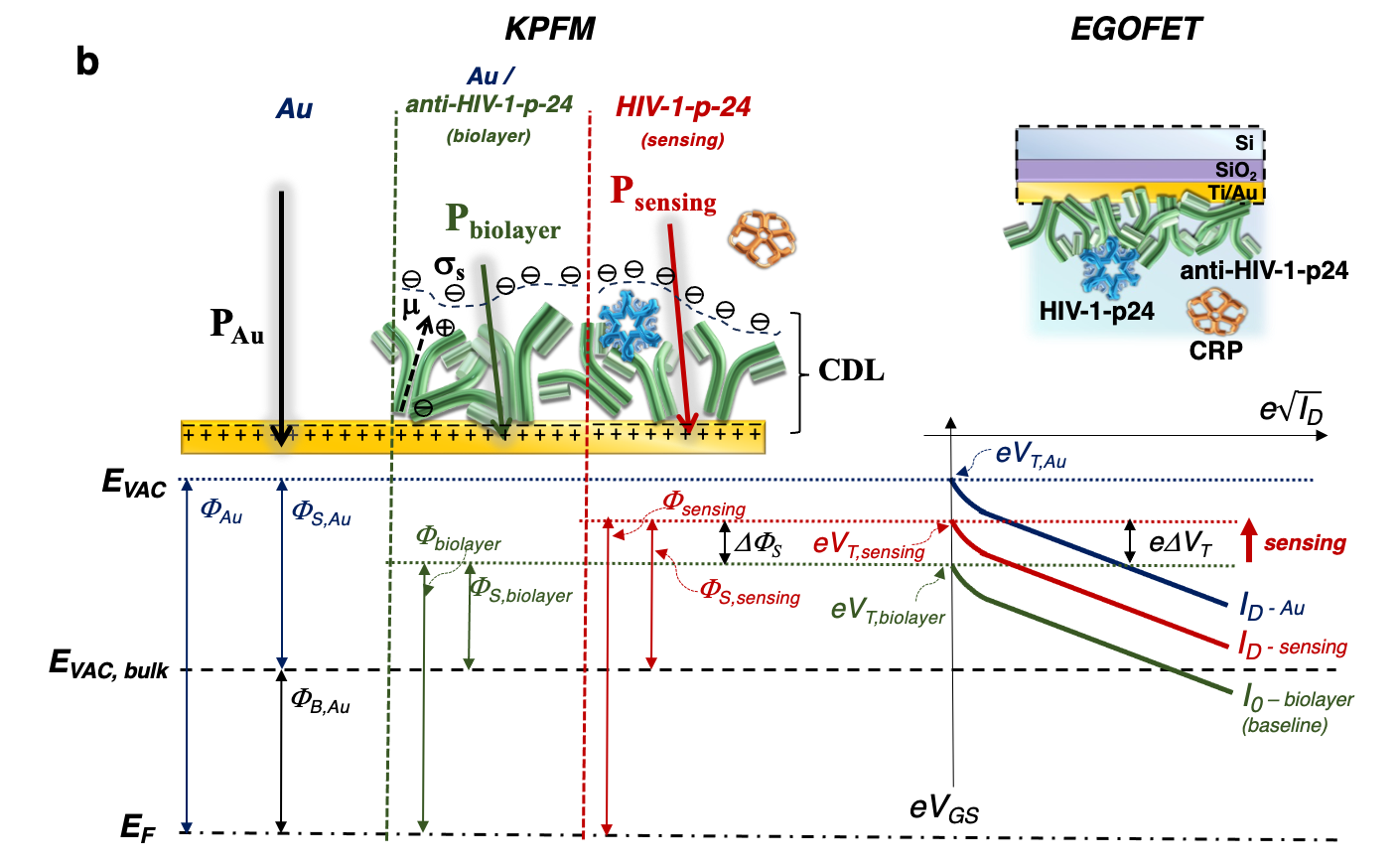


**Figure S32b. Diagram of the work functions and surface potential energies and threshold voltage shift at the different stages of the “single/few-molecules” sensing –** Energy levels at the interface between the bare gold substrate and the physisorbed capturing anti-HIV-1-p24 layer, is shown. The work function (Φ), and its surface (Φ_S_) and bulk (Φ_B_) potential components, are shown for the Au portion using the blue color code. The work function (Φ_biolayer_) and its surface potential component (Φ_S,biolayer_) of Au/anti-HIV-1-p24 biolayer is shown using a green color code. The energy shifts occurring upon HIV-1-p24 sensing (Φ_S,sensing_ and Φ_S,sensing_), are shown in red. The corresponding and *eΔV_T_* shifts for the EGOFETs are also shown on the rightmost part of the figure.

In an EGOFET (**Figure S25a**) the threshold voltage V_T_^[62]^ is taken as the flat band potential^[83]^. With a p-type semiconductor and in the common source configuration, the source (S) electrode is grounded, and the gate (G) is biased with an external voltage V_GS_. The accumulation mode regime is installed in the FET, when V_GS_, and the source-drain, V_D_, bias are both negative. In this case a negative source-drain hole current, I_D_, flows in the channel. The equation for $\sqrt{I_{D}}$ *vs.* V_GS_ at a fixed V_D_ value in the saturation region is: $\sqrt{I_{D}}=\sqrt{\frac{W\cdot\mu_{FET}\cdot C_{CDL}}{2L}}(V_{GS}-V_{T}) at \left| V_{D} \right|>\left| V_{D}^{sat} \right|$, with L and W being the transistor channel dimensions, *C_CDL_* being the gating charge-double-layer (CDL) capacitance and μ_FET_ the field-effect mobility^[84,85]^. V_T_ in an EGOFET is the gate bias that needs to be applied to form a conductive channel in the FET channel; in unintentionally p-doped organic semiconductor, such as P3HT, this occurs in the accumulation regime where V_G_ is more negative than flat-band potential, V_FB_, given by^[86]^:

$V_{FB}=V_{T}= \frac{\Phi_{biolayer -}\Phi_{P3HT}}{e}- \frac{\sigma_{s}}{C_{CDL}}$ (**S11**).

For a solution-processed P3HT a valence band edge, or equivalently a Φ_P3HT_, of 5.1 eV is reported^[87]^ which is comparable to what measured by photoemission spectroscopy as the Φ_Au_ of sputtered gold films^[88]^. If we add **Eq. S10** and **Eq. S11** and consider Φ_P3HT_ = Φ_Au_ the following holds:

$V_{T}+SPD= - \frac{\sigma_{s}}{C_{CDL}}$ (**S12**)

meaning that in the absence of surface charge V_T_ = - SPD. Differentiating **Eq. S12** leads to:

${\Delta V}_{T}+\Delta SPD= - \left( \frac{\sigma_{s}}{C_{CDL}}- \frac{\sigma_{s0}}{C_{CDL0}} \right)$ (**S13a**).

The e$\sqrt{I_{D}}$ *vs.* eV_GS_ curves are shown on the right of the energy diagram of **Figure S32b**, evidencing the graphical extraction of the V_T_ as the intercept of e$\sqrt{I_{D}}$ with eV_GS_. It is demonstrated that a shift towards more negative V_T_ measured with a p-type semiconductor channel, is seen when the gate surface potential energy becomes lower^[86]^. In the present case a threshold voltage shift, towards more negative values is seen upon biofunctionalization. The shift is reversed upon sensing and the associated energy shift eΔV_T_, confirms the trend seen with KPFM (*vide infra*).

*AFM of the biolayer*


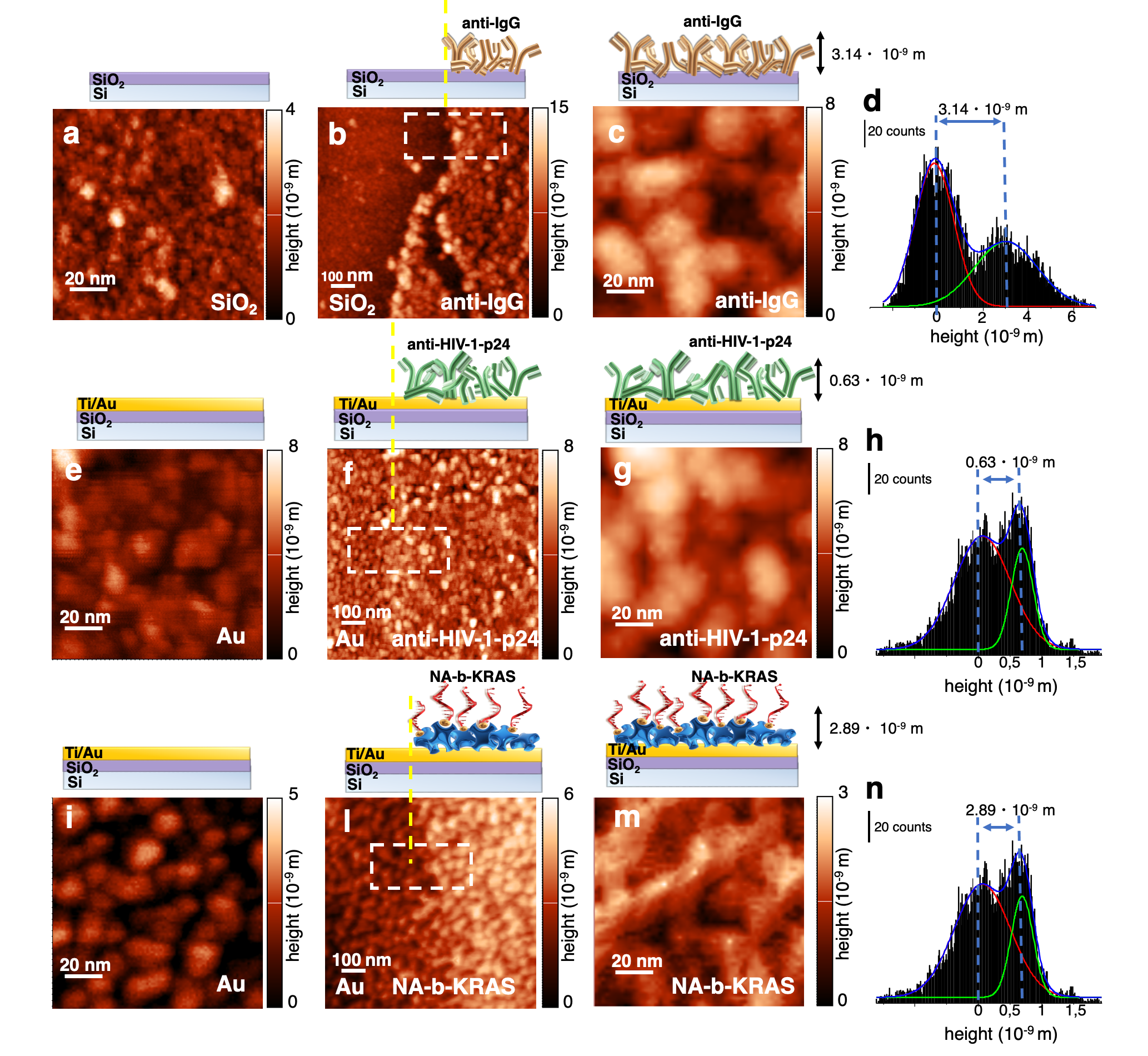


**Figure S33. AFM images of the patterned biological recognition layers: anti-IgG on SiO_2_, anti-HIV-1-p24 and NA-b-KRAS on Au -** The AFM semi-contact morphologies of the patterned samples across the interface between the substrate and the as deposited biolayers are shown. The images are acquired in air on a dried sample at room temperature (22 °C). **(a)** Representative 100 x 100 nm^2^ morphology of the SiO_2_ surface with a root mean square (RMS) roughness of 0.3 nm. **(b)** 1 x 1 µm^2^ anti-IgG physisorbed layer on the Si/SiO_2_ substrate, at the very sharp interface between the silicon oxide area and the rightmost anti-IgG region. **(c)** 100 x 100 nm^2^ AFM image of the anti-IgG layer; the morphology shows a dense network of anti-IgG clusters with 0.9 nm RMS roughness. **(d)** Histogram of the height profiles within the dashed area in **(b)**. The height difference between the biolayer and substrate, taken as reference, is 3.14 ± 0.08 nm. **(e)** Pristine gold morphology with an RMS roughness of 0.6 nm, **(f)** interface between the Au and the anti-HIV-1-p24 biofunctionalized surfaces. **(g)** anti-HIV-1-p24 biofunctionalized 100 x 100 nm^2^ image showing a net of segment-type assembly (RMS roughness of 0.8 nm). **(h)** Histogram of the height profiles within the dashed area in **(g)**. The height difference between the biolayer and the reference Au, is 0.63 ± 0.14 nm. **(i)** Au morphology image, **(l)** patterned 1 x 1 µm^2^ NA-b-KRAS layer on the Au substrate, **(m)** NA-b-KRAS assembly morphology; the size of the image is 100 x 100 nm^2^ and the RMS roughness is 0.8 nm. **(n)** Histogram of the height profiles within the dashed area in **(l)**, showing a height difference of 2.89 ± 0.16 nm. The AFM images have been flattened using a 1st order function. The histograms were built on raw data and the peaks have been fitted using Gaussian equations.

*KPFM inspection of the non-conditioned and pH conditioned anti-IgG biolayer on SiO_2_*


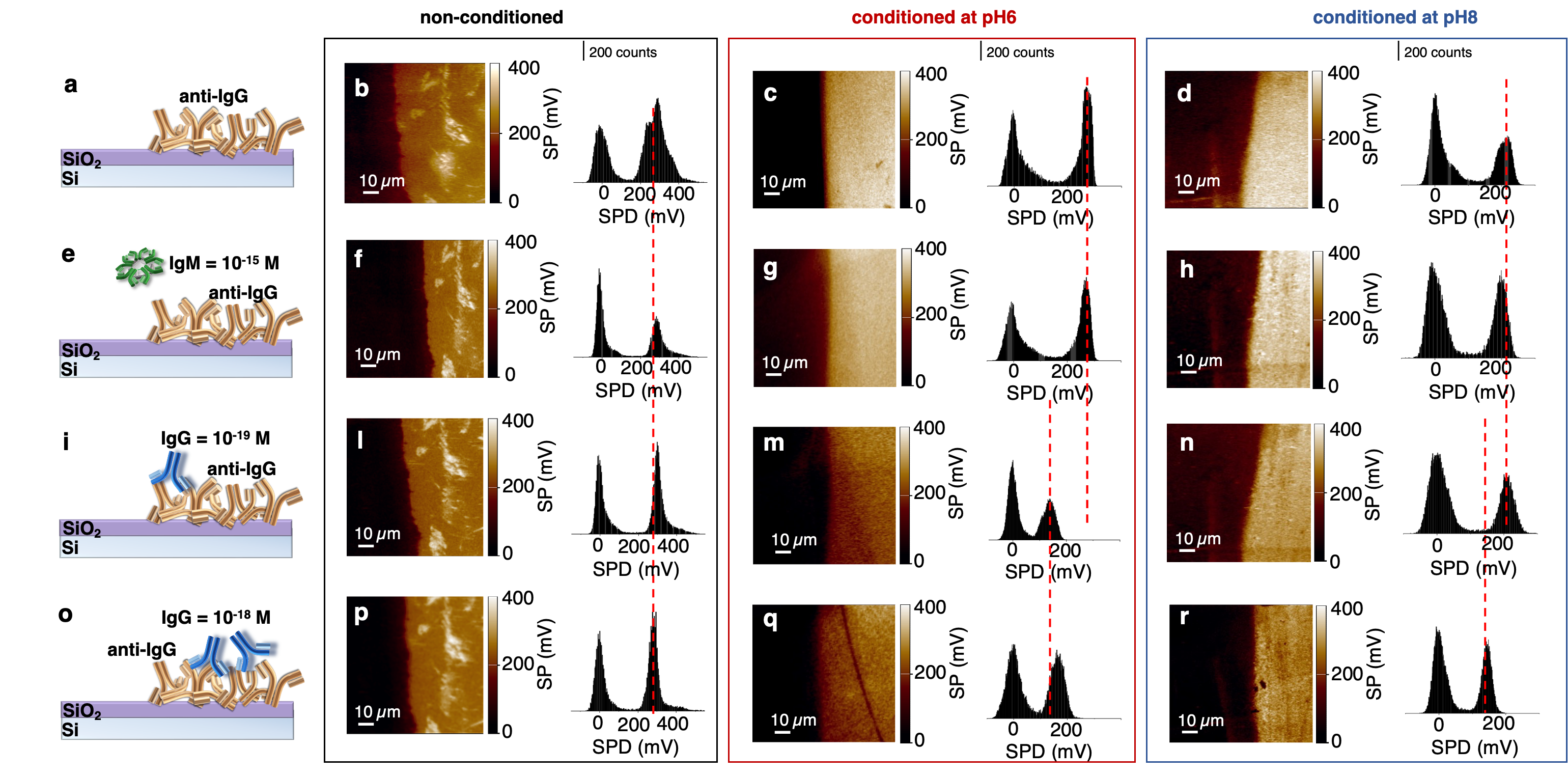


**Figure S34. Kelvin Probe Force Microscopy (KPFM) images of surface potential changes upon single/few-molecules sensing of IgG -** (**a**) Schematic representations of the patterned sample inspected across the sharp interface between the Si/SiO_2_ substrate and the physisorbed anti-IgG layer. The sample is washed in HEPES@pH7.4/i_s_-low and dried by spinning. KPFM images of a (90⋅μm)^2^ wide area are given for the non-conditioned, (**b**) the conditioned at pH 6 (**c**), and the conditioned at pH 8 (**d**) antibody capturing layer. The surface potential histograms are also shown on the rightmost panels where the surface potential difference (SPD) between the substrate (serving as internal standard) and the biolayer is plotted. Each column of panels in the figure is relevant to the KPFM analysis of the same sample at different stages of the sensing protocol. (**e**) Schematic of the sample exposed to a 1·10^-15^M HEPES@pH7.4 solution of the non-binding IgM (negative control experiment). In the {**f,g,h**} set of panels the KPFM data for the relevant anti-IgG capturing layers are shown. The samples undergo subsequent incubation in 1·10^-20^M (**i**) and 1·10^-19^M (**o**) HEPES@pH7.4 solutions of the binding IgG (sensing experiments). The related KPFM images and histograms are featured in the {**l,m,n**} and {**p,q,r**} set of panels, respectively. The red dashed lines across the histograms in each column mark the surface potential changes upon sensing.

*KPFM inspection of the non-conditioned and pH conditioned NA-b-KARAS biolayer on Au*


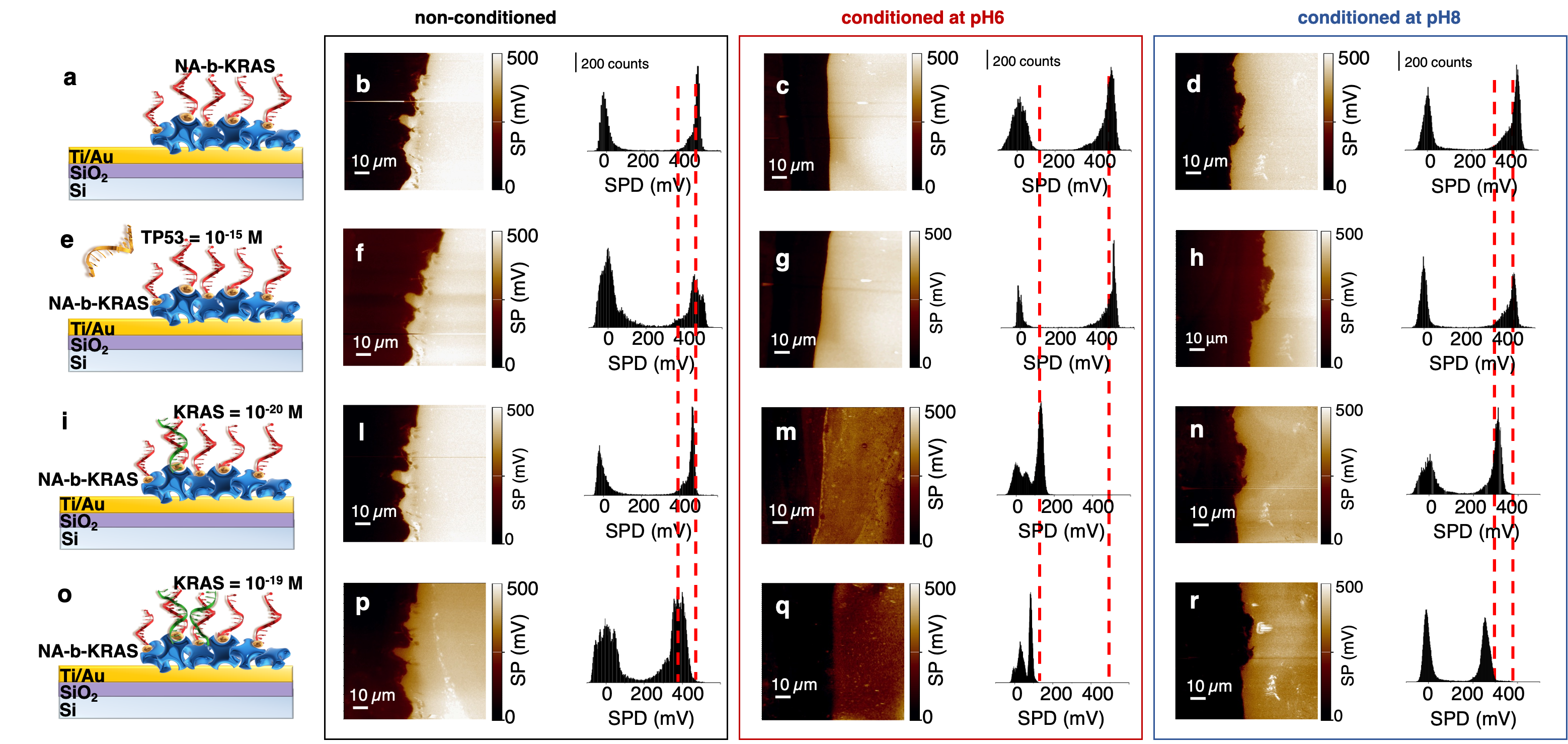


**Figure S35. Kelvin Probe Force Microscopy (KPFM) images of surface potential changes upon single/few molecules sensing of KRAS -** (**a**) Schematic representations of the patterned sample inspected across the sharp interface between the Au substrate and the physisorbed NA-b-KRAS layer. The sample is washed in HEPES@pH7.4/i_s_-low and dried by spinning. KPFM images of a 90 x 90 μm^2^ wide area are given for the non-conditioned, (**b**) the conditioned at pH 6 (**c**), and the conditioned at pH 8 (**d**) probe layer. The surface potential histograms are given on the rightmost panels where the surface potential difference (SPD) between the substrate (serving as reference) and the biolayer is plotted. Each column of panels in the figure is relevant to the KPFM analysis of the same sample at different stages of the sensing protocol. (**e**) Schematic of the sample exposed to a 1·10^-15^M HEPES@pH7.4 solution of the non-binding TP53 (negative control experiment). In the {**f,g,h**} set of panels the KPFM data for the relevant NA-b-KRAS capturing layers are shown. The samples undergo subsequent incubation in 1·10^-20^M (**i**) and 1·10^-19^M (**o**) HEPES@pH7.4 solutions of the binding KRAS (sensing experiments). The related KPFM images and histograms are featured in the {**l,m,n**} and {**p,q,r**} set of panels, respectively. The red dashed lines across the histograms in each column mark the surface potential changes induced by the sensing.

***SN10. Solid Surface Zeta Potential of the physisorbed capturing layer***

The zeta (ζ)-potential of a physisorbed anti-IgG or anti-HIV-1-p24 layer is measured with a SurPASS 3 streaming potential technique and measurements are carried out as detailed elsewhere^[89,90]^. Briefly, the ζ-potential involves measuring the current produced as an electrolyte solution flows through an adjustable gap system. In this study, an adjustable clamping cell is utilized for non-destructive analysis of rigid glass samples, 3.5 cm^2^ wide, coated with a 50 nm thick gold layer covered by physisorbed antibodies. A reusable polyvinylidene difluoride (PVDF) reference surface is also mounted in the cell. During the measurement, the capturing antibody coated glass is secured onto sample holders and placed within the cell, with the biolayer coating facing the PVDF reference surface. A gap of approximately 100 μm is set between the two surfaces. A buffered KCl electrolyte solution, with a concentration of 10 mM and pH 5.5, fills the gap, facilitating ionic conduction during the ζ-potential measurements. Before starting the measurements, thorough rinsing of both the sample and the cell with the KCl electrolyte is performed to eliminate any trapped air.


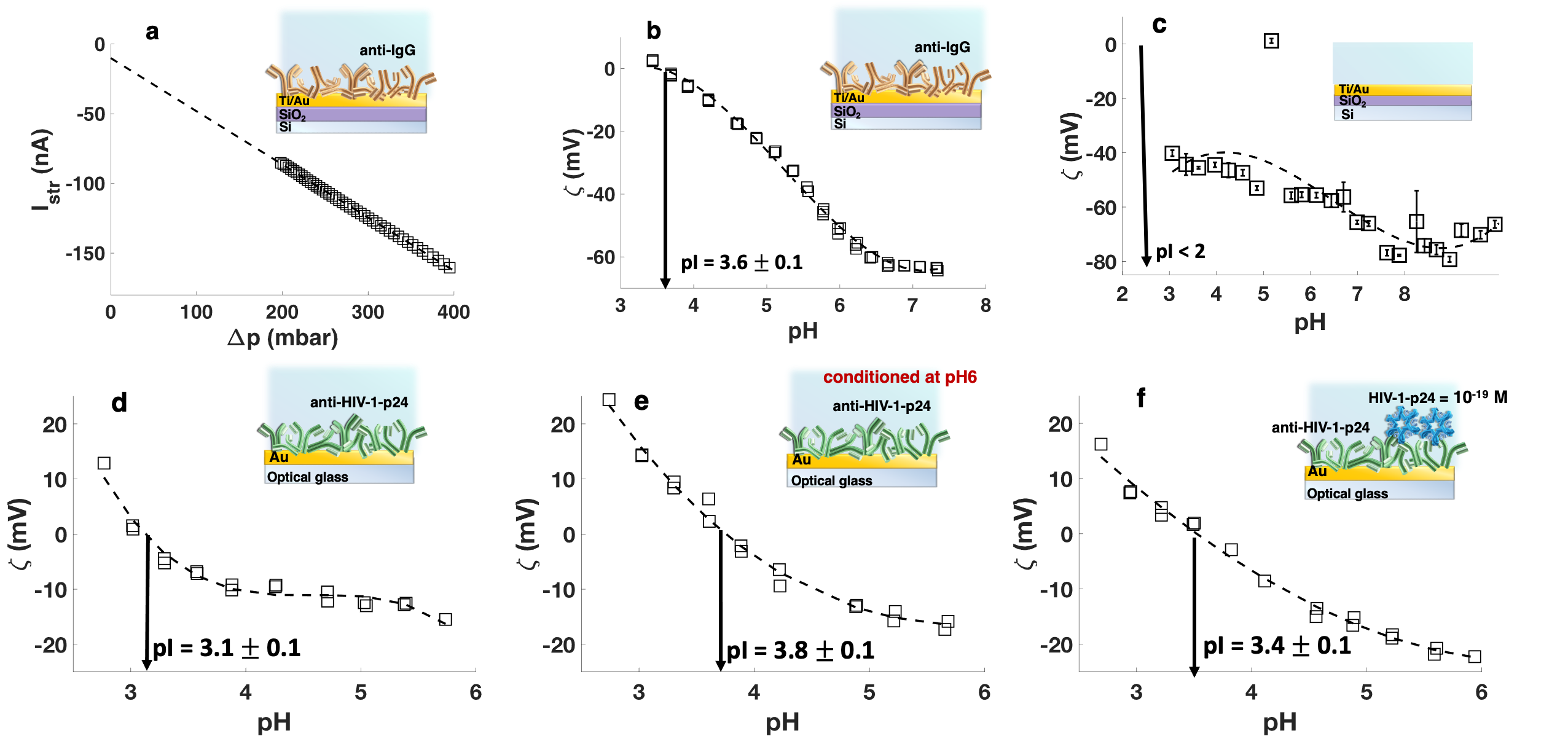


**Figure S36. ζ-potential measurements - (a)** Streaming current vs. Δp (osmotic pressure)

measured on a pristine anti-IgG sample; **(b)** Measured ζ potential v*s.* pH on a pristine anti-IgG sample and **(c)** for a plain Au surface; on each sample. Extracted ζ-potential *vs*. pH on the anti-HIV-1-p24 capturing layer pristine **(d)** conditioned at pH6 in HEPES@pH6 **(e)** and after exposure to HIV-1-p24 10^-19^ M in HEPES@pH7.5 **(f)**. Data are taken at least in duplicates and error bars are one standard deviation.

The movement of the electrolyte is induced by pressurizing the liquid reservoir using atmospheric air, leading to the separation of electrical charges in the flow's trajectory as the electrolyte passes through the measuring cell. The resulting flow of current is quantified by two Ag/AgCl electrodes allocated at the inlet and the outlet of the cell. Throughout the measurement process, both the pressure variance across the measuring cell (Δp) and the streaming current (I_str_) are monitored using a pressure control unit and an electronic circuit with minimal internal resistance, respectively. These recorded values of Δp and I_str_ are utilized to compute the ζ-potential that is given by: $\zeta=\frac{{dI}_{str}}{d\Delta p}\cdot\frac{\eta}{\varepsilon_{r}\cdot\varepsilon_{0}}\cdot\frac{L}{A}$ , with η being the viscosity, ε_r_⋅ε_0_ the dielectric permittivity, L and A the length and the cross-section of the streaming channel, respectively. The streaming current *vs.* Δp, measured on the anti-IgG sample is shown in **Figure S36a** where an excellent linearity (R^2^ = 0.9998) can be seen which is an indicator of high measurement performance and a prerequisite for a reliable measurement. The ζ-potential data extracted from **Eq. S13** and a titration curve is built as shown in **Figure S36b**. After rinsing the samples with water until equilibration, the pH titration was performed toward the isoelectric point (pI, *i.e.* the pH where ζ = 0 mV), which is indicative of the surface charge. The pI of a specific Au-anti-IgG layer is measured with a pH titration carried out adjusting the electrolyte pH with 50 mM HCl and 50 mM NaOH solutions. On the anti-IgG sample, it is registered at pI of 3.6, which means that at physiological condition the surface is negatively charged. For comparison in **Figure S36c**, the same data are given for a bare Au surface whose pI is lower than 2. In **Figure S36d-f** the same titration curve is measure for an anti-HIV-1-p24 physisorbed layer. In **Figure S36d** the pI of a pristine anti-HIV-1-p24 physisorbed layer is measured to be 3.1 ± 0.1, it shifts to 3.8 ± 0.1 (**Figure S36e**) upon conditioning at pH6, while it goes back to 3.4 ± 0.1 (**Figure S36f**) upon sensing at 10^-19^ M.

The electrokinetic potential at an interface, known as the ζ-potential, is a measurement that accounts for the charges remaining on the examined surface despite the flow of streaming current during the measurement process. Typically, the ζ-potential is linked to the composition of the Stern layer (inner Helmholtz plane) within a CDL^[91]^. This layer denotes a densely packed assembly of ions drawn from the surrounding electrolyte solution, which are electrostatically attracted to the charged surface being studied, a negatively charged gold layer in this context. Consequently, the Stern layer consists of ions tightly bound by electrostatic forces, resistant to removal by the streaming current. The ζ-potential thus serves as a gauge of the electrical potential at the interface between the Stern layer and the diffuse layer (outer Helmholtz plane) within a CDL, often termed the shear plane. While this plane is well-defined on ideally flat and chemically uniform surfaces with a Stern layer thickness within 1 nm^[92]^, a less distinct boundary is observed on surfaces biofunctionalized with protein or protein-probes layers, such as the Au/anti-IgG, Au/anti-HIV-1-p24 and the Au/NA-b-KRAS systems in this study^[93,94]^. Previous investigations have confirmed that physisorbed protein macromolecules form a tightly adherent monolayer on the gold surface. surface^[95]^. Considering the strong attachment of the protein layer to the gold surface and its resistance to removal by the streaming current, it can be inferred that in this study, the actual shear plane limit aligns with the surface of the protein or protein-probe layer.

***SN11. Electrochemical impedance spectroscopy analysis***

The Electrochemical Impedance Spectroscopy (EIS) analysis is performed to measure the capacitance of the electrode functionalized with an anti-IgG physisorbed layer at the different stages of the sensing. The data are given in **Figure S37a** for a pristine as deposited anti-IgG layer (black hollow squares), after conditioning at pH6 (gray hollow squares), and the after sensing at 10^-19^ M (red hollow squares). In **Figure S37b** the shifts of the capacitance, ΔC, after sensing with respect to the value after conditioning C_0_ (baseline in buffer), are shown.

***
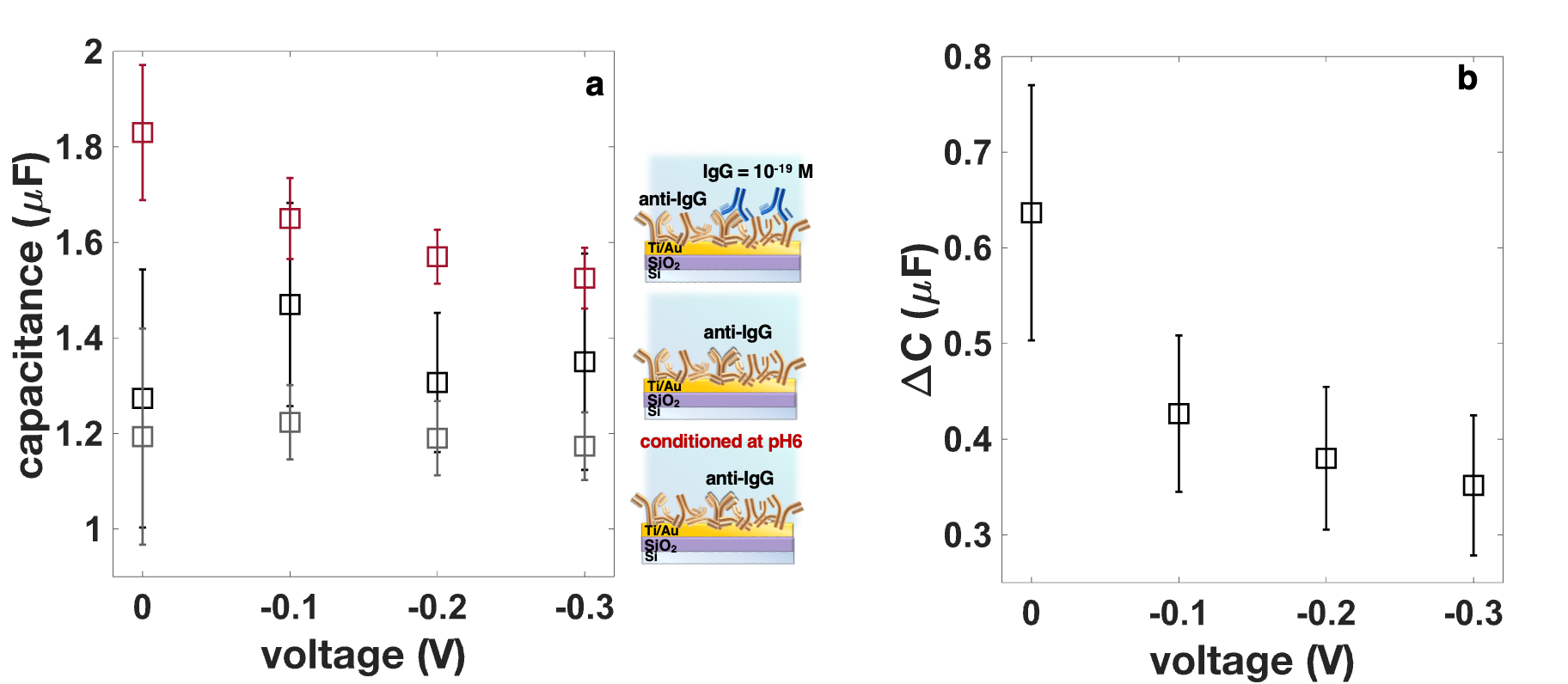
***

**Figure S37. Capacitance measurements on an anti-IgG layer - (a)** Capacitance as a function of the applied DC voltage of a pristine as deposited anti-IgG layer (black hollow squares), the layer conditioned at pH6 (gray hollow squares), and the layer after sensing at 10^-19^ M (red hollow squares). **(b)** Shift of the capacitance, ΔC, after sensing respect to the baseline C_0_ measured after conditioning at pH6. Data are acquired in triplicates and average values are plotted along with one standard deviation.

To this end, Ag/AgCl pellets served both as counter and as reference electrodes while the working electrode is the sample to be assessed. EIS are performed as a function of the DC voltage. At each operating voltage a sinusoidal signal with an amplitude of 20 mV is applied. A frequency range from 1 Hz to 10k Hz is investigated while the electrolyte solution is NaCl 10 mM at pH=7.4. The capacitance is obtained by modeling the impedance spectra with the Randless equivalent circuit. In all the investigated cases the charge transfer resistance and the Warburg element could be neglected, while the constant phase element yields the CDL capacitance. The extracted capacitance values as a function of the applied DC voltage are shown in **Figure S37a.** The capacitance slightly decreases by decreasing the applied voltage and a plateau is displayed at V = - 0.2 V, in agreement with previous published data^[54]^. The mean values of the capacitance at - 0.3 V is (1.35 ± 0.23) μF for the pristine as deposited anti-IgG layer, (1.17 ± 0.07) μF after conditioning at pH6, and (1.53 ± 0.06) μF after sensing at 10^-19^ M.

***SN12. Plasmonic and potentiometric data on “single/few-molecules” detection***

In **Table S6a** a summary the changes measured for the anti-HIV-1-p24 system with SPR (Δθ_SPR_ **Figure 1B**), EGOFET (ΔV_T_, **Figure 2B**) KPFM (ΔSPD, **Figure 3O)**, isoelectric point (pI) from ζ-potential (ΔpI and Δζ, **Figure S35c-e**) and capacitance (ΔC, **Figure S36b** at - 0.3 V) are given. For those measurements where the anti-HIV-1-p24 data are not available the responses are given for the anti-IgG system. All the shifts are computed respect to the baseline, namely the signals (θ_SPR0_, V_T0_, SPD_0_, ζ _0_ and C_0_) measured after the conditioning at pH6 in the buffer typical of the set of data. In **Table S6a** also the area of the samples inspected, the ionic strength, the pH at which the measurement is performed and the volume of the solution assayed are given.

As it is evidenced in **Figure S32b**, the biolayer physisorption lowers the Au work function, Φ_Au_, and Polarization density, **P_Au_**, as well as the negative charge at the samples surfaces, σ_s_. Hence, Φ_biolayer_ < Φ_Au_ and |**P_biolayer_|** < **|P_Au_**|. After “single/few-molecules” sensing both the **P** and the work function increases with Φ_sensing_ > Φ_biolayer_ and |**P_sensing_| > |P_biolayer_**|. After sensing, as Φ_Au_ > Φ_sensing_ , the surface is still negatively charged.

**Table S6a**: **Summary of the shifts** for “single/few-molecules” sensing with the anti-HIV-1-p24 system conditioned at pH6

|  | *plasmonic* | *potentiometric* | | | |
| --- | --- | --- | --- | --- | --- |
|  | *SPR* | *EGOFET* | *KPFM* | *ζ-potential* | *capacitance** |
| *area (cm^2^)* | 0.4 | 0.2 | 0.1 | 3.5 | 0.2 |
| *volume (mL)* | 0.1 | 0.1 | 0.3 | 0.8 | 0.1 |
| *i_s_ (mM); pH* | 150; 7.4 | 5 ; 7.4 | *in-air* | 10; 7.4 | 10; 7.4 |
|  | *SPR*  *Δθ_SPR_* | *EGOFET*  *ΔV_T_* | *KPFM*  *ΔSPD* | *ζ-potential*  Δζ | *capacitance**  *ΔC* |
| *“single-molecule”* | (8.2 ± 2.1)  10^-3^ (°) | (20 ± 2)  mV | (- 106 ± 11)  mV | -- | -- |
| *“few-molecules”* | (14.6 ± 3.4)  10^-3^ (°) | (28 ± 3)  mV | (- 91 ± 13)  mV | (31 ± 1)  mV | 0.35 ± 0.07  μF |

*^*^ measurements are taken on the anti-IgG system.*

However, to compare the data of the potentiometric measurements quantitatively it is necessary to adjust the data for the different i_s_. The EGOFET measurements are carried out at an i_s_ of 5 mM while the ζ-potential and capacitance measurements that are carried out at 10 mM. All are performed at pH 7.4. A quantitative estimate of the potential reduction, going from 5·10^-3^ M (EGOFET) to 10^-2^ M (ζ-potentials) i_s_, can be given assuming a linear relationship between the ζ-potential and the molar concentration of ions in the electrolyte solution^[96]^: ζ-potential ~ - 45 mV · ${Log}_{10}$ (i_s_). This means that the data on EGOFETs are to be lowered by 13.5 mV. For the potentiometric part, **Table S6a** becomes:

**Table S6b**: **Corrected shift values** for “few-molecules” sensing with the anti-HIV-1-p24 system conditioned at pH6

|  | *potentiometric* | | | |
| --- | --- | --- | --- | --- |
| *i_s_ (mM); pH* | 10; 7.4 | in-air (≈1 mM) | 10; 7.4 | 10; 7.4 |
|  | *EGOFET*  *ΔV_T_* | *KPFM*  *ΔSPD* | *ζ-potential*  Δζ | *capacitance**  *ΔC* |
| *“few-molecules”* | (14.5 ± 3)  mV | (- 91 ± 13)  mV | (31 ± 1)  mV | 0.35 ± 0.07  μF |

*^*^ measurements are taken on the anti-IgG system.*

Considering that the share plane in the ζ-potential is on the surface of the biolayer, we can rewrite **Eq S13a** as:

${\Delta V}_{T}+\Delta SPD= - \Delta\zeta$ (**S13b**),

and substituting the data from **Table S6b** into **Eq. S13b**, we obtain a ΔSPD = - 45.5 mV, which is shifted by - 45.5 mV, compared to the measured value of - 91 mV. This indicates that the sensing data measured in air with a KPFM tool corresponds to an i_s_ concentration approximately one decade lower than 10 mM, thus around 1 mM. Significantly, at this concentration, a Debye length, λ_D_, is comparable to the thickness for the anti-HIV-1-p24 layer.

***SN13. SCA measurements on pH-conditioned capturing layer***

In **Figure S38** the Static Contact Angle apparatus is schematically shown, while in **Figure S39** all the measurements at each sensing step are given. Here, the degree of hydrophilicity of the 1 cm^2^ capturing layer large surface after each sensing step and upon conditioning, is investigated. A rather high hydrophilic surface with an average contact angle on the two sensing steps of 41 ± 3 (°), characterizes the non-conditioned (black squares) anti-IgG biolayer, while a slightly higher contact angle of 45 ± 3 (°) is measured on the anti-IgG layer conditioned at pH 8 (blue circles). A net decrease in hydrophilicity is seen on the anti-IgG conditioned at pH 6 (red circles) already after the single-molecule sensing where an angle of 56 ± 4 (°) is measured. The static contact angle reaches 61 ± 4 (°) when the pH 6 conditioned anti-IgG is exposed to 6 ± 2 molecules of IgG. This is a 18 ± 7 (°) decrease as compared to the contact angle of 43 ± 7 (°) which is measured on the same film after the negative control experiment (IgM 1·10^-15^M).


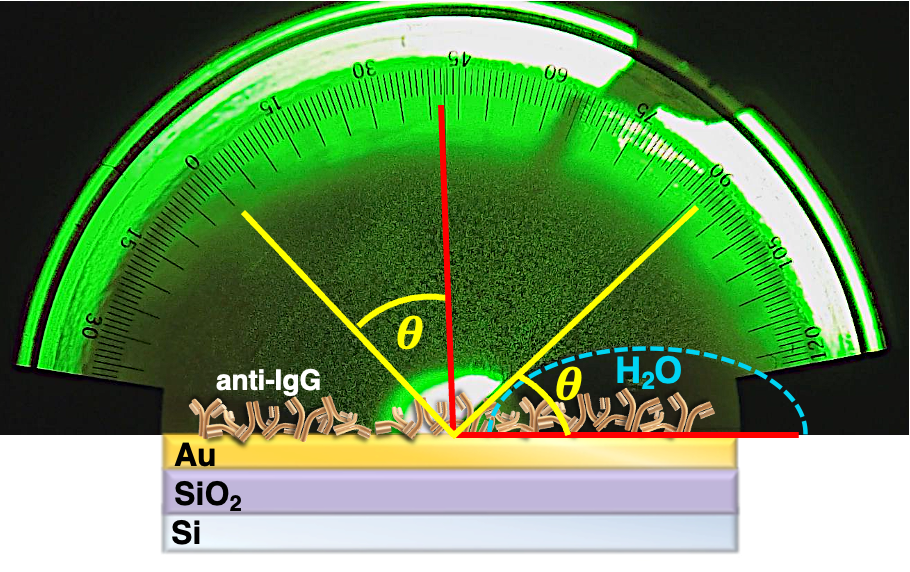


**Figure S38. Schematic representation of the static contact angle measurements -** An Au metallized Si/SiO_2_ substrate is biofunctionalized with a physisorbed anti-IgG biolayer. A 2 μL droplet of deionized water, whose contour is highlighted with a light blue dashed line, is dispensed on the 1 cm^2^ sample surface and the static contact angle, θ, is measured with a RameHart 100 goniometer while the droplet on the surface is photographed with a GoPro Macro camera. The contact angle θ is customarily taken between the sample surface (red line) and the tangent to the droplet (yellow line).


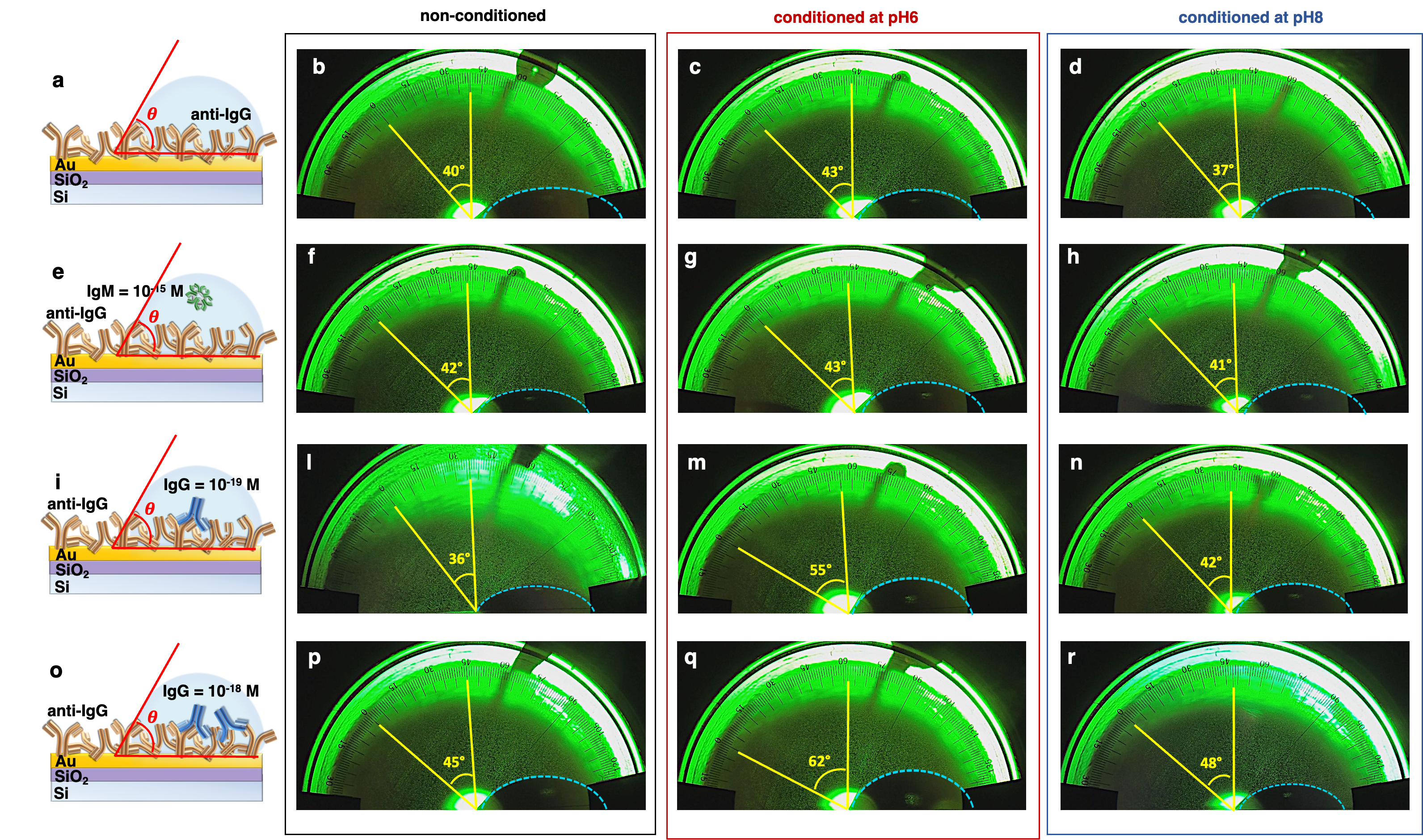


**Figure S39. Pictures of the static contact angle, θ, change upon single/few-molecules sensing of IgG -** (**a**) Schematic representations of the physisorbed anti-IgG layer on the 1 cm^2^ sample inspected. The water droplet contour is highlighted with a light blue dashed line. Images of the droplet and the relevant contact angle for the non-conditioned, (**b**) the conditioned at pH 6 (**c**), and the conditioned at pH 8 (**d**) antibody capturing layer. Each column in the figure is relevant to the contact angle assessment of the same sample at different stages of the sensing protocol. (**e**) Schematic of the sample exposed to a 1·10^-15^M HEPES@pH7.4 solution of the non-binding IgM (negative control experiment). In the {**f,g,h**} set of panels the θ values for the relevant anti-IgG capturing layers are shown. The samples undergo subsequent incubation in 1·10^-20^M (**i**) and 1·10^-19^M (**o**) HEPES@pH7.4 solutions of the binding IgG (sensing experiments). The related static contact angle images are featured in the {**l,m,n**} and {**p,q,r**} set of panels, respectively.

***SN14. Nanomechanical characterization of a conditioned anti-IgG layer***

An anti-IgG layer is physisorbed from HEPES@pH7.4 directly on Si/SiO_2_ and exposed to the non-binding IgM (1·10^-15^ M) and, afterward, to IgG (1·10^-18^ M) in HEPES@pH7.4. In 0.1 mL of an IgG 1·10^-18^ M solution, there are about 10^2^ IgG molecules, while on the (50 μm)^2^ inspected surface almost 10^8^ anti-IgG are present. In between each experiment, the sample is washed in deionized water (pH ~ 5.5 and i_s_ ~ 5 μM) and dried by spin coating (3.000 rpm for 60 s) in air.


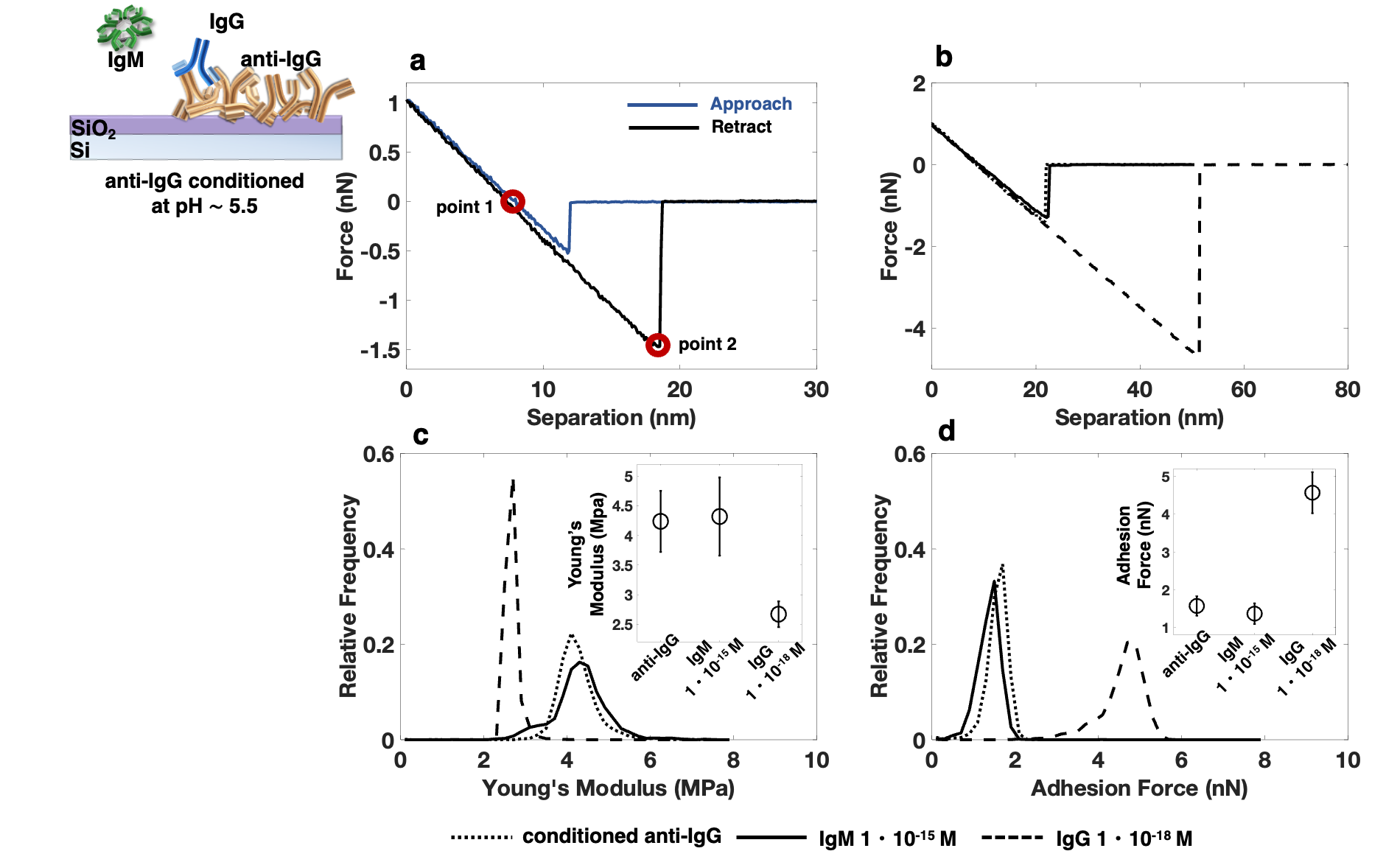


**Figure S40. AFM Force-distance spectroscopy measurements -** Representative force-distance (FD) (**a**) and retract curves (**b**). Frequency distributions, over 2.500 points, of the Young's modulus (**c**) and adhesion force (**d**) over the same (50 μm)^2^ area, where the capture anti-IgG antibody is first deposited at physiological condition and washed in water (hence it is a pH-conditioned layer), at the three stages of the experiment. Dotted line: as conditioned anti-IgG layer; Solid line: after incubation of the conditioned anti-IgG layer in IgM (1·10^-15^M in [HEPES@pH7.4](mailto:HEPES@pH7.4)) buffer solution; dashed line: after incubation of the conditioned anti-IgG layer in and IgG (1·10^-18^M in HEPES@pH7.4) buffer solution. The insets display the average Young’s modulus and adhesion force, with error bars indicating one standard deviation over three replicates on three different samples.

Force-distance spectroscopy measurements, *i.e.* the tip-surface interaction force as a function of the distance between the tip and the sample surface (FD curves) are measured by AFM on a 50 μm x 50 μm square lattice of 2.500 equally spaced points. Representative FD curves recorded as the sample approaches the tip until contact occurs (snap-in), followed by separation (blue and black curves represent the approach and retract paths, respectively), are shown in **Figure S40a**. Knowing the cantilever characteristics (deflection sensitivity, spring constant, and tip apex radius) it is possible to estimate both the adhesion force and Young's modulus of the physisorbed anti-IgG layer. Following the method proposed by Kim *et a*l.^[97]^, specific points (highlighted in **Figure S40a**) on the FD curves are used to calculate the Young’s modulus. This calculation involves mechanical models and here the Johnson-Kendall-Roberts (JKR) contact model, commonly used for soft matter characterizations, is employed. Notably, Sep_zero_ represents the distance where the force reaches zero during sample retraction (point 1), while F_min_ corresponds to the minimum force at z = Sep_min_ (point 2), indicating the adhesion force.

The representative retract curves shown in **Figure S40b** illustrate the three steps of the experiment. A wide area of 50 x 50 μm^2^ is at first inspected on the anti-IgG deposited at physiological condition and washed in deionized water, hence it is a pH-conditioned layer. Afterward, the same area is inspected after incubation of the conditioned anti-IgG layer in IgM (1·10^-15^M in HEPES@pH7.4) and IgG (1·10^-18^M in HEPES@pH7.4) buffer solutions. While the force profiles relevant to the as conditioned anti-IgG layer (black dotted line) and to the layer exposed to IgM, (black solid line) appear quite similar, a significant increase in adhesion force is evident upon exposure to the IgG antigen (black dashed curve). In **Figures S40c** and **S40d** the frequency distributions display the Young's modulus and adhesion force at the three stages of the experiment. The insets display the average Young’s modulus and adhesion force, with error bars indicating one standard deviation over three replicates on three different samples.

The quantitative nanomechanical characterization of the immunoglobulin layer reveals a marked decrease by a factor of ~ 3 of the Young’s modulus, and a corresponding decrease, by a similar factor of the adhesion force distributions, when the functionalized surface is conditioned and exposed to a few antigens. This is associated to a protein undergoes partial denaturation as, when this happens, its three-dimensional structure is affected. When partial denaturation happens, the protein loses part of its native structure, and its Young's modulus can decrease as it becomes more flexible and less stiff^[98]^. They are more hydrophobic, hence more prone to interact, so they become stickier as well^[99,100]^.

***SN15. Spectroscopic characterization with PM-IRRAS***

*The PM-IRRAS principle*

Polarization-modulation infrared reflection-absorption spectroscopy combines the principles of IRRAS with the advantages of polarization modulation enabling measurements of IR absorption of nanometres thin samples, such as the biolayer of capturing antibodies here investigated.


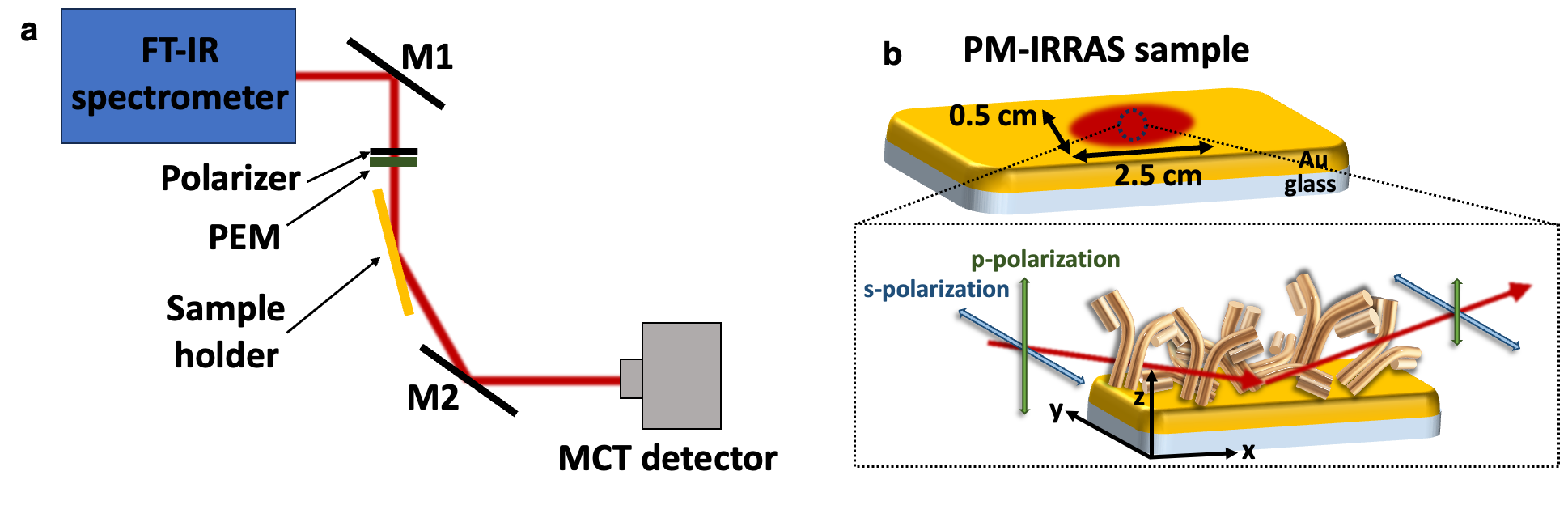


**Figure S41. Schematic of the PM-IRRAS apparatus and sample -** **(a)** Optical layout for the PM-IRRAS measurements. Infrared radiation from the FT-IR spectrometer is focused on the sample surface by a parabolic mirror (M1), passing through a wire-grid polarizer and the photoelastic modulator (PEM); then a second parabolic mirror (M2) directs radiation to the mercury cadmium telluride (MCT) detector. **(b)** PM-IRRAS sample comprises a glass slide covered with gold and functionalized with a physisorbed anti-IgG layer. The red ellipse represents the area on the sample illuminated by IR radiation. A zoom-on the anti-IgG antibody capturing layer is depicted below. The red arrows are the grazing-angle incident and reflected infrared beams with the direction of the s- and p- polarizations evidenced in blue and green, respectively. The reduction in the length of the green bar, which represents p-polarized radiation after reflection, indicates the absorption in the film. This absorption occurs only through transition dipole moments oriented along the normal to the metal surface (z-axis), as predicted by the surface selection rule at metal ad-layer interfaces^[101]^.

The experimental apparatus for PM-IRRAS measurements, schematically shown in **Figure S41**, comprises an FT-IR spectrometer, coupled with an external module containing a wire-grid polarizer, the photoelastic modulator (PEM), the sample holder and a liquid nitrogen-cooled mercury-cadmium-telluride (MCT) detector.

The investigated spectral range, 1480 – 1750 cm^-1^, corresponds to the Amide I and Amide II main IR absorption bands^.^ The Amide I band arises from the stretching vibration of the C=O bond (carbonyl group), which is particularly sensitive to the secondary structure of proteins, including the presence of α-helixes, β-sheets, turns, and random coils. The vibrational modes giving origin to the Amide II band involve the bending of the N-H and the stretching of the C-N bonds. Amide II is also sensitive to protein conformation, particularly the presence of α-helices and β-sheets and hence is often studied in conjunction with Amide I to provide complementary structural information about proteins and peptides.

The surface selection rule, valid at grazing incidence on thin film deposited on a metallic substrate, predicts that only by transition dipole moments oriented perpendicularly to the surface may contribute to the absorption^[101]^. Hence, PM-IRRAS provides insights into the orientation of the associated oscillating dipoles with respect to the normal of the substrate (z-axis) of molecules physisorbed on a metallic substrate. The contributions to the PM-IRRAS signal at a given frequency $\nu$ are proportional to the squared modulus of the z-components of the transition dipole moment vectors (**μ**)**:**

$|\left. \mu_{z} \right|^{2}=\cos^{2} \left( \theta\right) |\left. \boldsymbol{\mu} \right|^{2}$ (**S14**)

where $\theta$ is the angle between the oscillating dipole and the normal to the substrate surface. For PM-IRRAS measurements, the infrared radiation is focused on the sample surface illuminating an elliptical area of about 1 cm^2^, containing $N\sim{10}^{12}$ capturing anti-IgG, each comprising $M$ dipoles oscillating at frequency $\nu$. The intensity of the measured infrared signal, *I_IR_*, is proportional to the contributions of all transition dipole moments within the region of the biolayer exposed to the incident light,

$I_{IR}\left( \nu\right)\propto\sum_{i}^{N} \sum_{j}^{M} |\left. \mu_{zij} \right|^{2}=|\left. \mu\right|^{2}\sum_{j}^{M} \sum_{i}^{N} \cos^{2} \left( \theta_{ij} \right)$ (**S15**).

Therefore, the measured PM-IRRAS intensity depends on the distribution of the transition dipole moments orientations. Structural modifications, which involve a reorganization of antibodies in the biolayer without changing the number and nature of dipoles, can lead to an altered spectral profile. This modification may result in increased or decreased intensities, corresponding to a significant or minor alignment of dipoles along the surface normal in the z-direction.

*The PM-IRRAS multivariate data processing*

The PM-IRRAS characterization involved the analysis of 54 samples: 18 for baseline measurements (5 samples non conditioned, 9 samples conditioned at pH 6, and 4 samples conditioned at pH 8), 18 for negative control measurements (6 samples non conditioned, 6 samples conditioned at pH 6, and 6 samples conditioned at pH 8), and 18 for sensing measurements (6 samples conditioned at pH 6, and 6 samples conditioned at pH 8). Baseline, negative control, and sensing PM-IRRAS spectra were acquired in random order and on different substrate, to prevent the introduction of unwanted systematic effects in the acquisition of the PM-IRRAS signal.

**Table S7**: **Summary of the baseline, negative control and sensing** PM-IRRAS 54 spectra acquired after the conditioning at pH 6, at pH 8 and with no conditioning.

|  | Baseline | Negative control | Sensing |
| --- | --- | --- | --- |
| Not-conditioned | *5* | *6* | *6* |
| Conditioned at pH 6 | *9* | *6* | *6* |
| Conditioned at pH 8 | *4* | *6* | *6* |

The dataset summarized in **Table S7** is analyzed with multivariate data processing based on Principal Component Analysis (PCA)^[56,102]^. PCA is a statistical procedure that uses an orthogonal transformation to convert a set of correlated variables into a set of linearly uncorrelated variables called principal components (PCs). Each PC is a standardized linear combination of the original spectral frequencies (positively and negatively combined), weighted by the eigenvalues of the *loading* matrix. The *scores* are the overall summed value for each PC.

The mean-centered spectra acquired on the whole dataset in the spectral range 1480 - 1750 cm^-1^ were considered to develop the PCA model. The diagnostic on the dataset is performed utilizing the Influence plot shown in **Figure S42**, where the Q residuals *vs.* Hotelling’s T^2^ statistic is employed as a multivariate chart that allows the identification of the outliers. Specifically, Hotelling's T^2^ is a multivariate probability distribution that is a generalization of Student's t-statistic, used in multivariate hypothesis testing. It detects variation in the subspace of the first two principal components being a measure of the variation of each sample within the PCA model. Q contributions are calculated as the residuals relative to the model, thus denoting the data points that are not explained by the model of principal components. This approach enables to distinguish the data with abnormal behaviour, thanks to the statistical confidence intervals shown in the plot as red lines. The red lines in **Figure S42** define the acceptance region with a significance level of p = 0.05 (solid line), p = 0.01 (dashed line), and p = 0.001 (dotted line). The influence plot for the data set demonstrates the presence of three outliers out of 54 spectra, that were excluded from the analysis.

The results of the PCA analysis fed by the spectra, excluding the three outliers, are shown in **Figure S43**. Remarkably, the two first principal components (PCs) accounts for 98.9% of the dataset variance. The score and the loadings on the first two PCs are shown in **Figures S43a** and **S43b**, respectively. Specifically, as shown in the Score Plot reported in **Figure S43a**, PC1 explains 95.1% of the total variance. Moreover, the *loading* on PC1, shown in **Figure S43b** as a black line, is all positive and follows the profile of the signal intensity, thus suggesting that it describes conspicuous variations among samples. On the other hand, the PCA analysis reveals that baseline and negative controls’ spectra cluster in the first and second quarters of the score plot, at positive values of PC2.


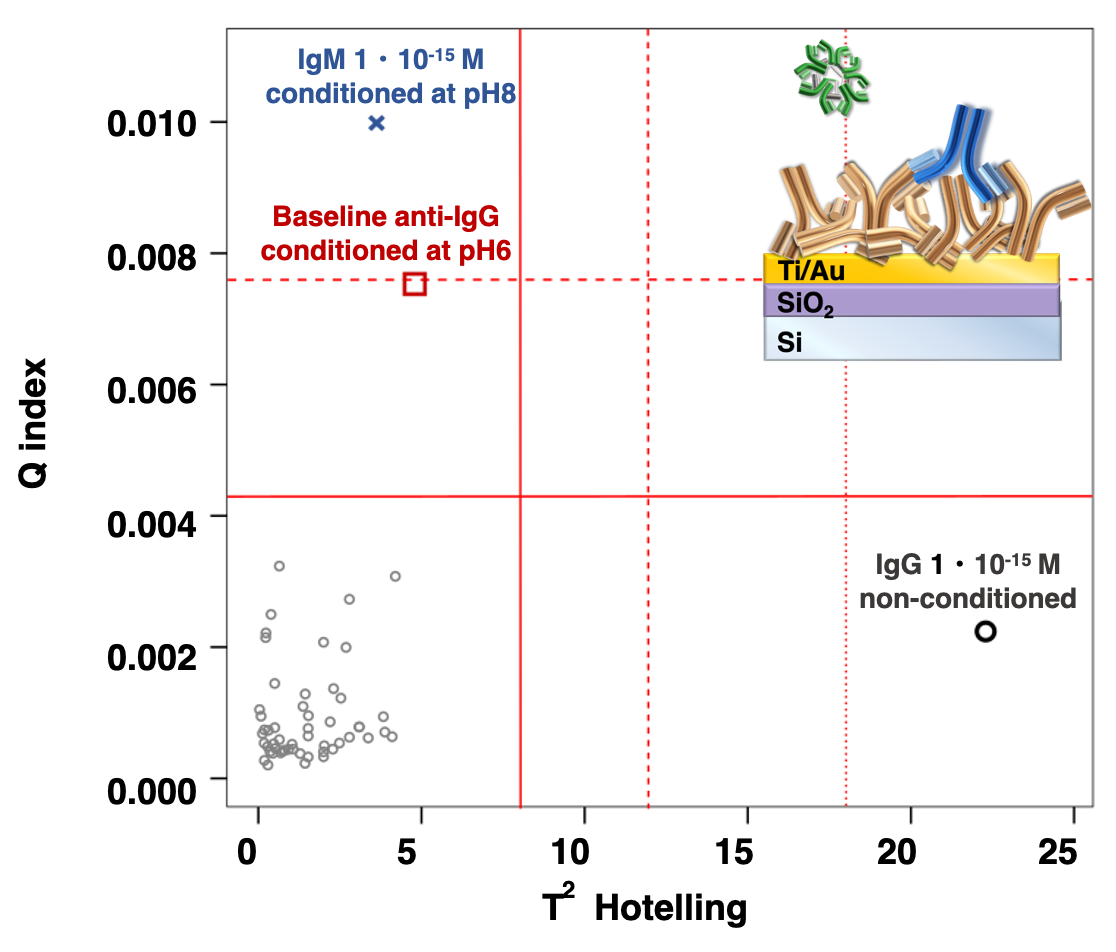


**Figure S42. Influence plot multivariate chart -** Diagnostic of the dataset is shown in the Influence plot, representing the Q residuals vs. Hotelling’s T^2^ statistic. The red lines define the acceptance regions with a significance level p=0.05 (solid line) p = 0.01 (dashed line), and p = 0.001 (dotted line).

Sensing spectra, instead, mainly cluster at negative values of PC2. Moreover, the *loading* on PC2, shown in **Figure S43b** as a gray line, suggests that the sensing spectra are characterized by increased PM-IRRAS signal at frequencies comprised in the spectral range 1660 – 1680 cm^-1^. The latter are associated to Amide I vibration. PCA further shows that the conditioning at the different pHs does not have a sizeable effect on the PM-IRRAS spectra. In **Figure S43c** three representative PM-IRRAS spectra, namely the S1 baseline (gray solid line), S2 negative control (black solid line), and S3 sensing (black dashed line) spectra highlighted in the Score plot (**Figure S43a**), are shown. It is apparent that the baseline (S1) and the negative control (S2) spectra perfectly overlap. On the other hand, the S3 spectrum revels that when the anti-IgG film is incubated with a solution containing a concentration of 1⋅10^-15^M IgG antigens the PM-IRRAS spectrum changes substantially showing an average increase of about 8% of the intensity ratio between Amide I and Amide II band areas.

This change in the S3 spectrum is explicated by the difference signal (dotted black line) between the sensing and negative control spectra, showing increased intensity between 1660 – 1680 cm^-1^.


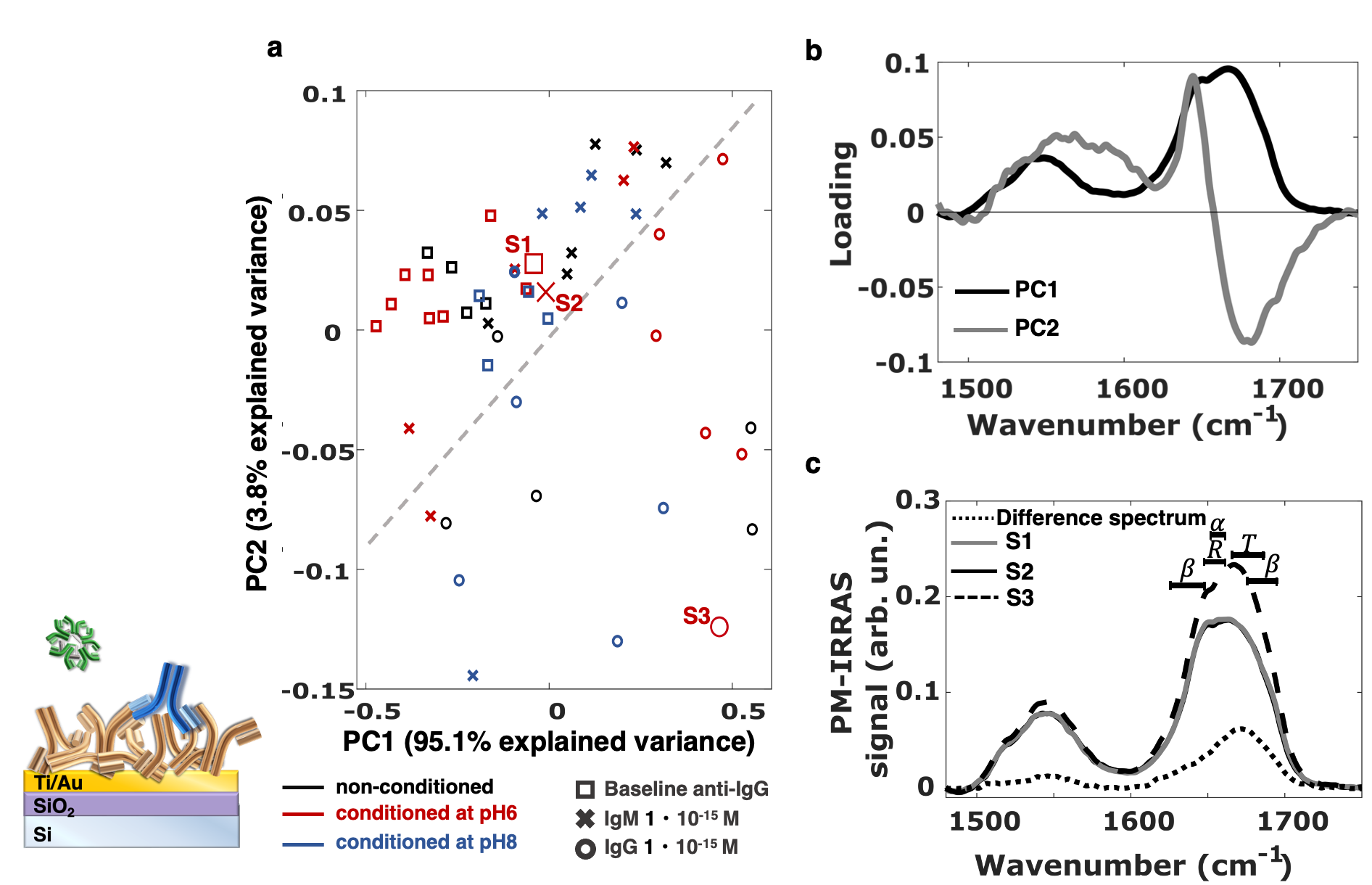


**Figure S43. Multivariate data processing of the PM-IRRAS spectra - (a)** Score-Plot featuring the score of each sample evaluated for the two principal components PC1 and PC2. The data shown as squares belong to the 17 spectra acquired with bare anti-IgG (baseline), the cross to the 17 spectra acquired upon exposure to IgM 1 fM (negative control), and the circles to the 17 spectra acquired upon exposure to IgG 1 fM (sensing experiment). The different colors identify measurements on samples that have undergone different pH shifts. The red labels refer to samples conditioned at pH 6, the blue labels those conditioned at pH 8, and the black labels the non-conditioned samples. **(b)** Loading plot, showing the loadings of each original spectral frequency in the whole inspected range (1480 - 1750 cm^-1^) on PC1 and PC2. **(c)** Spectra acquired onvecchi file the samples conditioned at pH 6 and marked as S1 (baseline), S2 (negative control), S3 (sensing) in the Score-Plot. The black dotted line refers to the difference spectrum obtained as the sensing S3 spectrum subtracted by the negative control S2 spectrum.

*Assessment of the capturing layer protonation/deprotonation before and after pH-conditioning*

The effect of pH-conditioning on as-deposited anti-IgG layers through PM-IRRAS analysis, is here assessed. The pH-conditioning protocol entails immersing a pristine anti-IgG biolayers in HEPES@pH6 or HEPES@pH8 buffer solution for 30 minutes, followed by rinsing in HEPES@pH7.4 for about 10 minutes and spin-coater drying.

In **Figure** **S44** representative PM-IRRAS spectra of non-conditioned (black-line) and conditioned biolayers (red-lines: pH6, blue lines: pH8) are shown. The spectra, cover the elicited range of 1485–1730 cm⁻¹ comprises the Amide I and the Amide II absorption bands. The Amide I band, originates as anticipated from the C=O stretching vibrations and carboxylate vibrations are sensitive to variations in protonation-state changes and hydrogen-bonding of individual amino acid side chains^[103]^. For example, the Amide I vibration of protonated carboxylic groups is expected at higher wavenumber compared to IR spectra of deprotonated residues^[104]^. The Amide II band arises from the mechanical coupling of N-H in-plane bending and C-N stretching vibrations and is significantly less sensitive to the amide group protonation. Therefore, the Amide II band has been used as an internal standard, and spectra can be normalized to the Amide II area^[105]^. As it is apparent, the Amid I peaks are not significantly different when comparing non-conditioned and pH-conditioned biolayer, thus proving that any protonation/deprotonation occurring during the exposure of the biolayer to a pH-shift is restored once the layer is exposed back to pH-7.4. At variance, the pH-conditionings do not affect the spectral responses, as confirmed also by the peak area ratios, IRRs=*I_Amide-I_/I_Amide-II_*. The non-conditioned samples exhibit an IRR= 2.59±0.05, while the biolayers conditioned at pH6 or pH8 show IRR values of (2.64 ± 0.06) and (2.62 ± 0.10), respectively. Also, these values are invariant within one standard deviation, hence in this respect the pH-conditioning is not changing the molecular secondary structure of the biolayer.

Eventually, it can be concluded that any protonation/deprotonation changes, necessarily occurring during the exposure to a pH different from 7.4, are reversible and hence are restored when the biolayer is exposed back to a physiological pH.


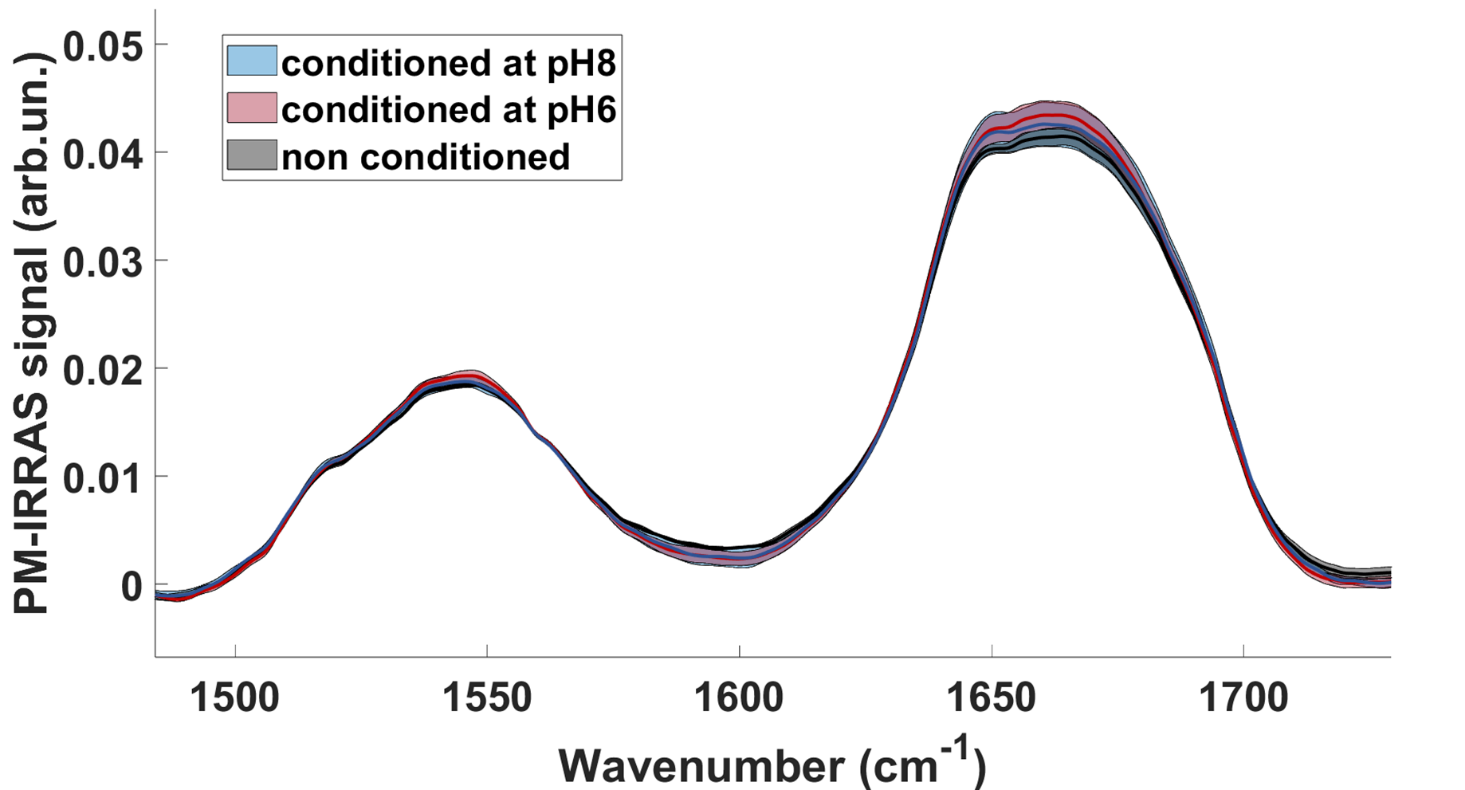


**Figure S44**. **Comparison of PM-IRRAS spectra of non-conditioned and pH-conditioned biolayer -** The non-conditioned (black-line) and pH-conditioned biolayers (red-lines: pH6, blue lines: pH8) in the spectral range of 1485–1730 cm⁻¹ are shown for the anti-IgG biolayer. The lines are the averages while colored shadings represent one standard deviation over 18 samples.

**Supporting References**

[1] J. D. Cohen, L. Li, Y. Wang, C. Thoburn, B. Afsari, L. Danilova, C. Douville, A. A. Javed, F. Wong, A. Mattox, R. H. Hruban, C. L. Wolfgang, M. G. Goggins, M. Dal Molin, T.-L. Wang, R. Roden, A. P. Klein, J. Ptak, L. Dobbyn, J. Schaefer, N. Silliman, M. Popoli, J. T. Vogelstein, J. D. Browne, R. E. Schoen, R. E. Brand, J. Tie, P. Gibbs, H.-L. Wong, A. S. Mansfield, J. Jen, S. M. Hanash, M. Falconi, P. J. Allen, S. Zhou, C. Bettegowda, L. A. Diaz, C. Tomasetti, K. W. Kinzler, B. Vogelstein, A. M. Lennon, N. Papadopoulos, *Science* 2018, *359*, 926.

[2] W. Knoll, *Annu. Rev. Phys. Chem.* 1998, *49*, 569.

[3] M. Bockovaacute, J. Slabyacute, T. Scaronpringer, J. Homola, *Annu. Rev. Anal. Chem.* 2019, *12*, 151.

[4] E. Kretschmann, H. Raether, *Z. Für Naturforschung A* 1968, *23*, 2135.

[5] R. D’Agata, N. Bellassai, G. Spoto, *Talanta* 2024, *266*, 125033.

[6] J. Homola, In *Springer Series on Chemical Sensors and Biosensensors*, 2006, pp. 3–44.

[7] J. Homola, *Chem. Rev.* 2008, *108*, 462.

[8] Q. Su, C. Jiang, D. Gou, Y. Long, *ACS Appl. Bio Mater.* 2021, *4*, 4684.

[9] Suherman, M. M. Hossain, K. Morita, T. Kawaguchi, *Orient. J. Chem.* 2018, *34*, 1355.

[10] M. Thompson, S. L. R. Ellison, R. Wood, *Pure Appl. Chem.* 2002, *74*, 835.

[11] P. Subramanian, A. Lesniewski, I. Kaminska, A. Vlandas, A. Vasilescu, J. Niedziolka-Jonsson, E. Pichonat, H. Happy, R. Boukherroub, S. Szunerits, *Biosens. Bioelectron.* 2013, *50*, 239.

[12] Y. Uludag, I. E. Tothill, *Anal. Chem.* 2012, *84*, 5898.

[13] L. Sarcina, G. F. Mangiatordi, F. Torricelli, P. Bollella, Z. Gounani, R. Österbacka, E. Macchia, L. Torsi, *Biosensors* 2021, *11*, 180.

[14] L. Sarcina, C. Scandurra, C. D. Franco, M. Caputo, M. Catacchio, P. Bollella, G. Scamarcio, E. Macchia, L. Torsi, *J. Mater. Chem. C* 2023, *11*, 9093.

[15] D. Daems, J. Lu, F. Delport, N. Mariën, L. Orbie, B. Aernouts, I. Adriaens, T. Huybrechts, W. Saeys, D. Spasic, J. Lammertyn, *Anal. Chim. Acta* 2017, *950*, 1.

[16] S. Shi, L. Wang, A. Wang, R. Huang, L. Ding, R. Su, W. Qi, Z. He, *J. Mater. Chem. C* 2016, *4*, 7554.

[17] L. Zhang, H. Wang, H. Zhang, N. Zhang, X. Zheng, W. Li, X. Qiu, D. Yu, *Sens. Actuators B Chem.* 2022, *369*, 132272.

[18] B. Ramakrishna, V. V. R. Sai, *Sens. Actuators B Chem.* 2016, *226*, 184.

[19] J. Lu, D. Spasic, F. Delport, T. Van Stappen, I. Detrez, D. Daems, S. Vermeire, A. Gils, J. Lammertyn, *Anal. Chem.* 2017, *89*, 3664.

[20] S. Lisi, S. Scarano, S. Fedeli, E. Pascale, S. Cicchi, C. Ravelet, E. Peyrin, M. Minunni, *Biosens. Bioelectron.* 2017, *93*, 289.

[21] M. Miyashita, T. Shimada, H. Miyagawa, M. Akamatsu, *Anal. Bioanal. Chem.* 2005, *381*, 667.

[22] J. Martinez-Perdiguero, A. Retolaza, L. Bujanda, S. Merino, *Talanta* 2014, *119*, 492.

[23] Y. Liu, Q. Cheng, *Anal. Chem.* 2012, *84*, 3179.

[24] J.-W. Choi, D.-Y. Kang, Y.-H. Jang, H.-H. Kim, J. Min, B.-K. Oh, *Colloids Surf. Physicochem. Eng. Asp.* 2008, *313–314*, 655.

[25] H. R. Culver, M. E. Wechsler, N. A. Peppas, *ACS Nano* 2018, *12*, 9342.

[26] G. Ma, Z. Wan, Y. Yang, W. Jing, S. Wang, *ACS Sens.* 2021, *6*, 4234.

[27] Winspall 3.02 software Wolfgang Knoll group - Max Planck Institute for Polymer Research, Germany, .

[28] Refractive Index reference value - https://refractiveindex.info/, .

[29] L. S. Jung, C. T. Campbell, T. M. Chinowsky, M. N. Mar, S. S. Yee, *Langmuir* 1998, *14*, 5636.

[30] T. L. McMeekin, M. L. Groves, N. J. Hpp, NORBERT J., In *Amino Acids and Serum Proteins*, AMERICAN CHEMICAL SOCIETY, 1964, pp. 54–66.

[31] D. Khago, J. C. Bierma, K. W. Roskamp, N. Kozlyuk, R. W. Martin, *J. Phys. Condens. Matter Inst. Phys. J.* 2018, *30*, 435101.

[32] H. Zhao, P. H. Brown, P. Schuck, *Biophys. J.* 2011, *100*, 2309.

[33] V. Ball, J. J. Ramsden, *Biopolymers* 1998, *46*, 489.

[34] J. A. De Feijter, J. Benjamins, F. A. Veer, *Biopolymers* 1978, *17*, 1759.

[35] E. Macchia, Z. M. Kovács-Vajna, D. Loconsole, L. Sarcina, M. Redolfi, M. Chironna, F. Torricelli, L. Torsi, *Sci. Adv.* 2022, *8*, eabo0881.

[36] E. A. Vogler, *Biomaterials* 2012, *33*, 1201.

[37] J. J. Ramsden, *Chem. Soc. Rev.* 1995, *24*, 73.

[38] S. Ravichandran, J. Talbot, *Biophys. J.* 2000, *78*, 110.

[39] H. H. Nguyen, J. Park, S. Kang, M. Kim, *Sensors* 2015, *15*, 10481.

[40] T. Neumann, M.-L. Johansson, D. Kambhampati, W. Knoll, *Adv. Funct. Mater.* 2002, *12*, 575.

[41] J. N. Anker, W. P. Hall, O. Lyandres, N. C. Shah, J. Zhao, R. P. Van Duyne, *Nat. Mater.* 2008, *7*, 442.

[42] S.-Y. Ding, J. Yi, J.-F. Li, B. Ren, D.-Y. Wu, R. Panneerselvam, Z.-Q. Tian, *Nat. Rev. Mater.* 2016, *1*, 1.

[43] T. Špringer, Z. Krejčík, J. Homola, *Biosens. Bioelectron.* 2021, *194*, 113613.

[44] Y. Wu, R. D. Tilley, J. J. Gooding, *J. Am. Chem. Soc.* 2019, *141*, 1162.

[45] K. M. Mayer, F. Hao, S. Lee, P. Nordlander, J. H. Hafner, *Nanotechnology* 2010, *21*, 255503.

[46] P. Zijlstra, P. M. R. Paulo, M. Orrit, *Nat. Nanotechnol.* 2012, *7*, 379.

[47] H. Li, Y. Huang, G. Hou, A. Xiao, P. Chen, H. Liang, Y. Huang, X. Zhao, L. Liang, X. Feng, B.-O. Guan, *Sci. Adv.* 2019, *5*, eaax4659.

[48] E. Hifumi, N. Kubota, Y. Niimi, K. Shimizu, N. Egashira, T. Uda, *Anal. Sci. Int. J. Jpn. Soc. Anal. Chem.* 2002, *18*, 863.

[49] M. Malmqvist, *Curr. Opin. Immunol.* 1993, *5*, 282.

[50] S. K. Vashist, C. K. Dixit, B. D. MacCraith, R. O’Kennedy, *The Analyst* 2011, *136*, 4431.

[51] H. Horry, A. Maul, G. Thouand, *Sens. Actuators B Chem.* 2007, *127*, 649.

[52] R. Leardi, *Anal. Chim. Acta* 2009, *652*, 161.

[53] C. Scandurra, P. Bollella, R. Österbacka, F. Leonetti, E. Macchia, L. Torsi, *Adv. Sens. Res.* 2022, *1*, 202270002.

[54] E. Macchia, K. Manoli, B. Holzer, C. Di Franco, M. Ghittorelli, F. Torricelli, D. Alberga, G. F. Mangiatordi, G. Palazzo, G. Scamarcio, L. Torsi, *Nat. Commun.* 2018, *9*, 3223.

[55] E. Macchia, F. Torricelli, M. Caputo, L. Sarcina, C. Scandurra, P. Bollella, M. Catacchio, M. Piscitelli, C. Di Franco, G. Scamarcio, L. Torsi, *Adv. Mater.* 2023.

[56] E. Genco, F. Modena, L. Sarcina, K. Björkström, C. Brunetti, M. Caironi, M. Caputo, V. M. Demartis, C. Di Franco, G. Frusconi, L. Haeberle, P. Larizza, M. T. Mancini, R. Österbacka, W. Reeves, G. Scamarcio, C. Scandurra, M. Wheeler, E. Cantatore, I. Esposito, E. Macchia, F. Torricelli, F. A. Viola, L. Torsi, *Adv. Mater.* 2023, *35*, 2304102.

[57] E. Macchia, F. Torricelli, P. Bollella, L. Sarcina, A. Tricase, C. Di Franco, R. Österbacka, Z. M. Kovács-Vajna, G. Scamarcio, L. Torsi, *Chem. Rev.* 2022, *122*, 4636.

[58] E. Macchia, A. Tiwari, K. Manoli, B. Holzer, N. Ditaranto, R. A. Picca, N. Cioffi, C. Di Franco, G. Scamarcio, G. Palazzo, L. Torsi, *Chem. Mater.* 2019, *31*, 6476.

[59] E. Macchia, L. De Caro, F. Torricelli, C. D. Franco, G. F. Mangiatordi, G. Scamarcio, L. Torsi, *Adv. Sci.* 2022, *9*, 2104381.

[60] K. Guo, S. Wustoni, A. Koklu, E. Díaz-Galicia, M. Moser, A. Hama, A. A. Alqahtani, A. N. Ahmad, F. S. Alhamlan, M. Shuaib, A. Pain, I. McCulloch, S. T. Arold, R. Grünberg, S. Inal, *Nat. Biomed. Eng.* 2021, *5*, 666.

[61] L. Sarcina, E. Macchia, G. Loconsole, G. D’Attoma, P. Bollella, M. Catacchio, F. Leonetti, C. Di Franco, V. Elicio, G. Scamarcio, G. Palazzo, D. Boscia, P. Saldarelli, L. Torsi, *Adv. Sci.* 2022, *9*, 2203900.

[62] F. Torricelli, D. Z. Adrahtas, Z. Bao, M. Berggren, F. Biscarini, A. Bonfiglio, C. A. Bortolotti, C. D. Frisbie, E. Macchia, G. G. Malliaras, I. McCulloch, M. Moser, T.-Q. Nguyen, R. M. Owens, A. Salleo, A. Spanu, L. Torsi, *Nat. Rev. Methods Primer* 2021, *1*, 1.

[63] L. Torsi, M. Magliulo, K. Manoli, G. Palazzo, *Chem. Soc. Rev.* 2013, *42*, 8612.

[64] E. Macchia, L. Sarcina, R. A. Picca, K. Manoli, C. Di Franco, G. Scamarcio, L. Torsi, *Anal. Bioanal. Chem.* 2020, *412*, 811.

[65] M. Pichlo, S. Bungert-Plümke, I. Weyand, R. Seifert, W. Bönigk, T. Strünker, N. D. Kashikar, N. Goodwin, A. Müller, P. Pelzer, Q. Van, J. Enderlein, C. Klemm, E. Krause, C. Trötschel, A. Poetsch, E. Kremmer, U. B. Kaupp, H. G. Körschen, U. Collienne, *J. Cell Biol.* 2014, *206*, 541.

[66] Y. Liang, M. Xiao, D. Wu, Y. Lin, L. Liu, J. He, G. Zhang, L.-M. Peng, Z. Zhang, *ACS Nano* 2020, *14*, 8866.

[67] E. Macchia, K. Manoli, C. Di Franco, R. A. Picca, R. Österbacka, G. Palazzo, F. Torricelli, G. Scamarcio, L. Torsi, *ACS Sens.* 2020, *5*, 1822.

[68] E. Macchia, K. Manoli, B. Holzer, C. Di Franco, R. A. Picca, N. Cioffi, G. Scamarcio, G. Palazzo, L. Torsi, *Anal. Bioanal. Chem.* 2019, *411*, 4899.

[69] E. Macchia, P. Romele, K. Manoli, M. Ghittorelli, M. Magliulo, Z. M. Kovács-Vajna, F. Torricelli, L. Torsi, *Flex. Print. Electron.* 2018, *3*, 034002.

[70] M. Sensi, M. Berto, S. Gentile, M. Pinti, A. Conti, G. Pellacani, C. Salvarani, A. Cossarizza, C. Augusto Bortolotti, F. Biscarini, *Chem. Commun.* 2021, *57*, 367.

[71] S. Ricci, S. Casalini, V. Parkula, M. Selvaraj, G. D. Saygin, P. Greco, F. Biscarini, M. Mas-Torrent, *Biosens. Bioelectron.* 2020, *167*, 112433.

[72] J. Oh, H. Yang, G. E. Jeong, D. Moon, O. S. Kwon, S. Phyo, J. Lee, H. S. Song, T. H. Park, J. Jang, *Anal. Chem.* 2019, *91*, 12181.

[73] R. Hajian, S. Balderston, T. Tran, T. deBoer, J. Etienne, M. Sandhu, N. A. Wauford, J.-Y. Chung, J. Nokes, M. Athaiya, J. Paredes, R. Peytavi, B. Goldsmith, N. Murthy, I. M. Conboy, K. Aran, *Nat. Biomed. Eng.* 2019, *3*, 427.

[74] M. F. M. Fathil, M. K. Md Arshad, A. R. Ruslinda, S. C. B. Gopinath, M. Nuzaihan M.N., R. Adzhri, U. Hashim, H. Y. Lam, *Sens. Actuators B Chem.* 2017, *242*, 1142.

[75] S. Palit, K. Singh, B.-S. Lou, J.-L. Her, S.-T. Pang, T.-M. Pan, *Sens. Actuators B Chem.* 2020, *310*, 127850.

[76] G. Seo, G. Lee, M. J. Kim, S.-H. Baek, M. Choi, K. B. Ku, C.-S. Lee, S. Jun, D. Park, H. G. Kim, S.-J. Kim, J.-O. Lee, B. T. Kim, E. C. Park, S. I. Kim, *ACS Nano* 2020, *14*, 5135.

[77] Y. Li, B. Zeng, Y. Yang, H. Liang, Y. Yang, Q. Yuan, *Chin. Chem. Lett.* 2020, *31*, 1387.

[78] J. Yu, M. Xu, L. Liang, M. Guan, Y. Zhang, F. Yan, H. Cao, *Appl. Phys. Lett.* 2020, *116*, 123704.

[79] V. K. Tamboli, N. Bhalla, P. Jolly, C. R. Bowen, J. T. Taylor, J. L. Bowen, C. J. Allender, P. Estrela, *Anal. Chem.* 2016, *88*, 11486.

[80] J. Lee, M. J. Kim, H. Yang, S. Kim, S. Yeom, G. Ryu, Y. Shin, O. Sul, J. K. Jeong, S.-B. Lee, *IEEE Sens. J.* 2021, *21*, 178.

[81] C. Di Franco, E. Macchia, L. Sarcina, N. Ditaranto, A. Khaliq, L. Torsi, G. Scamarcio, *Adv. Mater. Interfaces* 2023, *10*, 2201829.

[82] Cinzia Di Franco, M. Piscitelli, E. Macchia, C. Scandurra, M. Catacchio, L. Torsi, G. Scamarcio, *J. Mater. Chem. C* 2023, *12*, 73.

[83] L. Kergoat, L. Herlogsson, B. Piro, M. C. Pham, G. Horowitz, X. Crispin, M. Berggren, *Proc. Natl. Acad. Sci. U. S. A.* 2012, *109*, 8394.

[84] L. Kergoat, L. Herlogsson, D. Braga, B. Piro, M. C. Pham, X. Crispin, M. Berggren, G. Horowitz, *Adv. Mater.* 2010, *22*, 2565.

[85] L. Torsi, F. Marinelli, M. D. Angione, A. Dell’Aquila, N. Cioffi, E. D. Giglio, L. Sabbatini, *Org. Electron.* 2009, *10*, 233.

[86] L. Kergoat, L. Herlogsson, B. Piro, M. C. Pham, G. Horowitz, X. Crispin, M. Berggren, *Proc. Natl. Acad. Sci.* 2012, *109*, 8394.

[87] K. Hong, S. H. Kim, A. Mahajan, C. D. Frisbie, *ACS Appl. Mater. Interfaces* 2014, *6*, 18704.

[88] W. E. Ford, D. Gao, N. Knorr, R. Wirtz, F. Scholz, Z. Karipidou, K. Ogasawara, S. Rosselli, V. Rodin, G. Nelles, F. von Wrochem, *ACS Nano* 2014, *8*, 9173.

[89] H. Bukšek, T. Luxbacher, I. Petrinić, *Acta Chim. Slov.* 2010, *57*, 700.

[90] K.-H. Yang, P. Joshi, K. B. Rodenhausen, A. V. Sumant, S. A. Skoog, R. J. Narayan, *Mater. Lett.* 2021, *295*, 129823.

[91] A. H. B. Dourado, *Electrochem* 2022, *3*, 789.

[92] M. A. Brown, A. Goel, Z. Abbas, *Angew. Chem. Int. Ed.* 2016, *55*, 3790.

[93] V. Kesler, B. Murmann, H. T. Soh, *ACS Nano* 2020, *14*, 16194.

[94] G. Palazzo, D. De Tullio, M. Magliulo, A. Mallardi, F. Intranuovo, M. Y. Mulla, P. Favia, I. Vikholm-Lundin, L. Torsi, *Adv. Mater.* 2015, *27*, 911.

[95] L. Sarcina, C. Scandurra, C. Di Franco, M. Caputo, M. Catacchio, P. Bollella, G. Scamarcio, E. Macchia, L. Torsi, *J. Mater. Chem. C* 2023, *11*, 9093.

[96] B. J. Kirby, E. F. Hasselbrink Jr., *ELECTROPHORESIS* 2004, *25*, 187.

[97] S. Kim, Y. Lee, M. Lee, S. An, S.-J. Cho, *Nanomaterials* 2021, *11*, 1593.

[98] L. R. Khoury, I. Popa, *Nat. Commun.* 2019, *10*, 5439.

[99] J. H. M. V. Gils, D. Gogishvili, J. V. Eck, R. Bouwmeester, E. V. Dijk, S. Abeln, *Bioinforma. Adv.* 2022, *2*, 1.

[100] S. N. Jamadagni, R. Godawat, S. Garde, *Annu. Rev. Chem. Biomol. Eng.* 2011, *2*, 147.

[101] B. L. Frey, R. M. Corn, S. C. Weibel, In *Handbook of Vibrational Spectroscopy*, Wiley, 2001.

[102] P. Oliveri, C. Malegori, R. Simonetti, M. Casale, *Anal. Chim. Acta* 2019, *1058*, 9.

[103] Infrared Difference Spectroscopy of Proteins: From Bands to Bonds | Chemical Reviews, .

[104] A. K. Dioumaev, *Biochem. Mosc.* 2001, *66*, 1269.

[105] Đ. Tintor, K. Ninković, J. Milošević, N. Đ. Polović, *Vib. Spectrosc.* 2024, *134*, 103726.
